# Infant Group B Streptococcal Disease Incidence and Serotypes Worldwide: Systematic Review and Meta-analyses

**DOI:** 10.1093/cid/cix656

**Published:** 2017-11-06

**Authors:** Lola Madrid, Anna C Seale, Maya Kohli-Lynch, Karen M Edmond, Joy E Lawn, Paul T Heath, Shabir A Madhi, Carol J Baker, Linda Bartlett, Clare Cutland, Michael G Gravett, Margaret Ip, Kirsty Le Doare, Craig E Rubens, Samir K Saha, Ajoke Sobanjo-ter Meulen, Johan Vekemans, Stephanie Schrag, for the Infant GBS Disease Investigator Group, Ramesh Agarwal, Andre Ricardo Araujo da Silva, Quique Bassat, James A Berkley, Ziyaad Dangor, Sangappa Dhaded, Eric Giannoni, Majeda Hammoud, Miwako Kobayahsi, Catherine O’Sullivan, Hiro Sakata, Santhanam Sridhar, Betuel Sigaúque, Greg Tyrrell, Vinod Paul

**Affiliations:** 1 ISGlobal, Barcelona Centre for International Health Research, Hospital Clinic–University of Barcelona, Spain;; 2 Maternal, Adolescent, Reproductive and Child Health Centre, London School of Hygiene & Tropical Medicine, United Kingdom;; 3 Centro de Investigação em Saúde de Manhiça, Mozambique;; 4 College of Health and Medical Sciences, Haramaya University, Dire Dawa, Ethiopia;; 5 Centre for Child and Adolescent Health, School of Social and Community Medicine, University of Bristol, United Kingdom;; 6 Chief of Health, United Nations Children’s Fund, Afghanistan;; 7 Vaccine Institute, Institute for Infection and Immunity, St George’s, University of London and St George’s University Hospitals NHS Foundation Trust, United Kingdom;; 8 Medical Research Council: Respiratory and Meningeal Pathogens Research Unit, and Department of Science and Technology/National Research Foundation: Vaccine Preventable Diseases, University of the Witwatersrand, Faculty of Health Sciences, and; 9 National Institute for Communicable Diseases, National Health Laboratory Service, Johannesburg, South Africa;; 10 Departments of Pediatrics and Molecular Virology and Microbiology, Baylor College of Medicine, Houston, Texas;; 11 Department of International Health, Johns Hopkins Bloomberg School of Public Health, Baltimore, Maryland,; 12 Global Alliance to Prevent Prematurity and Stillbirth, Seattle, Washington;; 13 Department of Obstetrics and Gynecology, University of Washington, Seattle;; 14 Department of Microbiology, Faculty of Medicine, Chinese University of Hong Kong;; 15 Centre for International Child Health, Imperial College London, United Kingdom;; 16 Department of Global Health, University of Washington, Seattle;; 17 Bangladesh Institute of Child Health, Dhaka;; 18 Bill & Melinda Gates Foundation, Seattle, Washington;; 19 World Health Organization, Geneva, Switzerland; and; 20 National Center for Immunization and Respiratory Diseases, Centers for Disease Control and Prevention, Atlanta, Georgia

**Keywords:** group B *Streptococcus*, early onset, late onset, estimate, case fatality risk

## Abstract

**Background:**

Group B *Streptococcus* (GBS) remains a leading cause of neonatal sepsis in high-income contexts, despite declines due to intrapartum antibiotic prophylaxis (IAP). Recent evidence suggests higher incidence in Africa, where IAP is rare. We investigated the global incidence of infant invasive GBS disease and the associated serotypes, updating previous estimates.

**Methods:**

We conducted systematic literature reviews (PubMed/Medline, Embase, Latin American and Caribbean Health Sciences Literature [LILACS], World Health Organization Library Information System [WHOLIS], and Scopus) and sought unpublished data regarding invasive GBS disease in infants aged 0–89 days. We conducted random-effects meta-analyses of incidence, case fatality risk (CFR), and serotype prevalence.

**Results:**

We identified 135 studies with data on incidence (n = 90), CFR (n = 64), or serotype (n = 45). The pooled incidence of invasive GBS disease in infants was 0.49 per 1000 live births (95% confidence interval [CI], .43–.56), and was highest in Africa (1.12) and lowest in Asia (0.30). Early-onset disease incidence was 0.41 (95% CI, .36–.47); late-onset disease incidence was 0.26 (95% CI, .21–.30). CFR was 8.4% (95% CI, 6.6%–10.2%). Serotype III (61.5%) dominated, with 97% of cases caused by serotypes Ia, Ib, II, III, and V.

**Conclusions:**

The incidence of infant GBS disease remains high in some regions, particularly Africa. We likely underestimated incidence in some contexts, due to limitations in case ascertainment and specimen collection and processing. Burden in Asia requires further investigation.

Group B *Streptococcus* (GBS; *Streptococcus agalactiae*) is a leading infectious cause of neonatal morbidity and mortality, well described in high-income contexts (HICs) [1–8], but less well studied in low- to middle-income contexts (LMICs) and low-income contexts (LICs) [9]. A systematic review in 2012 [9], reported an overall incidence of invasive GBS disease among infants of 0.53 per 1000 live births (95% confidence interval [CI], .41–.62), with the highest incidence in Africa (1.21 per 1000 live births), followed by the Americas (0.67 per 1000 live births) and the lowest incidence in the Western Pacific (0.15 per 1000 live births) and Southeast Asia (0.016 per 1000 live births). Although data, especially from LICs, were limited, case fatality risks (CFRs) were higher in Africa (22%) compared with the Americas (11%) or Europe (7%) [9].

Understanding the global burden of GBS disease in young infants (0–89 days), including neonates (0–27 days), is important to guide public health decision making on interventions. Many HICs have implemented intrapartum antibiotic prophylaxis (IAP), aiming to reduce early-onset GBS disease (EOGBS; days 0–6) for women with rectovaginal GBS colonization detected through microbiological screening or with clinical risk factors [10, 11]. However, this strategy will not reduce late-onset infant GBS disease (LOGBS; onset on days 7–89 of life), and in LMICs and LICs, where there are more home deliveries and women present later for delivery, IAP may be less feasible and effective than other potential strategies for prevention, such as a maternal GBS vaccine.

This article therefore aims to examine the incidence of invasive GBS disease among young infants and the associated CFR and serotypes causing GBS invasive disease (Figure 1). It is part of a supplement estimating the burden of GBS disease among pregnant women, stillbirths, and infants [12]. The supplement includes systematic reviews and meta-analyses on GBS colonization, and adverse outcomes associated with GBS around birth [10, 13–19], which provide data inputs for estimating the worldwide burden of GBS [20].

**Figure 1.**
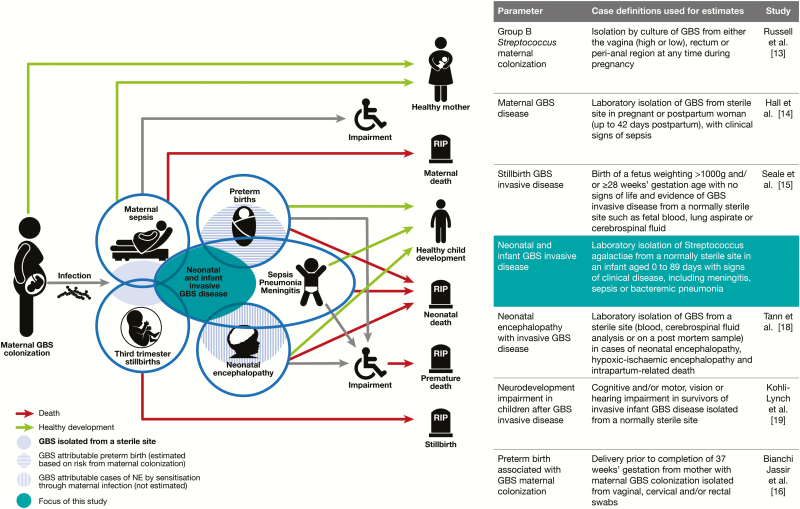
Infant group B streptococcal (GBS) disease in disease schema for GBS, as described by Lawn et al [12].

## OBJECTIVES

1. To provide a comprehensive, systematic literature review and meta-analyses on the burden of infant invasive GBS disease to include:
  a. Incidence of infant GBS disease: overall incidence risk, including stratification by EOGBS and LOGBS.
  b. CFR for EOGBS and LOGBS (7–89 days) and neonatal disease (7–27 days).
  c. Serotype distribution: prevalence of GBS serotypes causing GBS disease among infants.
2. To generate parameters to be used as data inputs in a compartmental model estimating the burden of GBS in pregnancy for women, stillbirth, and infants; including
  a. EOGBS to LOGBS ratio.
  b. Clinical syndrome (proportion of neonatal disease that was meningitis or sepsis).
3. To evaluate data gaps and recommend improvements for the data regarding GBS disease in young infants.

## METHODS

This article is part of a protocol entitled “Systematic estimates of the global burden of GBS in pregnant women, stillbirths and infants,” submitted for ethical approval to the London School of Hygiene & Tropical Medicine (reference number 11966) and approved on 30 November 2016. The general methods are described elsewhere [12]; here we present details specific to estimates related to the incidence of invasive GBS disease among infants.

We included studies that described incidence risk, deaths, or serotypes of bacterial isolates among infants aged 0–89 days with invasive GBS disease. Eligible studies were those reporting data published or unpublished between 1 January 2000 and 31 January 2017, limited to humans and with no language restrictions. We identified data through systematic review of the published literature and through an investigator group that sought unpublished data from clinicians, researchers, and relevant professional institutions worldwide.

### Definitions

Invasive GBS disease was defined as laboratory isolation of *S. agalactiae* from any normally sterile site using conventional microbiological methods together with any signs of clinical disease. EOGBS was defined as invasive GBS disease in infants aged 0–6 days after birth and LOGBS in infants 7–89 days after birth. Incidence risk was defined as cases per 1000 live births and CFR as number of deaths in GBS cases divided by total GBS cases.

### Search Strategy

We undertook systematic literature searches of PubMed/Medline, Embase, Literature in the Health Sciences in Latin America and the Caribbean (LILACS), the World Health Organization Library Information System (WHOLIS), and Scopus databases using the search terms (“*Streptococcus agalactiae*” [Medical subject headings (MeSH)] OR “*Streptococcus* Group B” OR “Group B streptococcal”) AND “infant,” “outcome,” “death,” “mortality,” “case AND fatality AND rate.” We limited searches to humans and publications from 1 January 2000 to 31 January 2017 (see Supplementary Table 1 for the full list of search terms). For consistency, we used the same search terms as a previous systematic review [9]. We did not apply date or language restrictions; texts were translated to English when published in other languages. An additional search for reports with serotype data was performed, using the search terms (“*Streptococcus agalactiae serotype*” [MeSH] OR “*Streptococcus* Group B serotype” OR “Group B streptococcal serotype”) using the same limits above. We used snowball searches of article reference lists including reviews to identify additional studies

One investigator performed the database search, screened for duplicates, and screened titles and abstracts to assess eligibility for inclusion. Two independent investigators (L. M. and M. K. L.) assessed the full-length articles associated with selected abstracts to confirm eligibility and extract data. Where there was discrepancy between the 2 reviewers, a third investigator (A. S.) made the final decision.

### Study Selection

We included studies with original data on GBS disease in infants who were aged 0–89 days at onset of infection episode, with clinical specimens obtained from a sterile site, which had a population denominator (total live births). We excluded studies focusing on very high-risk groups (such as only human immunodeficiency virus [HIV]–infected infants or only preterm infants), where data were not representative of live births in the population. Where countries had multiple or duplicated publications or systematically collected surveillance data, we included the most recent data. Studies reporting GBS disease in infants aged 0–90 days that did not specify age at onset for the individual cases were included with the 0–89 day studies as the probability of a case on day 90 is negligible. For full details of inclusion and exclusion criteria, see Supplementary Table 2.

### Data Abstraction

We used a standardized data abstraction tool to capture information on the study design (prospective or retrospective), setting (health facility or not), use of IAP, timing of clinical disease (onset in the first 24–48 hours, EOGBS, and LOGBS), outcomes (survived or died), sample type (cerebrospinal fluid, blood, or other sterile site) and GBS serotype. For facility-based studies limited to babies born at the facility, facility live births was used as denominator. Where studies included inborn and outborn babies, a population denominator of all live births in the catchment area of the health facility was used. Data on study location were also abstracted including country and town. These data were imported into Stata version 14 software.

### Analysis

We used random-effects meta-analyses to estimate overall infant disease incidence, EOGBS and LOGBS incidence, the EOGBS to LOGBS incidence ratio, and CFRs using the DerSimonian and Laird method [21]. In addition to worldwide estimates, estimates by United Nations regions and/or subregions were obtained when sufficient data were available.

To assess bias, we performed the following sensitivity analyses:

1. Invasive disease:
  a. Infant invasive disease limited to facility-based studies where denominator was facility births.
  b. EOGBS estimates limited to studies including data for days 0–6 after birth.
  c. LOGBS estimates limited to studies including data for days 7–89 after birth.
  d. Late-onset neonatal incidence limited to studies with data for days 7–27 after birth.
2. The ratio of early-onset disease to late-onset disease, including only studies considered to be less subject to case finding bias resulting from low access to care, nonsystematic sampling, or suboptimal laboratory detection methods [22–25] as considered by the expert advisory group.

## RESULTS

### Literature Search and Study Selection

**Figure 2.**
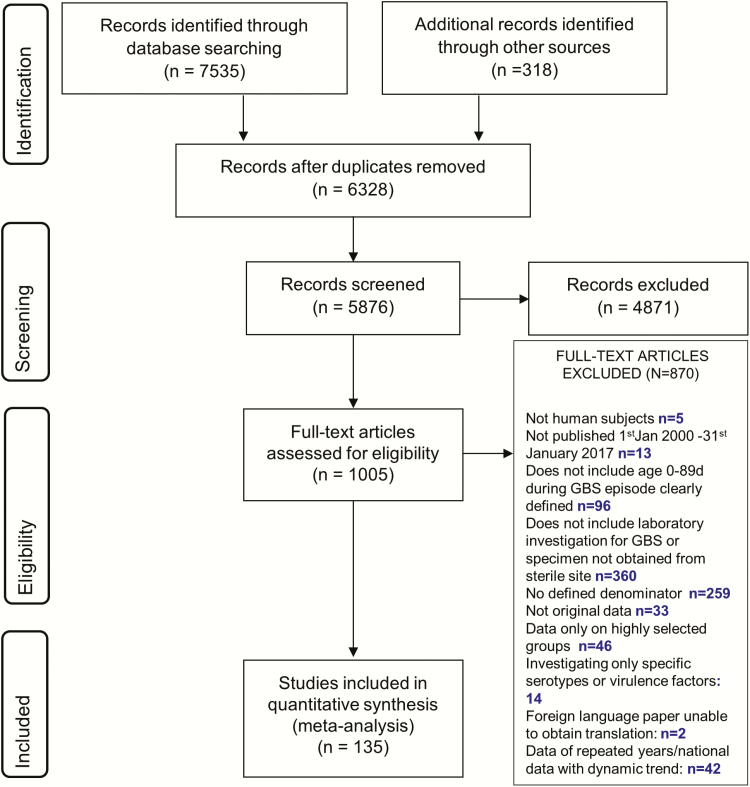
Search strategy and process of study selection. Abbreviation: GBS, group B *Streptococcus*.

We identified 7535 articles for consideration from database searches, 318 additional records from expert groups in neonatal care and reference lists, and 7 datasets from an investigator group [22, 23, 26] (Araujo da Silva et al. unpublished, Dhaded et al. unpublished, Saha et al. unpublished, Sigaúque et al. unpublished). One hundred thirty-five articles (reporting data from 57 countries) met our inclusion criteria (search strategy of study selection in Figure 2). Of these, 90 reported incidence [22–96], (EOGBS: 74 studies; LOGBS: 33 studies), 64 reported CFR [22–27, 30, 34, 37, 38, 41–43, 46, 49–57, 59, 61, 63, 68, 70, 71, 73–75, 78–81, 84, 89, 90, 92, 95–111], and 45 reported serotype data [22–25, 31, 42, 47, 50, 55, 61, 63, 66, 70, 73, 78, 81, 84, 112–138]. (The full list of articles included in this review is available in Supplementary Table 3.) Articles excluded because more recent data from the same population were available are shown in Supplementary Table 4 and Supplementary Figure 1. Compared to the previously published global GBS invasive disease estimates [9], we included 61 additional studies: 34 reporting incidence, 35 CFR, and 26 serotype (Supplementary Figure 2).

### Study Characteristics

**Figure 3.**
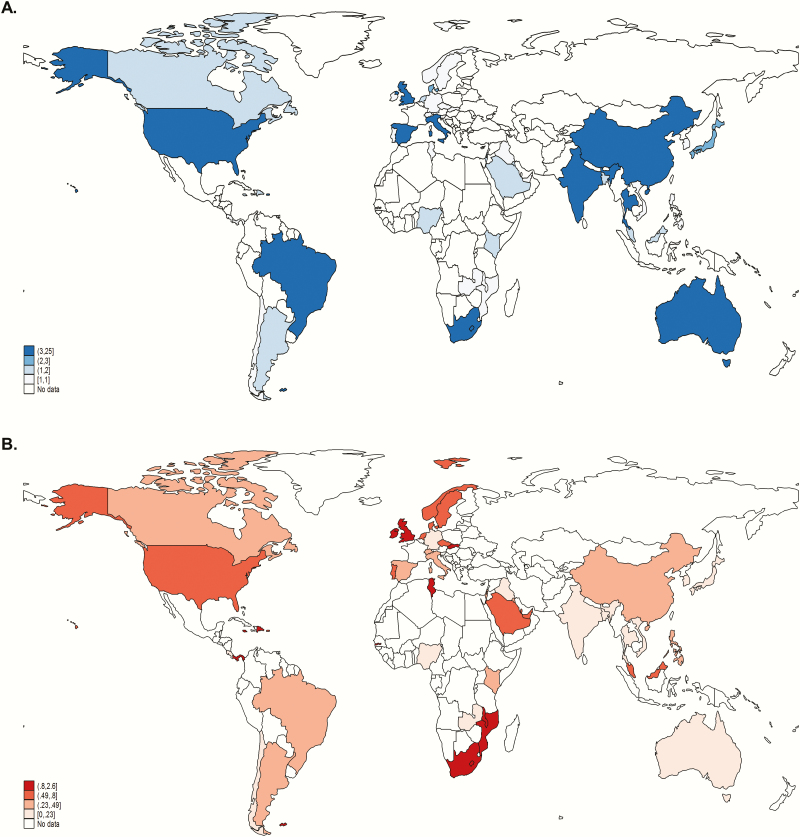
Worldwide distribution of data inputs. *A*, Map illustrating number of studies by country reporting incidence of group B streptococcal (GBS) invasive disease. *B*, Map illustrating overall incidence of GBS disease among infants by country included in the meta-analyses. Borders of countries/territories in map do not imply any political statement.

There were more data from HICs (77 studies) compared to LMICs (18 studies), of which 12 were from Africa (11 in sub-Saharan Africa, 1 in North Africa). Data inputs are illustrated in Figure 3A and 3B. Data inputs of the previous systematic review [9] are shown in Supplementary Figures 3*A* and 3*B*. Most studies (109/135) were facility based and information about IAP use was available from 116 of 135 studies (Table 1). Seventy-six studies reported any use of IAP: 27 of 76 (35.5%) were based on screening, 14 of 76 (18.4%) were based on a risk factor algorithm, and 35 of 76 (46.1%) did not specify a strategy. Of those studies reporting incidence, 58 of 90 (64.4%) reported use of any IAP; this was highest in developed countries (46/58 [79.3%]) and lowest in sub-Saharan Africa (3/11 [27.3%]) (Supplementary Figure 4). Of 74 studies that reported EOGBS, 49 (67.1%) reported IAP use and approximately one-third of articles (24/74 [32.4%], including 6 studies from LICs and LMICs) reported information about age at onset of EOGBS. Serotype was available in studies from 25 countries (developed countries, 16; Central and South America, 4; Southern and Eastern Africa, 3; Eastern Asia, 2). We were unable to abstract data on laboratory methods used, maternal risk factors, and weight or gestational age at birth of neonates.

**Table 1.** Characteristics of Included Studies Investigating Invasive Group B Streptococcal Disease in Infants

Characteristic	Total (135 Articles)	Incidence (90 Articles)	CFR (64 Articles)	Serotypes (47 Articles)
United Nations subregion				
Developed countries	58 (43.0)	32 (35.6)	28 (43.8)	32 (68.1)
Central America	2 (1.5)	2 (2.2)	1 (1.6)	1 (2.1)
Caribbean	6 (4.4)	5 (5.6)	4 (6.2)	0 (0.0)
South America	15 (11.1)	9 (10.0)	8 (12.5)	4 (8.5)
Northern Africa	1 (0.7)	1 (1.1)	0 (0.0)	0 (0.0)
Eastern Africa	5 (3.7)	4 (4.4)	4 (6.2)	2 (4.3)
Western Africa	3 (2.2)	3 (3.4)	1 (1.6)	0 (0.0)
Southern Africa	3 (2.2)	3 (2.3)	3 (4.7)	2 (4.3)
Eastern Asia	17 (12.6)	7 (7.8)	8 (12.5)	5 (10.6)
Western Asia	8 (5.9)	7 (7.8)	2 (3.1)	0 (0.0)
Southern Asia	7 (5.2)	7 (7.8)	3 (4.7)	1 (2.1)
Southeastern Asia	10 (7.4)	10 (11.1)	2 (3.1)	0 (0.0)
Study design				
Prospective	53 (39.3)	46 (51.1)	26 (40.6)	12 (25.5)
Retrospective	82 (60.7)	44 (48.9)	38 (59.3)	35 (74.5)
Population/facility-based study^a^				
Population-based	24 (18.8)	18 (20.0)	13 (20.3)	35 (76.1)
Facility based	109 (81.2)	71 (78.9)	50 (78.1)	11 (23.9)
Reporting period				
Full period (0–89 d)^b^	10 (7.4)	10 (11.1)	10 (15.6)	6 (12.7)
Full EOGBS period (0–6 d)^c^	42 (31.1)	42 (46.7)	30 (46.9)	13 (27.7)
Full LOGBS period (7–89 d)^d^	11 (8.1)	11 (12.2)	11 (17.2)	5 (10.6)
Specimen type				
Blood only	27 (20.0)	19 (21.8)	12 (18.8)	3 (6.4)
CSF only	5 (3.7)	2 (2.3)	2 (3.1)	2 (4.3)
Blood and CSF	75 (55.6)	53 (58.9)	36 (56.3)	27 (57.5)
All sterile sites	25 (18.5)	14 (15.6)	14 (21.9)	15 (31.9)
IAP				
Any IAP used	76 (65.5)	58 (69.9)	41 (70.7)	21 (43.8)
No IAP	40 (34.5)	25 (30.1)	17 (29.3)	27 (56.2)
Rural/urban				
Rural	2 (1.5)	2 (2.2)	1 (1.6)	1 (2.1)
Urban	69 (51.1)	46 (51.1)	33 (51.6)	21 (44.7)
Semirural	2 (1.5)	2 (2.2)	2 (3.1)	2 (4.3)
Mixed	30 (22.2)	22 (24.4)	15 (23.4)	11 (23.4)
Not described	32 (23.7)	18 (20.0)	13 (20.3)	12 (25.5)

Data are presented as No. (%).

Abbreviations: CFR, case fatality risk; CSF, cerebrospinal fluid; EOGBS, early-onset group B *Streptococcus*; IAP, intrapartum antibiotic prophylaxis; LOGBS, late-onset group B *Streptococcus*.

^a^Two missing values for population/facility-based and 19 missing values for IAP use.

^b^Studies reporting incidence among infants for the whole period aged 0-89 days among all studies.

^c^Studies reporting EOGBS cases among infants for the whole period aged (0-6 days) among studies reporting EOGBS in each category.

^d^Studies reporting LOGBS cases among infants for the whole period (7–89 days) among studies reporting EOGBS in each category.

### Incidence Risk of Group B *Streptococcus* Disease

**Figure 4.**
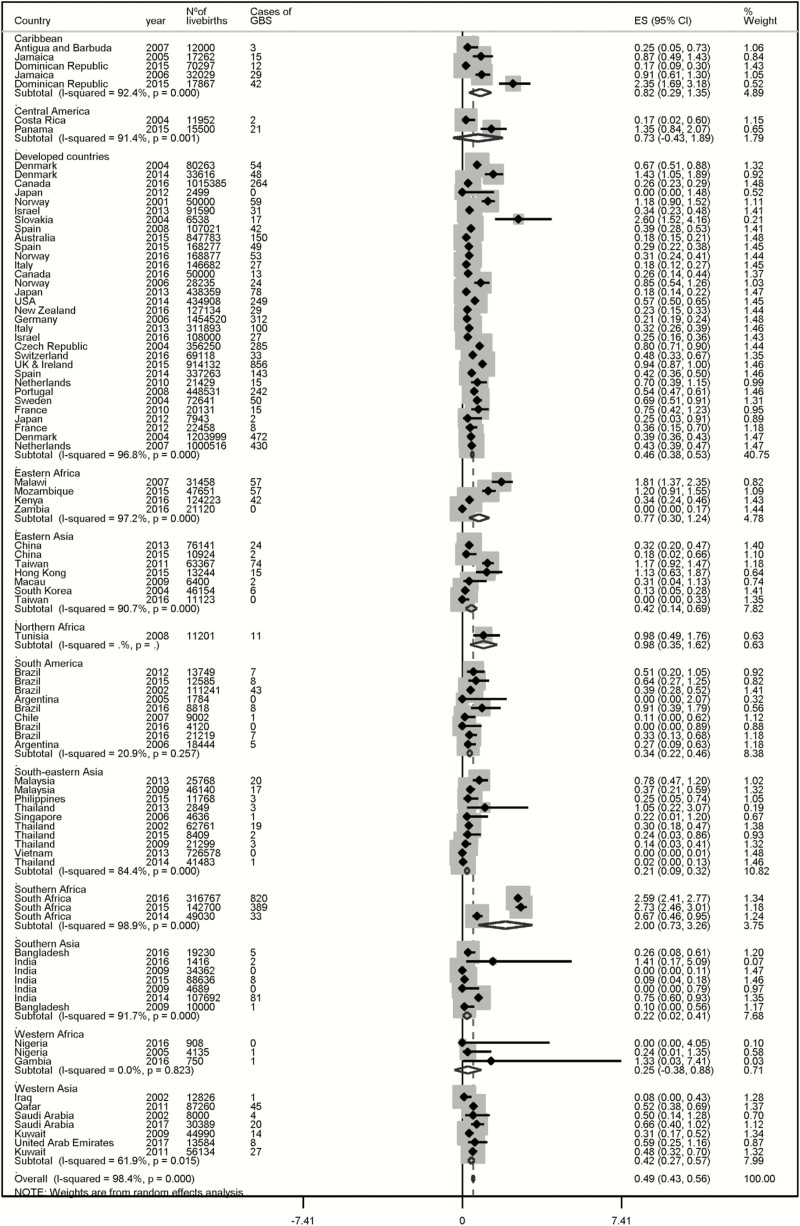
Pooled estimated incidence risk per 1000 live births of overall infant invasive group B streptococcal disease. Abbreviations: CI, confidence interval; ES, effect size; GBS, group B *Streptococcus*.

There were 6199 infants with invasive GBS disease among 13300000 live births in 53 countries. The incidence risk (per 1000 live births) for infant GBS disease was 0.49 (95% CI, .43–.56) overall, being 1.12 in Africa, 0.49 in Latin America and the Caribbean, 0.46 in developed countries, and the lowest in Asia, 0.30. Incidence was highest in Southern Africa (2.00 [95% CI, .74–3.26]) and lowest in Southeast Asia (0.21 [95% CI, .09–.32]; meta-analysis in Figure 4). There were 3664 cases of EOGBS from 9866793 live births. Incidence risk (per 1000 live births) of EOGBS worldwide was 0.41 (95% CI, .36–.47) and ranged from 0.32 (95% CI, .22–.41) in Asia to 0.71 (95% CI, .24–1.18) in Africa. The Caribbean had the highest incidence risk of EOGBS (1.47), followed by Southern Africa (1.07) and South Asia the lowest (0.20) (Supplementary Figure 5). Among EOGBS cases, 68% (95% CI, 57%–79%) developed symptoms in the 24 hours after birth, being higher in HIC (74% [95% CI, 58%–89%]) compared with LICs (31% [95% CI, –20% and 82%]); meta-analysis included as Supplementary Figure 6). There were 2003 cases of LOGBS among 8975899 live births. Incidence risk of LOGBS worldwide was 0.26 (95% CI, .21–.30), ranging from 0.04 (95% CI, –.02 to .09) in Asia to 0.65 (95% CI, .25–1.05) in Africa. Southern Africa had the highest incidence risk of LOGBS (0.93), and South America, Western Africa, and Southeastern Asia had the lowest (0.0, 0.0, and 0.03, respectively, based on the single study captured from each of these regions; Supplementary Figure 7).

### Case Fatality Risk

There were 570 deaths among 6501 infant cases. The overall CFR was 8.4% (95% CI, 6.6%–10.2%). CFR in Africa (18.9% [95% CI, 13.7%–24.0%]) was 4 times higher than in developed countries (4.7% [95% CI, 3.3%–6.1%]) (meta-analysis in Supplementary Figure 8). EOGBS CFR was 10.0% (95% CI, 7.0%–12.0%) ranging from 5.0% (95% CI, 4.0%–7.0%) in developed countries to 27.0% (95% CI, 17.0%–37.0%) in Africa. LOGBS CFR was 7.0% (95% CI, 4.0%–9.0%) and, consistently with overall and EOGBS CFR, was lowest in developed countries (4.0% [95% CI, 3.0%–6.0%]) and highest in Africa (12.0% [95% CI, 5.0%–19.0%]) (meta-analysis in Supplementary Figures 9 and 10, respectively).

### Serotype Distribution

**Figure 5.**
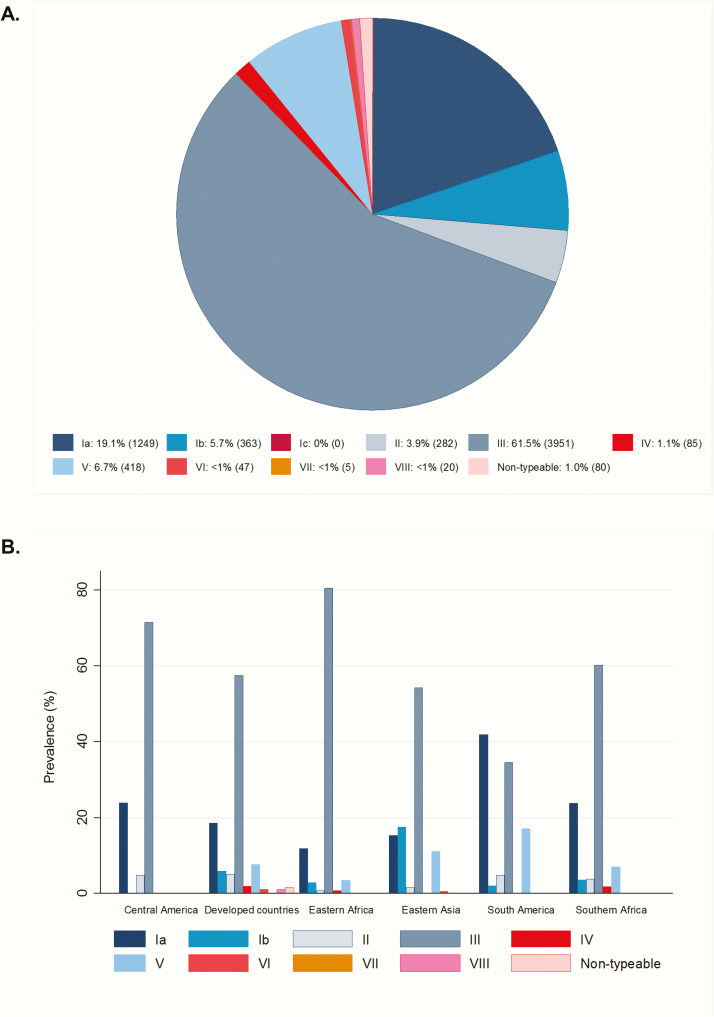
Global distribution of group B *Streptococcus* (GBS) serotypes in invasive disease in young infants (N = 6500 isolates). *A*, Prevalence of GBS serotypes presented as percentage (number of cases). *B*, Distribution of GBS serotypes by region. Serotypes included in a pentavalent vaccine are shown in blue and those not included are shown in red.

A total of 6500 bacterial isolates were included in the meta-analysis of serotype prevalence (data inputs are illustrated in Supplementary Figure 11). Five serotypes (Ia, Ib, II, III, and V) accounted for 97% of invasive isolates in all regions with serotype data (Figure 5). Serotype III was the most prevalent serotype across the United Nations subregions, although it was lower in South America (34%) compared with other subregions. Nearly half (47%) of EOGBS cases and 73.0% of LOGBS cases were caused by serotype III. Serotype Ia, Ib, and V were frequently isolated in EOGBS (22.8%, 8.0%, and 10.6%, respectively) and LOGBS (14.2%, 5.3%, and 4.0%) (Supplementary Figure 12).

### Early-Onset to Late-Onset Group B Streptococcus Disease Ratio

The overall ratio of EOGBS to LOGBS disease was 1.72 (95% CI, 1.35–2.21). The highest ratio was in Asia (5.99 [95% CI, 2.40–14.92]) and lowest in Africa (1.02 [95% CI, .82–1.28]). The ratio in developed countries was similar to the overall ratio, at 1.82 (95% CI, 1.29–2.57) (meta-analysis as Supplementary Figure 13).

### Clinical Syndrome (Sepsis or Meningitis)

Twenty-three percent of all GBS invasive cases (95% CI, 14%–32%) were meningitis. Among EOGBS cases, 78% (95% CI, 67%–88%) had sepsis and 16% (95% CI, 8%–25%) had meningitis (meta-analysis of meningitis cases among EOGBS cases in Supplementary Figure 14). The meningitis/sepsis ratio was 0.18 (95% CI, .13–.25). Among LOGBS cases, there was a lower percentage of sepsis; 53% (95% CI, 43%–62%) had sepsis and 43% (95% CI, 34%–51%) had meningitis (meta-analysis of meningitis cases among LOGBS cases in Supplementary Figure 15). The meningitis:sepsis ratio was 0.78 (95% CI, .55–1.10).

### Sensitivity Analyses to Assess Bias

Among facility-based studies with facility births denominator (n = 71), the incidence among infants 0–89 days of age was slightly higher than the main analysis (0.53 [95% CI, .44–.61]). The highest incidence was in Southern Africa (2.00 [95% CI, .73–3.26]) and the lowest in Southeastern Asia (0.21 [95% CI, .09–.32]) (Supplementary Figure 16). EOGBS incidence was 0.43 (95% CI, .35–.50) per 1000 live births (Supplementary Figure 17) and LOGBS incidence was 0.31 (95% CI, .24–.38) per 1000 live births (Supplementary Figure 18).

When we limited estimates of EOGBS to studies with reported data for days 0–6 of life (42/74 studies with EOGBS data reported), EOGBS incidence (0.42 [95% CI, .35–.49]) was similar to the main analysis (meta-analyses in Supplementary Figure 19).

When we limited LOGBS estimates to studies with complete data for days 7–89 after birth (11/33 studies), the incidence estimate was 0.40 per 1000 live births (95% CI, .27–.53), higher than the main analysis (meta-analysis in Supplementary Figure 20). Including only studies with data for days 7–27 after birth (3/32 studies), the incidence was 0.16 per 1000 live births (95% CI, .08–.24) (meta-analysis in Supplementary Figure 21).

When we limited estimates of the EOGBS to LOGBS ratio to studies considered to be less subject to bias (14 studies [22–25, 31, 42, 52, 56, 62, 63, 73, 81]), the estimated ratio was 1.11 (95% CI, .96–1.30) and, unlike the main analysis, was similar across geographic regions (meta-analysis in Supplementary Figure 13).

## DISCUSSION

Our comprehensive review and meta-analyses represent an important update to the previous global invasive infant GBS disease burden estimates [9], and most notably include new data from LMICs (18 new studies from 10 LICs and LMICs). Infant invasive GBS disease incidence and case fatality is high in every world region, yet likely considerably underestimated in settings with limited access to care and diagnostics as <10% of neonates with suspected serious infection have a positive blood culture [139, 140]. The overall estimated incidence of infant GBS disease, 0.49 per 1000 live births, is slightly lower than the previous estimate of 0.53 per 1000 live births (95% CI, .41–.62) [9]. While fewer studies in this review reported IAP use compared to the previous review (66% vs 77.0%), more weight (>50%) was applied to the data from Europe and the Americas where IAP is in use. The reduction in overall incidence is likely driven by lower incidence of invasive infant GBS disease in the Americas (0.43/1000 live births here vs 0.67/1000 live births in the previous review), and Europe (0.53 vs 0.57/1000 live births). This difference, especially in the United States where infant GBS rates declined notably during the study period, reflects the use of more recent data in our analysis.

Similarly, the incidence of invasive infant GBS disease in Africa (1.12/1000 live births) was also slightly lower than previously reported, although >2 times higher than in developed countries (0.46/1000 live births). This is the result of broader incidence data from Africa, including large studies in South Africa [24, 42, 96] Mozambique ”(Sigaúque et al, Unpublished data)”, and Gambia [68] reporting a high incidence of invasive GBS disease, in contrast to studies in Nigeria [28, 77] and Zambia [62] reporting a very low incidence. Our point estimate for EOGBS incidence for Africa was higher compared to that reported in the most recent worldwide review [9], although LOGBS incidence for the same region was similar in both reviews [9]. There are many possible reasons for the increase in EOGBS incidence in Africa, which could be due to true emergence, increases in comorbidities such as HIV [42], or improved data collection to detect early disease. This high incidence is important in terms of total burden, as CFRs in Africa were also 4 times higher than in developed countries (18.9% and 4.7%, respectively); thus the greatest burden of cases, and deaths, is in Africa. However, our data are limited to few African studies mostly in Southern and Eastern Africa.

There are other important regional differences. The incidence of infant GBS disease was strikingly low in Asia at 0.31 per 1000 live births, with the lowest incidence in Southeast Asia (0.21/1000 live births). This may reflect a true regional difference, which could be related to differences in lower overall prevalence of maternal colonization and/or lower prevalence of serotype III [13], which is more commonly associated with the most virulent clone, clonal complex 17. Some of the difference may also be due to incomplete case ascertainment, being in Asia more challenging as they have more home births than Africa. For the earliest-onset cases (<24 hours of birth), differences in access to care and rapid and high case fatality can reduce case ascertainment. Cerebrospinal fluid sampling is infrequently performed in many parts of this region and that would reduce the apparent incidence of LOGBS disease, which is more frequently associated with meningitis. However, the lack of late-onset cases in this region does not fully align with those reasons and also suggests there may be more at play, potentially related to strain differences, level of natural acquired protective maternal antibody, or other host, environmental, or behavioral factors that may affect disease burden.

Difficulties in case ascertainment in LICs likely contribute to the higher incidences observed when the analysis was limited to facility-based studies, particularly in Africa. Studies in contexts where access to care, particularly for home deliveries, is difficult are likely to underestimate EOGBS disease incidence, due to the preponderance of cases with onset on day zero, which can be as high as 90% in studies with high-quality ascertainment but was 68% among the studies we included where this information could be extracted. Late-onset disease is likely underestimated too, due to studies that did not capture cases for the full 7–89 days; the sensitivity analysis showed the incidence of late onset disease to be almost twice as high (0.40 vs 0.26) when only studies with data for days 7–89 were included. This may result in an underestimation of the burden of GBS meningitis in particular, a significant concern given the morbidity associated with this condition.

Differences in the early- to late-onset disease ratios in different regions in the main analysis may also reflect (and reveal) biases in the data. Asia had the highest ratio of EOGBS disease to LOGBS disease, with the lowest in Africa (5.99 vs 1.02). It is possible that Asia has less LOGBS disease, consistent with the lower prevalence of maternal colonization with serotype III, a serotype commonly associated with LOGBS disease. Interestingly, when limiting the EOGBS:LOGBS ratio to high-quality studies, the estimate was similar across regions and lower than the overall EOGBS:LOGBS ratio. This is likely to be influenced by those countries with widespread IAP as well as South Africa, which may have a uniquely low ratio due to the high prevalence of maternal HIV infection, which predisposes to late-onset disease. However, no study from Asia was included in this analysis.

In terms of serotypes causing invasive disease, serotype III accounted for over half of all disease-causing isolates followed by serotypes Ia, V, and II, consistent with previous work [9, 141]. Disease-causing serotypes were similar in prevalence across different regions, with some slight variations. The lowest prevalence of serotype III (where data were available) was found in South America and Southeast Asia, 2 of the regions with the lowest prevalence of serotype III among colonized pregnant women [13]. Serotype distribution was similar to that reported in the previous review [9], suggesting stability over time.

Our findings suggest GBS disease is an important cause of infant disease, despite the limitations in the data and uncertainties about the low incidence in Asia. In Africa, where the incidence is highest, the CFR is also highest, suggesting this is the region where prevention strategies are most critical to introduce. Existing preventive strategies using IAP are not usually available in low-income contexts and, with a higher number of home deliveries and late presentation to health facilities for delivery, IAP may be more difficult to implement. Maternal vaccination offers an alternative strategy, and the data we have suggest that a pentavalent conjugate vaccine (including Ia/Ib/II/III/V) would cover almost all disease-causing serotypes (96%) in young infants worldwide (Table 2).

**Table 2.** Key Findings and Implications

What’s new about this? • Most comprehensive review to date of published data, and supplemented by unpublished data, including more data especially from LICs and LMICs with total of 18 studies from 10 LICs and LMICs. • Extensive attempts to standardize inputs and to assess biases through a set of sensitivity analyses.
What was the main finding? • GBS is an important cause of invasive disease among infants worldwide, despite widespread use of intrapartum antibiotic prophylaxis in many countries in developed regions. • Highest incidence is in Africa; the lowest incidence is in Asia and not fully explained by these data.
How can the data be improved? • Lower incidence in Asia may be partially explained by lower maternal colonization rates and less-virulent serotypes, but more data are required to better understand regional differences. • Improved reporting of studies to better understand the biases in the data reported, for example, if low case ascertainment in the first 24 hours after birth, may be reducing reported GBS incidence rates, and if this occurs more frequently in some regions.
What does it mean for policy and programs? • Prevention strategies are needed in all settings and particularly in the highest-burden settings (in Africa) • Higher proportion of meningitis is among LOGBS cases, for which there are currently no preventive strategies. • A pentavalent vaccine (serotypes Ia/Ib/II/III/V) would cover the GBS serotypes causing almost all (96%) invasive disease in infants.

Abbreviations: GBS, group B *Streptococcus*; LIC, low-income context; LMIC, low- to middle-income context; LOGBS, late-onset group B *Streptococcus*.

## Supplementary Data

Supplementary materials are available at *Clinical Infectious Diseases* online. Consisting of data provided by the authors to benefit the reader, the posted materials are not copyedited and are the sole responsibility of the authors, so questions or comments should be addressed to the corresponding author.

## Supplementary Material

supplement-material

## Notes

***Author contributions.*** The concept of the estimates and the technical oversight of the series were led by J. E. L. and A. C. S. The reviews, analyses, and first draft of the manuscript were undertaken by L. M. with A. C. S. and S. S. Other specific contributions were made by M. K. L., K. M. E., J. E. L., P. T. H., S. A. M. The GBS Estimates Expert Advisory Group (C. J. B., L. B., C. C., M. G. G., M. I., K. L. D., C. E. R., S. K. S., A. S.-t. M., J. V.) contributed to the conceptual process throughout, notably on the disease schema and data inputs. The Infant GBS Invasive Disease Investigator Group (R. A., A. R. A. d. S., Q. B., J. A. B., Z. B., S. D., E. G., H. M., K. L. D., C. O. S., S. Sri., H. S., D. N. B. S. S., B. S., G. T., P. V. K.) input data for the analyses. All the authors reviewed and gave input to the manuscript.

***Acknowledgments.*** L. M. would like to thank her PhD advisor Dr Quique Bassat, who supported her work in this project during her PhD program and S. Nanduri for his kind effort to prepare and share his data. The authors also thank Claudia da Silva for administrative assistance and Alegria Perez for coordinating author signatures.

***With the Infant Invasive GBS Disease Investigator Group.*** Ramesh Agarwal (Newborn Health Knowledge Centre, India), Andre Ricardo Araujo da Silva (Universidade Federal Fluminense, Niterói, Brasil), Quique Bassat (ISGlobal, Spain; Centro de Investigação em Saúde de Manhiça, Mozambique; ICREA, Spain), James A. Berkley (Kenya Medical Research Institute), Ziyaad Dangor (Respiratory and Meningeal Pathogens Research Unit, South Africa), Sangappa Dhaded (KLE University, India), Eric Giannoni (Lausanne University Hospital and Swiss Pediatric Sepsis Study Group, Switzerland), Majeda Hammoud (Department of Pediatrics, Faculty of Medicine, Kuwait University, Kuwait), Miwako Kobayahsi (US Centers for Disease and Control Prevention), Catherine O′Sullivan (Paediatric Infectious Diseases Research Group, St George’s, University of London, UK), Hiro Sakata (Asahikawa Kosei Hospital, Japan), Santhanam Sridhar (Christian Medical College, India), Betuel Sigaúque (Centro de Investigação em Saúde de Manhiça, Mozambique), Greg Tyrrell (Alberta Health Services, Canada), Vinod Paul (Newborn Health Knowledge Centre, India).

***Financial support.*** This supplement was supported by a grant to the London School of Hygiene & Tropical Medicine from the Bill & Melinda Gates Foundation (reference number OPP1131158).

***Supplement sponsorship.*** This article appears as part of the supplement “The Burden of Group B *Streptococcus* Worldwide for Pregnant Women, Stillbirths, and Children,” sponsored by the Bill & Melinda Gates Foundation and coordinated by the London School of Hygiene & Tropical Medicine.

***Potential conflicts of interest.*** Many contributors to this series of papers have received funding for their research from foundations, especially the Bill & Melinda Gates Foundation, and several from the Wellcome Trust, Medical Research Council UK, the Thrasher Foundation, the Meningitis Research Foundation, and one individual from the US National Institutes of Health. Members of the Expert Advisory Group received reimbursement for travel expenses to attend working meetings related to this series. A. S.-t. M. works for the Bill & Melinda Gates Foundation. C. J. B. has served as a member of the Presidential Advisory Committee for Seqirus Inc and of the CureVac Inc Scientific Advisory Committee, as well as undertaken consultancy work for Pfizer Inc. C. C. has received institutional compensation from Novartis for conducting GBS studies. P. T. H. has been a consultant to Novartis and Pfizer on GBS vaccines but received no funding for these activities. M. I. has undertaken sponsored research from Pfizer on pneumococcal disease in adults and from Belpharma Eumedica (Belgium) on temocillin antimicrobial susceptibility in Enterobacteriaceae. K. L. D. has received funding by the Bill & Melinda Gates Foundation to work on research on GBS serocorrelates of protection to inform vaccine trials, and travel expenses from Pfizer to attend a meeting on an investigator-led project on GBS. S. A. M. has collaborated on GBS grants funded by GlaxoSmithKline and by Pfizer and received personal fees for being member of its advisory committee; he has also collaborated on a GBS grant funded by Minervax. All authors have submitted the ICMJE Form for Disclosure of Potential Conflicts of Interest. Conflicts that the editors consider relevant to the content of the manuscript have been disclosed.
