# Supplementary material for: Infant Group B Streptococcal Disease Incidence and Serotypes Worldwide: Systematic Review and Meta-analyses
Source: Clin Infect Dis. 2017 Nov 6;65(Suppl 2):S160–72. doi: 10.1093/cid/cix656 (PMC5850457; doi:10.1093/cid/cix656)
Supplement: supplement-material [file cix656_suppl_supplement-material.pdf]

# The burden of Group B *Streptococcus* worldwide for pregnant women, stillbirths and children

**Paper 8:** Infant Group B Streptococcal disease incidence and serotypes worldwide:  systematic review and meta-analyses

## Supplementary information

## Contents


**The burden of Group B *Streptococcus* worldwide for pregnant women, stillbirths and children** ..... 1

**Paper 8:** Infant Group B Streptococcal disease incidence and serotypes worldwide: systematic review and meta-analyses ..... 1

**Supplementary information** ..... 1

Supplementary Table S1: Search terms ..... 4

Supplementary Table S2: Inclusion and exclusion criteria ..... 5

Supplementary Table S3: Characteristics of studies included and data type extracted for invasive infant GBS disease ..... 6

Supplementary Table S4: Studies excluded (repeated data or more recent data available) ..... 10

Supplementary Figure S1: Incidence of invasive infant GBS disease in studies from United States, 2002-2017, grouped in year periods ..... 12

Supplementary Figure S2: Publication years of included studies on incidence of invasive infant GBS disease ..... 13

Supplementary Figure S3A. Map illustrating number of studies by country reporting incidence of GBS invasive disease in the previous systematic review by Edmond[1]. ..... 14

Supplementary Figure S3B. Map illustrating overall incidence of GBS disease among infants aged 0-89 days by country included in the previous meta-analysis by Edmond[1]. ..... 15

Supplementary Figure S4. Regional distribution of included studies assessing incidence of infant GBS disease, and use of intrapartum antibiotic prophylaxis, 2000-2017. * ..... 16

Supplementary Figure S5: Incidence risk of early-onset GBS disease worldwide ..... 17

Supplementary Figure S6: Percentage of early-onset GBS disease cases in the first 24 hours after birth, of all early-onset (days 0-6) cases. ..... 18

Supplementary Figure S7: Incidence risk of late-onset GBS disease worldwide by region ..... 19

Supplementary Figure S8: Case fatality risk of GBS disease in infants aged 0-89 days worldwide by region ..... 20

Supplementary Figure S9: Case fatality risk of early-onset GBS disease by region. ..... 21

Supplementary Figure S10: Case fatality risk of late-onset GBS disease by region. ..... 22

Supplementary Figure S11: Map of included studies reporting serotype data for infant GBS disease ..... 23

Supplementary Figure S12: Distribution of GBS serotypes for A) early onset GBS disease and B) late onset GBS disease ..... 24

Supplementary Figure S13A: Ratio of early to late-onset GBS disease cases among infant GBS disease cases ..... 25

Supplementary Figure S13B: Early to late-onset ratio for infant invasive GBS disease in high quality studies. ..... 26

Supplementary Figure S14: Meningitis cases among early-onset GBS cases. ..... 27

Supplementary Figure S15: Meningitis cases among late-onset GBS cases ..... 28

Supplementary Figure S16: Incidence of GBS disease among infants aged 0-89 days in facility-based studies by region. ........ 29

Supplementary Figure S17: Incidence of early-onset GBS disease among infants in facility-based studies by region ........ 30

Supplementary Figure S18: Incidence of late-onset GBS disease among infants in facility-based studies by region ........ 31

Supplementary Figure S19: Incidence of early-onset of GBS disease among infants aged 0-6 days by region* ........ 32

Supplementary Figure S20: Incidence of late-onset of GBS disease among infants aged 7-89 days by regions* ........ 33

Supplementary Figure S21: Incidence of late-onset of GBS disease among infants aged 7-27 days by country ........ 34

## Supplementary Table S1: Search terms

| |
|---|
| infant |
| outcome |
| Death |
| Mortality |
| Case AND Fatality AND rate |
| Death [MeSH Terms] |
| Mortality [MeSH Terms] |
| Case fatality rate [MeSH Terms] |
| ***AND*** |
| Streptococcus |
| Streptococcal |
| Streptococci AND (Group AND B) or agalactiae |
| Streptococcus agalactiae [MeSH Terms] |
| ***AND*** |
| Streptococcus serotype |
| Streptococcal serotype |
| Streptococcus agalactiae  erotype[MeSH Terms] |

## Supplementary Table S2: Inclusion and exclusion criteria

| | Inclusion criteria | Exclusion criteria |
|---|---|---|
| **Population** | Invasive GBS disease in infants aged 0-89 days at onset of infection | Studies containing only information on very high-risk groups |
| **Laboratory** | GBS confirmed by blood / CSF culture. | |
| **Search** | No language restrictions | Foreign language papers where it was not possible to obtain English translation |
| **Article type** | Study reporting more recent data from country with dynamic trend | Case reports, case series, reviews. Studies from the same country reporting repeated years or with dynamic trend over time |

Supplementary Table S3: Characteristics of studies included and data type extracted for invasive infant GBS disease

| Author | Country | Year | Year data collection | Study type | Incidence | Case fatality risk | Serotype | IAP use | Included in previous review[1] |
|---|---|---|---|---|---|---|---|---|---|
| Andersen[2] | Denmark | 2004 | 1992-2001 | Community-based | Yes | | | Yes | Yes |
| Bell[3] | Jamaica | 2005 | 1995-2000 | Facility-based | Yes | | | No | Yes |
| Carbonell-Estrany[4] | Spain | 2008 | 2004-2005 | Facility-based | Yes | Yes | | Yes | Yes |
| Chang[5] | Taiwan | 2003 | 1986-2001 | Facility-based | | Yes | Yes | Yes | Yes |
| Davies[6] | Canada | 2001 | 1993-1999 | Community-based | | | Yes | Yes | Yes |
| Ekelund[7] | Denmark | 2004 | 1984-2002 | Facility-based | Yes | | Yes | Yes | Yes |
| Fluegge[8] | Germany | 2006 | 2001-2003 | Community-based | Yes | Yes | | Yes | Yes |
| Hasseltvedt[9] | Norway | 2001 | 2000 | Community-based | Yes | Yes | | Yes | Yes |
| Janek[10] | Slovakia | 2004 | 2000-2003 | Facility-based | Yes | | | Yes | Yes |
| Jiang[11] | Taiwan | 2004 | 1992-2001 | Facility-based | | Yes | | No | Yes |
| Kim[12] | South Korea | 2004 | 1999-2001 | Community-based | Yes | | | No | Yes |
| Neto[13] | Portugal | 2008 | 2001-2005 | Community-based | Yes | Yes | | Yes | Yes |
| Niduvaje[14] | Singapore | 2006 | 1999-2000 | Facility-based | Yes | | | Yes | Yes |
| Ben Hamida[15] | Tunisia | 2008 | 2001-2003 | Facility-based | Yes | | | No | Yes |
| Trijbels-Smeulders[16] | Netherlands | 2007 | 1997-2001 | Facility-based | Yes | Yes | | Yes | Yes |
| Trotman[17] | Jamaica | 2006 | 1991-2000 | Facility-based | Yes | Yes | | No | Yes |
| Yossuck[18] | Thailand | 2002 | 1996-2001 | Facility-based | Yes | | | No | Yes |
| Hoshina[19] | Japan | 2002 | 1983-1997 | Facility-based | | | Yes | No | Yes |
| Gray[20] | Malawi | 2007 | 2004-2005 | Facility-based | Yes | Yes | | No | Yes |
| Figueira-Coelho[21] | Portugal | 2004 | 1999-2000 | Facility-based | | | Yes | Yes | Yes |
| Hajdu[22] | Norway | 2006 | 2006 | UNK | Yes | Yes | | Yes | Yes |
| Vaciloto[23] | Brazil | 2002 | 1991-2000 | Facility-based | Yes | | | No | Yes |
| Strakova[24] | Czech Republic | 2004 | 2001-2002 | Facility-based | Yes | Yes | Yes | No | Yes |
| Park[25] | South Korea | 2010 | 1996-2005 | Facility-based | | Yes | | No | Yes |
| Matsubara[26] | Japan | 2009 | 1998-2007 | Facility-based | | | Yes | No | Yes |
| Darmstadt[27] | Bangladesh | 2009 | 2004-2006 | Community-based | Yes | | | Yes | Yes |
| Al-Zwaini[28] | Iraq | 2002 | 2000-2001 | Facility-based | Yes | | | No | Yes |
| Sundaram[29] | India | 2009 | 1995-2006 | Facility-based | Yes | | | No | Yes |
| Martin[30] | Antigua and Barbuda | 2007 | 1994-2002 | Facility-based | Yes | | | Yes | Yes |
| Cho[31] | South Korea | 2010 | 1996-2005 | Facility-based | | Yes | | No | Yes |
| Van den Hoogen[32] | Netherlands | 2010 | 1978-2006 | Facility-based | Yes | | | Yes | Yes |
| Kuhn[33] | France | 2010 | 2003-2004 | Community-based | Yes | | Yes | Yes | Yes |
| Milledge[34] | Malawi | 2005 | 1996-2001 | Facility-based | | Yes | | No | Yes |
| Zhao[35] | Australia | 2008 | 1994-2005 | Facility-based | | | Yes | Yes | Yes |
| Martins[36] | Portugal | 2007 | 2000-2004 | UNK | | | Yes | Yes | Yes |
| Trijbels-Smeulders[37] | Netherlands | 2006 | 1997-1999 | Facility-based | | | Yes | Yes | Yes |
| Fluegge[38] | Germany | 2005 | 2001-2003 | Facility-based | | | Yes | Yes | Yes |
| Persson[39] | Sweden | 2004 | 1998-2001 | Facility-based | Yes | Yes | Yes | Yes | Yes |
| Davies[40] | Canada | 2004 | 1995-1999 | Community-based | | | Yes | Yes | Yes |

| | | | | | | | | | |
|---|---|---|---|---|---|---|---|---|---|
| Bidet[41] | France | 2003 | 1990-2002 | Facility-based | | | Yes | No | Yes |
| Lopardo[42] | Argentina | 2003 | 1998-1999 | Facility-based | | | Yes | No | Yes |
| El-Said[43] | Saudi Arabia | 2002 | 1998-2000 | Facility-based | Yes | | | No | Yes |
| Ojukwu[44] | Nigeria | 2005 | 2002-2003 | Facility-based | Yes | | | No | Yes |
| Tiskumara[45] | India | 2009 | 2008-2009 | Facility-based | Yes | | | Yes | Yes |
| Tiskumara[45] | Kuwait | 2009 | 2006-2009 | Facility-based | Yes | | | Yes | Yes |
| Tiskumara[45] | Macau | 2009 | 2006-2008 | Facility-based | Yes | | | Yes | Yes |
| Tiskumara[45] | Malaysia | 2009 | 2006-2009 | Facility-based | Yes | | | Yes | Yes |
| Tiskumara[45] | Thailand | 2009 | 2007-2009 | Facility-based | Yes | | | Yes | Yes |
| Abdelmaaboud[46] | Qatar | 2011 | 2003-2009 | Facility-based | Yes | Yes | Yes | Yes | No |
| Al-Taiar[47] | China | 2013 | 2006-2009 | Facility-based | Yes | | | UNK | No |
| Al-Taiar[47] | Malasya | 2013 | 2006-2009 | Facility-based | Yes | | | UNK | No |
| Al-Taiar[47] | Thailand | 2013 | 2006-2009 | Facility-based | Yes | | | UNK | No |
| Al-Taiar[47] | Kuwait | 2011 | 2005-2009 | Facility-based | Yes | Yes | | Yes | No |
| Bekker[48] | Netherlands | 2014 | 1987-2011 | Facility-based | | | Yes | Yes | No |
| Berardi[49] | Italy | 2013 | 2003-2010 | Community-based | Yes | Yes | | Yes | No |
| Bromiker[50] | Israel | 2013 | 1997-2007 | Facility-based | Yes | | | Yes | No |
| Chang[51] | Japan | 2014 | 2007-2012 | Community-based | | | Yes | Yes | No |
| Cutland[52] | South Africa | 2015 | 2004-2008 | Facility-based | Yes | Yes | | Yes | No |
| Cantoni[53] | Italy | 2013 | 2004-2006 | Community-based | | Yes | | Yes | No |
| Didier[54] | France | 2012 | 2007 | Community-based | Yes | Yes | | Yes | No |
| Evangelista[55] | Brazil | 2015 | 2012-2013 | Facility-based | Yes | Yes | | No | No |
| Fiolo[56] | Brazil | 2012 | 2007-2011 | Facility-based | Yes | Yes | Yes | Yes | No |
| Giannoni[57] | Switzerland | 2016 | 2011-2015 | Facility-based | Yes | Yes | | Yes | No |
| Giménez[58] | Spain | 2015 | 2004-2010 | Facility-based | Yes | Yes | Yes | Yes | No |
| Hashavya[59] | Israel | 2011 | 2005-2009 | Facility-based | | Yes | | Yes | No |
| Juncosa-Morros[60] | Spain | 2014 | 1996-2010 | Facility-based | Yes | Yes | | Yes | No |
| Kruse[61] | Vietnam | 2013 | 2009-2010 | Facility-based | Yes | | | No | No |
| Liu[62] | China | 2015 | 2013-2014 | Facility-based | Yes | | Yes | No | No |
| Matsubara[63] | Japan | 2013 | 2004-2010 | Facility-based | Yes | Yes | Yes | Yes | No |
| Miyata[64] | Japan | 2012 | 2002-2009 | Facility-based | Yes | Yes | | Yes | No |
| Oladottir[65] | Iceland | 2011 | 1975-2006 | Facility-based | | | Yes | UNK | No |
| Petersen[66] | Denmark | 2014 | 2002-2010 | Facility-based | Yes | | | Yes | No |
| Sakata[67] | Japan | 2014 | 2010-2012 | Facility-based | | Yes | | UNK | No |
| Sridhar[68] | India | 2014 | 1998-2010 | Facility-based | Yes | | | Yes | No |
| Yu[69] | Taiwan | 2011 | 2001-2005 | Facility-based | Yes | Yes | | Yes | No |
| Thatrimontrichai[70] | Thailand | 2014 | 1995-2010 | Facility-based | Yes | | | UNK | No |
| Ko Danny[71] | Australia | 2015 | 2005-2008 | Community-based | Yes | Yes | Yes | Yes | No |
| Morozumi[72] | Japan | 2014 | 2006-2011 | Community-based | | | Yes | Yes | No |
| Almeida[73] | France | 2015 | 2008-2012 | Facility-based | | | Yes | No | No |
| Brzychczy-Wloch[74] | Poland | 2014 | 2006-2010 | Facility-based | | | Yes | UNK | No |
| Fluegge[75] | Germany | 2011 | 2001-2003 | Facility-based | Yes | | Yes | UNK | No |
| Imperi[76] | Italy | 2011 | 2005-2008 | Facility-based | Yes | | Yes | UNK | No |
| Joubrel[77] | France | 2015 | 2007-2012 | Facility-based | Yes | | | Yes | No |
| Six[78] | France | 2016 | 2006-2013 | Facility-based | | | Yes | Yes | No |
| Souza[79] | Brazil | 2013 | 2008-2010 | Facility-based | | | Yes | UNK | No |

| | | | | | | | | | |
|---|---|---|---|---|---|---|---|---|---|
| Teatero[80] | Canada | 2014 | 2009-2012 | Community-based | | | Yes | Yes | No |
| Yoon[81] | Korea | 2015 | 1995-2004 | Facility-based | | | Yes | No | No |
| Wang[82] | China | 2015 | 2008-2013 | Facility-based | | | Yes | UNK | No |
| Sakata[83] | Japan | 2012 | 2009-2011 | Facility-based | Yes | | | Yes | No |
| Rivera[84] | Panama | 2015 | UNK | Facility-based | Yes | Yes | Yes | Yes | No |
| Villanueva-Uy[85] | Thailand | 2015 | UNK | Facility-based | Yes | Yes | | UNK | No |
| Larcher[86] | Argentina | 2005 | 2001-2002 | Facility-based | Yes | | | Yes | No |
| Sigauque[87] | Mozambique | 2015 | 2001-2015 | Facility-based | Yes | Yes | Yes | No | No |
| Vinod[88] | India | 2016 | 2011-2015 | Facility-based | Yes | Yes | | Yes | No |
| Le Doare[89] | Gambia | 2016 | 2014 | Facility-based | Yes | Yes | | No | No |
| Saha[90] | Bangladesh | 2016 | 2012-2013 | Facility-based | Yes | Yes | | No | No |
| Araujo da Silva[91] | Brazil | 2016 | 2015-2016 | Facility-based | Yes | | | Yes | No |
| Dhaded[92] | India | 2016 | 2014-2015 | Facility-based | Yes | Yes | | Yes | No |
| Dangor[93] | South Africa | 2016 | 2005-2014 | Facility-based | Yes | Yes | Yes | Yes | No |
| Rivera[84] | Dominican Republic | 2015 | UNK | Facility-based | Yes | Yes | | Yes | No |
| Rivera[84] | Hong Kong | 2015 | UNK | Facility-based | Yes | Yes | | Yes | No |
| Rivera[84] | Dominican Republic | 2015 | UNK | Facility-based | Yes | Yes | | Yes | No |
| Villanueva-Uy[85] | Philippines | 2015 | UNK | Facility-based | Yes | Yes | | UNK | No |
| CDC[94] | United States | 2014 | 2014 | Community-based | Yes | Yes | Yes | Yes | No |
| O´Sullivan[95] | United Kingdom | 2015 | 2014-2015 | Community-based | Yes | Yes | Yes | Yes | No |
| Seale [96] | Kenya | 2016 | 1998-2013 | Community-based | Yes | Yes | Yes | No | No |
| Alhhazmi[97] | Canada | 2016 | 2003-2013 | Community-based | Yes | | Yes | Yes | No |
| Barbosa[98] | Brazil | 2016 | 2008-2011 | Facility-based | Yes | Yes | | No | No |
| Bartlett[99] | Australia | 2017 | 2000-2015 | Facility-based | | Yes | | Yes | No |
| Berardi[100] | Italy | 2016 | 2009-2012 | Community-based | Yes | Yes | | Yes | No |
| Bulkowstein[101] | Israel | 2016 | 2007-2013 | Community-based | Yes | | | No | No |
| Campisi[102] | China | 2016 | 2013-2014 | Facility-based | | | Yes | UNK | No |
| Darlow[103] | Australia | 2016 | 2009-2011 | Community-based | Yes | Yes | | Yes | No |
| Fjalstad[104] | Norway | 2016 | 2009-2011 | Community-based | Yes | Yes | | UNK | No |
| Freitas[105] | Brazil | 2016 | 2012-2015 | Facility-based | Yes | Yes | | Yes | No |
| Ip[106] | Hong Kong | 2016 | 1993-2012 | Facility-based | | | Yes | UNK | No |
| Hammoud[107] | Kuwait | 2017 | 2013-2015 | Facility-based | Yes | | | Yes | No |
| Hammoud[107] | United Arab Emirates | 2017 | 2013-2015 | Facility-based | Yes | | | Yes | No |
| Hammoud[107] | Saudi Arabia | 2017 | 2013-2015 | Facility-based | Yes | | | Yes | No |
| Kang[108] | South Korea | 2017 | 1995-2015 | Facility-based | | Yes | | No | No |
| Li YP[109] | Taiwan | 2016 | 2006-2013 | Facility-based | Yes | Yes | | Yes | No |
| Lomuto[110] | Argentina | 2006 | 2002-2005 | Facility-based | Yes | Yes | | Yes | No |
| Mendoza[111] | Colombia | 2013 | 2005-2012 | Facility-based | | Yes | | UNK | No |
| Martinez[112] | Chile | 2004 | 1998-2002 | Facility-based | | | Yes | UNK | No |
| Poliquin[113] | Canada | 2016 | 2008-2013 | Facility-based | Yes | Yes | | Yes | No |
| Reinheimer[114] | Germany | 2016 | 2010-2016 | Facility-based | | Yes | | Yes | No |
| Zeng[115] | China | 2016 | 2012-2014 | Facility-based | | Yes | | UNK | No |
| Tapia[116] | Chile | 2007 | 2001-2004 | Facility-based | Yes | | | Yes | No |
| Kabwe[117] | Zambia | 2016 | 2013-2014 | Facility-based | Yes | | | No | No |
| Akindolire[118] | Nigeria | 2016 | 2104 | Facility-based | Yes | | | No | No |

| | | | | | | | | |
|---|---|---|---|---|---|---|---|---|
| Ovalle[119] | Chile | 2002 | 1990-2001 | Facility-based | | Yes | Yes | No |
| Delgado-Picado[120] | Costa Rica | 2004 | 2002-2004 | Facility-based | Yes | | No | No |
| Diaz Alvarez[121] | Cuba | 2008 | 1992-2007 | Facility-based | | Yes | No | No |
| Costa[122] | Brazil | 2010 | 2003-2006 | Facility-based | | Yes | Yes | No |
| Frigarti[123] | South Africa | 2014 | 2010-2011 | Facility-based | Yes | Yes | Yes | No |

CFR: case fatality risk. IAP: intrapartum antibiotic prophylaxis. UNK: unknown (information not available).

Supplementary Table S4: Studies excluded (repeated data or more recent data available)

| Author | Country | Year | Year data collection | Study type |
|---|---|---|---|---|
| Angstetra[124] | Australia | 2007 | 1994-2006 | Facility-based |
| Ireland[125] | Australia | 2014 | 2002-2011 | Facility-based |
| Daley[126] | Australia & NewZealand | 2004 | 1992-2001 | Facility-based |
| May[127] | Australia & NewZealand | 2005 | 1992-2002 | Facility-based |
| Meehan[128] | Ireland | 2014 | 2007-2011 | Facility-based |
| Berardi[129] | Italy | 2007 | 2003-2005 | Community- based |
| Berardi[130] | Italy | 2011 | 2003-2010 | Community- based |
| Berardi [131] | Italy | 2013 | 2003-2011 | Community- based |
| Berkley[132] | Kenya | 2005 | 1998-2002 | Facility-based |
| Sigauque [133] | Mozambique | 2009 | 2001-2006 | Facility-based |
| Eastwood[134] | Northern Ireland | 2015 | 2008-2010 | Facility-based |
| Madhi[135] | South Africa | 2003 | 1997-1999 | Facility-based |
| Cutland [136] | South Africa | 2009 | 2004-2007 | Facility-based |
| Cutland [137] | South Africa | 2012 | 2004-2007 | Unknown |
| Dangor[138] | South Africa | 2015 | 2012-2014 | Facility-based |
| Schrag[139] | South Africa | 2012 | 2004-2007 | Facility-based |
| Andreu[140] | Spain | 2003 | 1994-2001 | Facility-based |
| Lopez Sastre[141] | Spain | 2005 | 2000-2001 | Facility-based |
| Martins[142] | Spain | 2011 | 1994-2009 | Facility-based |
| Vergnano[143] | United Kingdom | 2010 | 2006-2008 | Facility-based |
| Lamagni[144] | United Kingdom | 2013 | 1991-2010 | Facility-based |
| Weisner[145] | United Kingdom | 2004 | 2000-2001 | Community- based |
| Meehan[146] | United Kingdom | 2015 | 2011-2013 | Facility-based |
| Oddie[147] | United Kingdom | 2002 | 1998-2000 | Facility-based |
| Okike[148] | United Kingdom | 2014 | 2010-2011 | Community- based |
| Heath[149] | United Kingdom & Ireland | 2004 | 2000-2001 | Community- based |
| Brooks[150] | United States | 2005 | 1996-2004 | Community- based |
| Castrodale[151] | United States | 2007 | 2000-2004 | Community- based |
| Chen[152] | United States | 2005 | 1990-2002 | Facility-based |
| Cordero[153] | United States | 2004 | 1986-2002 | Facility-based |
| Hyde[154] | United States | 2002 | 1998-2000 | Community- based |
| Mayor-Lynn[155] | United States | 2005 | 1998-2002 | Facility-based |
| Phares[156] | United States | 2008 | 1999-2005 | Community- based |
| Puopolo[157] | United States | 2005 | 1997-2003 | Facility-based |
| CDC[158] | United States | 2007 | 2003-2005 | Community- based |
| Stoll[159] | United States | 2002 | 1998-2000 | Facility-based |
| Jordan[160] | United States | 2008 | 1990-2005 | Community- based |
| CDC[161] | United States | 2009 | 2000-2006 | Community- based |
| Ecker [162] | United States | 2013 | 1990-2007 | Facility-based |
| Greenhow[163] | United States | 2012 | 2005-2009 | Community- based |
| Greenhow[164] | United States | 2014 | 2005-2011 | Community- based |
| Mukhopadhyay[165] | United States | 2013 | 2008-2009 | Facility-based |
| Mukhopadhyay[166] | United States | 2014 | 2009-2012 | Facility-based |
| Tudela[167] | United States | 2012 | 2000-2008 | Facility-based |

| | | | | |
|---|---|---|---|---|
| Weston[168] | United States | 2011 | 2005-2008 | Community- based |
| Stafford[169] | United States | 2012 | 2000-2008 | Facility-based |
| Stoll[170] | United States | 2011 | 2006-2009 | Facility-based |
| Parente[171] | United States | 2017 | 2002-2012 | Community- based |
| Wortham[172] | United States | 2016 | 2006-2009 | Facility-based |

## Supplementary Figure S1: Incidence of invasive infant GBS disease in studies from United States, 2002-2017, grouped in year periods

| Author | Year | Nºof livebirths | Cases of GBS | ES (95% CI) | % Weight |
|---|---|---|---|---|---|
| 2001-2006 | | | | | |
| Hyde[32] | 2002 | 248184 | 166 | 0.67 (0.57, 0.78) | 4.56 |
| Stoll [37] | 2002 | 6204 | 9 | 1.45 (0.66, 2.75) | 0.75 |
| Puopolo[35] | 2005 | 67260 | 25 | 0.37 (0.24, 0.55) | 4.29 |
| Cordero [31] | 2004 | 59664 | 43 | 0.72 (0.52, 0.97) | 3.84 |
| Chen[30] | 2005 | 120952 | 116 | 0.96 (0.79, 1.15) | 4.14 |
| Brooks [28] | 2005 | 427000 | 308 | 0.72 (0.64, 0.81) | 4.65 |
| Mayor-Lynn[33] | 2005 | 28659 | 61 | 2.13 (1.63, 2.73) | 1.91 |
| Subtotal (I-squared = 88.9%, p = 0.000) | | | | 0.82 (0.62, 1.02) | 24.14 |
| . | | | | | |
| 2007-2011 | | | | | |
| Stoll[48] | 2011 | 396586 | 160 | 0.40 (0.34, 0.47) | 4.71 |
| Jordan [38] | 2008 | 4862508 | 1726 | 0.35 (0.34, 0.37) | 4.79 |
| CDC [36] | 2007 | 1363636 | 450 | 0.33 (0.30, 0.36) | 4.78 |
| Castrodale [29] | 2007 | 39628 | 21 | 0.53 (0.33, 0.81) | 3.73 |
| CDC[39] | 2009 | 2854761 | 2204 | 0.77 (0.74, 0.80) | 4.78 |
| Phares [34] | 2008 | 3047059 | 2268 | 0.74 (0.71, 0.78) | 4.78 |
| Weston [46] | 2011 | 858000 | 249 | 0.29 (0.26, 0.33) | 4.77 |
| Subtotal (I-squared = 99.4%, p = 0.000) | | | | 0.49 (0.32, 0.65) | 32.33 |
| . | | | | | |
| 2012-2017 | | | | | |
| Ecker [40] | 2013 | 45000 | 21 | 0.47 (0.29, 0.71) | 3.92 |
| Tudela [45] | 2012 | 143384 | 94 | 0.66 (0.53, 0.80) | 4.40 |
| Greenhow[41] | 2012 | 160818 | 19 | 0.12 (0.07, 0.18) | 4.73 |
| Parente[49] | 2017 | 642675 | 492 | 0.77 (0.70, 0.84) | 4.69 |
| CDC | 2014 | 434908 | 249 | 0.57 (0.50, 0.65) | 4.68 |
| Stafford[47] | 2012 | 143467 | 94 | 0.66 (0.53, 0.80) | 4.40 |
| Greenhow [42] | 2014 | 224553 | 29 | 0.13 (0.09, 0.19) | 4.74 |
| Wortham[50] | 2016 | 396586 | 78 | 0.20 (0.16, 0.25) | 4.75 |
| Mukhopadhyay[44] | 2014 | 33616 | 48 | 1.43 (1.05, 1.89) | 2.56 |
| Mukhopadhyay[43] | 2013 | 263793 | 95 | 0.36 (0.29, 0.44) | 4.67 |
| Subtotal (I-squared = 97.9%, p = 0.000) | | | | 0.50 (0.33, 0.66) | 43.54 |
| . | | | | | |
| Overall (I-squared = 98.6%, p = 0.000) | | | | 0.57 (0.47, 0.67) | 100.00 |

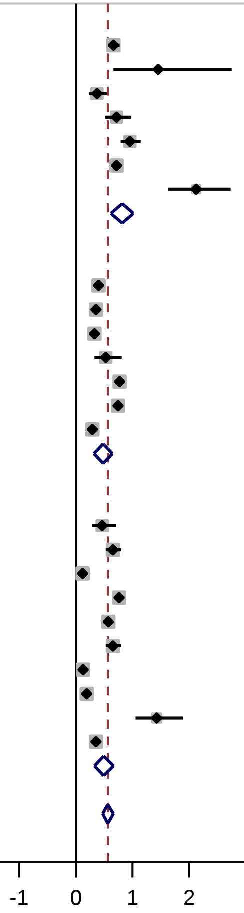


NOTE: Weights are from random effects analysis

Supplementary Figure S2: Publication years of included studies on incidence of invasive infant GBS disease

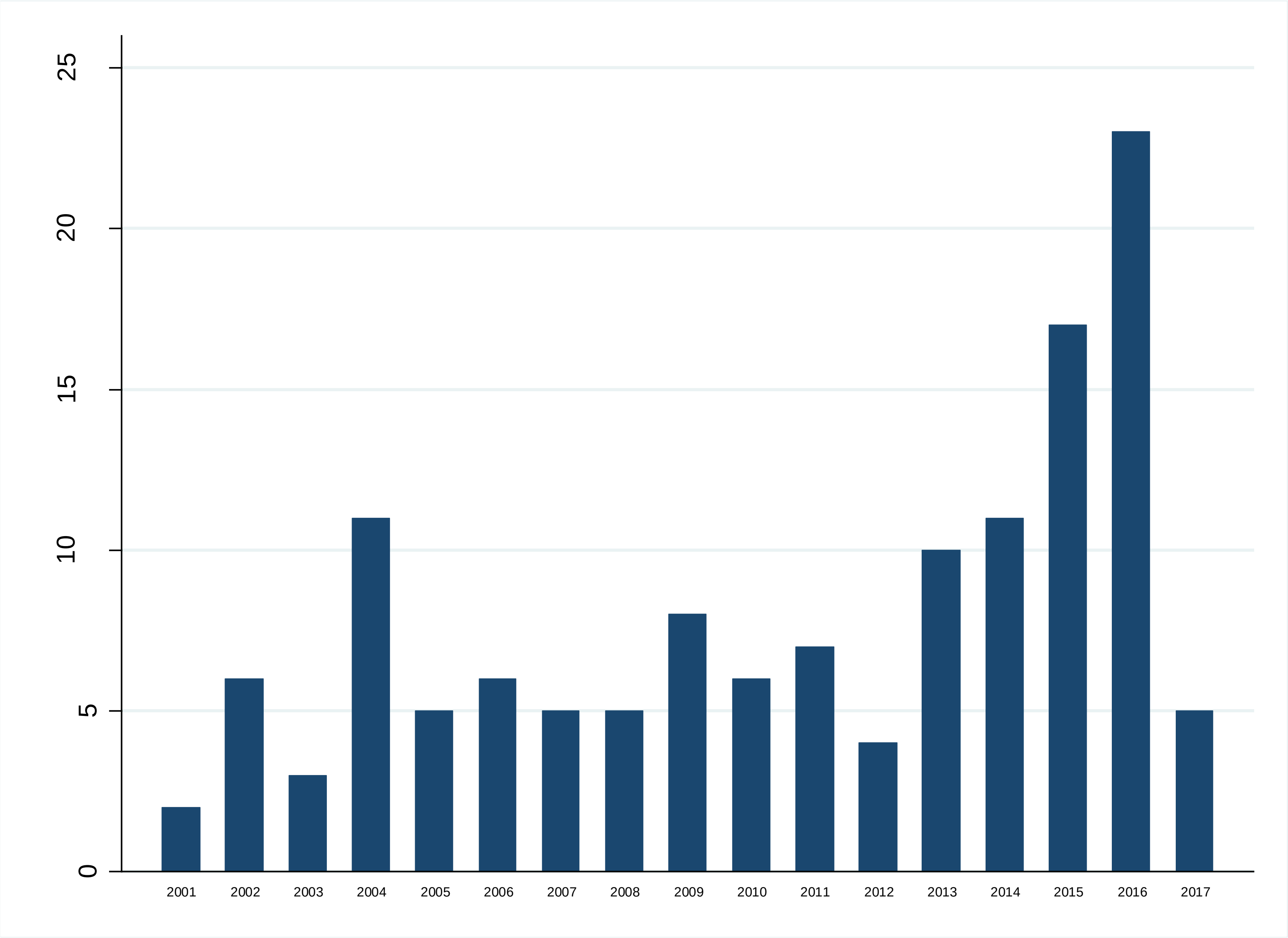

Supplementary Figure S3A. Map illustrating number of studies by country reporting incidence of GBS invasive disease in the previous systematic review by Edmond[1].

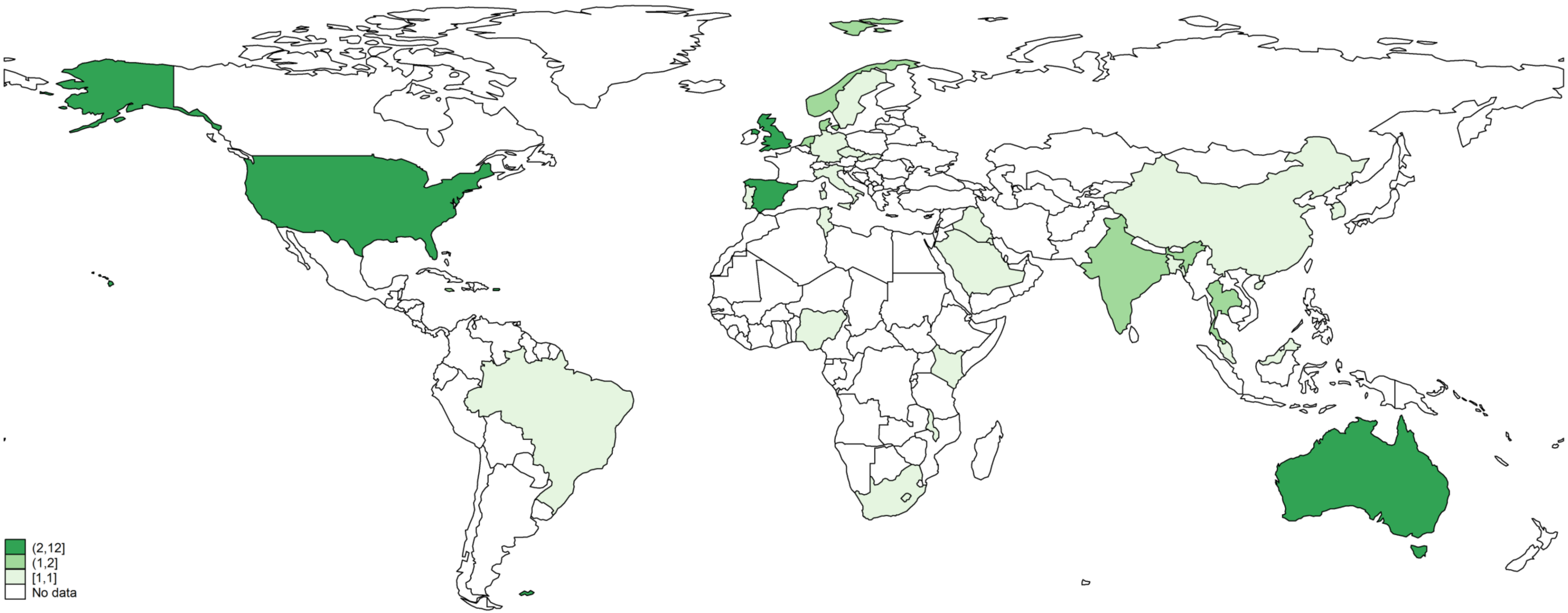

Supplementary Figure S3B. Map illustrating overall incidence of GBS disease among infants aged 0-89 days by country included in the previous meta-analysis by Edmond[1].

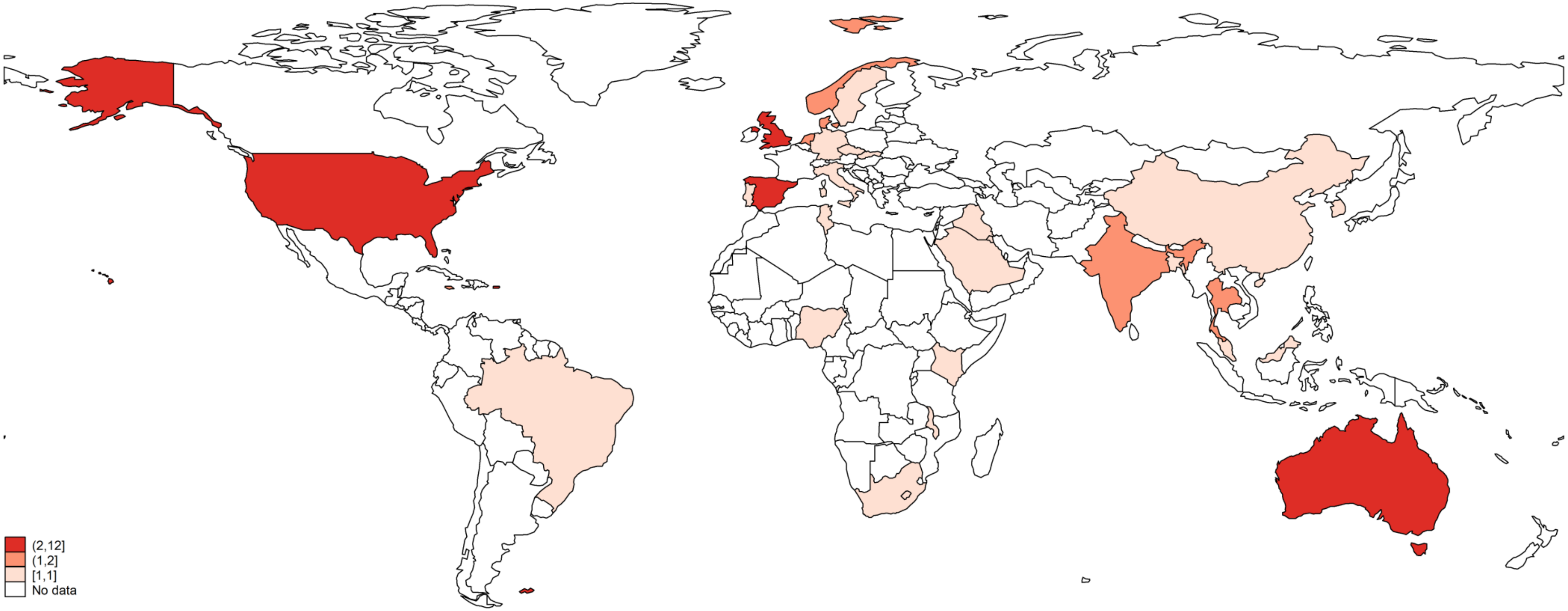

Supplementary Figure S4. Regional distribution of included studies assessing incidence of infant GBS disease, and use of intrapartum antibiotic prophylaxis, 2000-2017. *

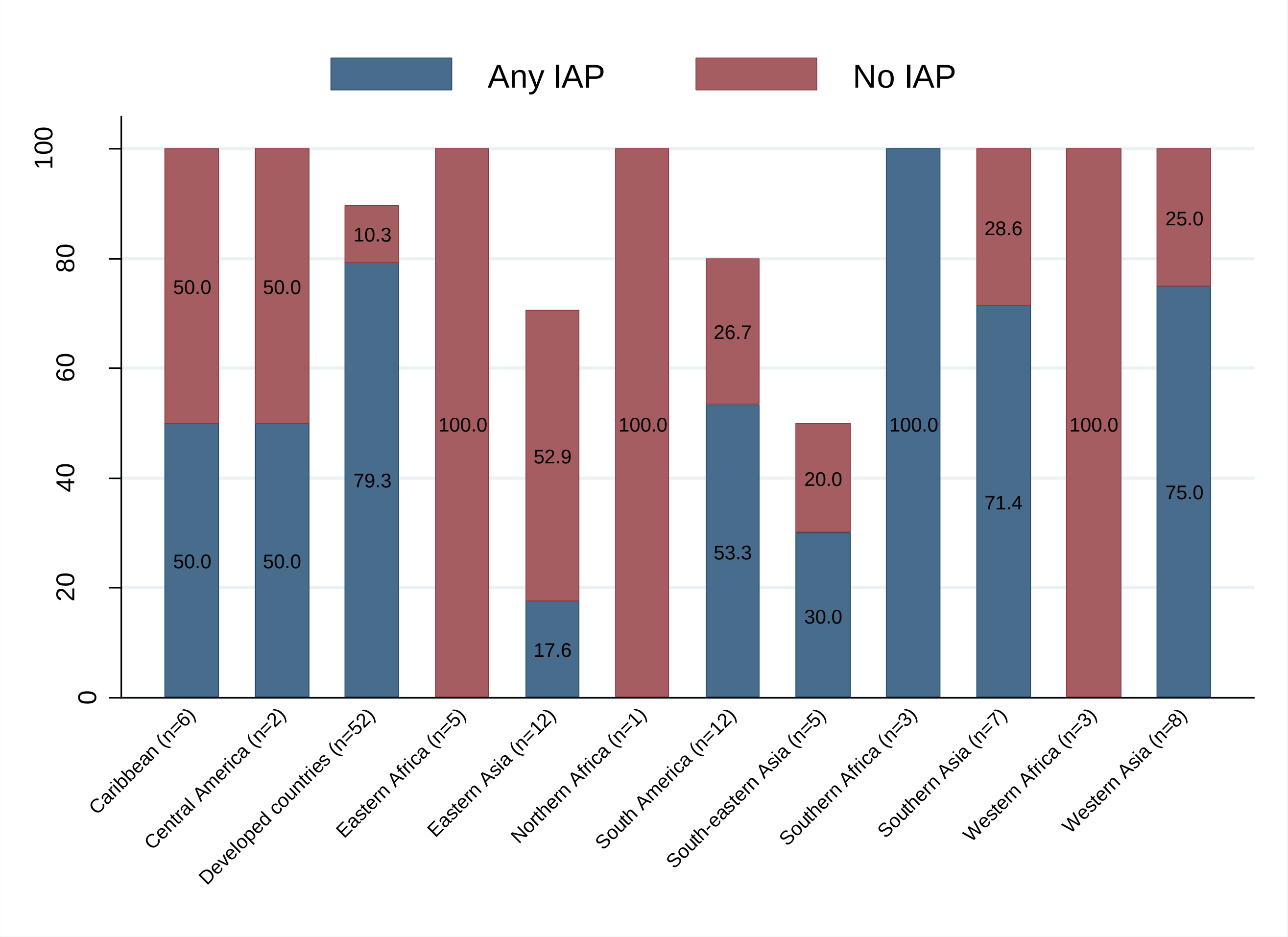


* Information about intrapartum antibiotic prophylaxis (IAP) was available for 116 of 135 studies included in the meta-analysis. Not available information for 6 developed countries,  5 countries from Eastern Asia, 3 countries from South America and 5 countries form South-eastern Asia.

## Supplementary Figure S5: Incidence risk of early-onset GBS disease worldwide

| Country | year | Nº of livebirths | EOD GBS cases | ES (95% CI) | % Weight |
|---|---|---|---|---|---|
| Latin America & the Caribbean | | | | | |
| Jamaica | 2006 | 32029 | 21 | 0.66 (0.41, 1.00) | 1.27 |
| Dominican Republic | 2015 | 17867 | 42 | 2.35 (1.69, 3.18) | 0.43 |
| Panama | 2015 | 15500 | 12 | 0.77 (0.40, 1.35) | 0.80 |
| Brazil | 2012 | 13749 | 7 | 0.51 (0.20, 1.05) | 0.92 |
| Brazil | 2015 | 12585 | 8 | 0.64 (0.27, 1.25) | 0.78 |
| Brazil | 2002 | 111241 | 43 | 0.39 (0.28, 0.52) | 1.87 |
| Argentina | 2005 | 1784 | 0 | 0.00 (0.00, 2.07) | 0.25 |
| Brazil | 2016 | 8818 | 8 | 0.91 (0.39, 1.79) | 0.47 |
| Chile | 2007 | 9002 | 1 | 0.11 (0.00, 0.62) | 1.24 |
| Brazil | 2016 | 4120 | 0 | 0.00 (0.00, 0.89) | 0.86 |
| Brazil | 2016 | 21219 | 7 | 0.33 (0.13, 0.68) | 1.35 |
| Argentina | 2006 | 18444 | 5 | 0.27 (0.09, 0.63) | 1.36 |
| Subtotal (I-squared = 74.5%, p = 0.000) | | | | 0.51 (0.30, 0.71) | 11.60 |
| . | | | | | |
| Developed countries | | | | | |
| Denmark | 2004 | 80263 | 52 | 0.65 (0.48, 0.85) | 1.67 |
| Denmark | 2014 | 33616 | 48 | 1.43 (1.05, 1.89) | 0.93 |
| Canada | 2016 | 53537 | 134 | 2.50 (2.10, 2.96) | 0.90 |
| Israel | 2013 | 91590 | 31 | 0.34 (0.23, 0.48) | 1.85 |
| Spain | 2008 | 107021 | 42 | 0.39 (0.28, 0.53) | 1.86 |
| Australia | 2015 | 847783 | 88 | 0.10 (0.08, 0.13) | 2.04 |
| Spain | 2015 | 168277 | 49 | 0.29 (0.22, 0.38) | 1.96 |
| Norway | 2016 | 168877 | 53 | 0.31 (0.24, 0.41) | 1.95 |
| Italy | 2016 | 146682 | 27 | 0.18 (0.12, 0.27) | 1.98 |
| Canada | 2016 | 50000 | 13 | 0.26 (0.14, 0.44) | 1.77 |
| Japan | 2013 | 438359 | 36 | 0.08 (0.06, 0.11) | 2.04 |
| USA | 2014 | 434908 | 113 | 0.26 (0.21, 0.31) | 2.02 |
| New Zealand | 2016 | 127134 | 29 | 0.23 (0.15, 0.33) | 1.95 |
| Germany | 2006 | 1454520 | 206 | 0.14 (0.12, 0.16) | 2.05 |
| Israel | 2016 | 108000 | 17 | 0.16 (0.09, 0.25) | 1.96 |
| Switzerland | 2016 | 69118 | 8 | 0.12 (0.05, 0.23) | 1.95 |
| UK & Ireland | 2015 | 914132 | 517 | 0.57 (0.52, 0.62) | 2.02 |
| Netherlands | 2010 | 21429 | 15 | 0.70 (0.39, 1.15) | 1.03 |
| Portugal | 2008 | 448531 | 194 | 0.43 (0.37, 0.50) | 2.00 |
| Sweden | 2004 | 72641 | 42 | 0.58 (0.42, 0.78) | 1.67 |
| France | 2010 | 20131 | 15 | 0.75 (0.42, 1.23) | 0.96 |
| Japan | 2012 | 7332 | 4 | 0.55 (0.15, 1.40) | 0.56 |
| Denmark | 2004 | 1203999 | 338 | 0.28 (0.25, 0.31) | 2.04 |
| Netherlands | 2007 | 1000516 | 353 | 0.35 (0.32, 0.39) | 2.03 |
| Subtotal (I-squared = 97.0%, p = 0.000) | | | | 0.37 (0.30, 0.44) | 41.16 |
| . | | | | | |
| Africa | | | | | |
| Malawi | 2007 | 31458 | 29 | 0.92 (0.62, 1.32) | 1.10 |
| Mozambique | 2015 | 47651 | 19 | 0.40 (0.24, 0.62) | 1.64 |
| Kenya | 2016 | 6598 | 5 | 0.76 (0.25, 1.77) | 0.41 |
| Zambia | 2016 | 21120 | 0 | 0.00 (0.00, 0.17) | 1.95 |
| Tunisia | 2008 | 11201 | 11 | 0.98 (0.49, 1.76) | 0.55 |
| South Africa | 2016 | 316767 | 447 | 1.41 (1.28, 1.55) | 1.83 |
| South Africa | 2015 | 142700 | 214 | 1.50 (1.31, 1.71) | 1.59 |
| Nigeria | 2016 | 908 | 0 | 0.00 (0.00, 4.05) | 0.07 |
| Nigeria | 2005 | 4135 | 1 | 0.24 (0.01, 1.35) | 0.50 |
| Gambia | 2016 | 750 | 1 | 1.33 (0.03, 7.41) | 0.02 |
| South Africa | 2014 | 49030 | 14 | 0.29 (0.16, 0.48) | 1.74 |
| Subtotal (I-squared = 97.6%, p = 0.000) | | | | 0.71 (0.24, 1.17) | 11.41 |
| . | | | | | |
| Asia | | | | | |
| China | 2013 | 76141 | 24 | 0.32 (0.20, 0.47) | 1.83 |
| China | 2015 | 10924 | 2 | 0.18 (0.02, 0.66) | 1.20 |
| Hong Kong | 2015 | 13244 | 10 | 0.76 (0.36, 1.39) | 0.73 |
| Macau | 2009 | 6400 | 2 | 0.31 (0.04, 1.13) | 0.67 |
| Taiwan | 2016 | 11123 | 0 | 0.00 (0.00, 0.33) | 1.73 |
| Malaysia | 2013 | 25768 | 20 | 0.78 (0.47, 1.20) | 1.08 |
| Malaysia | 2009 | 46140 | 17 | 0.37 (0.21, 0.59) | 1.65 |
| Philippines | 2015 | 11768 | 3 | 0.25 (0.05, 0.74) | 1.12 |
| Thailand | 2013 | 2849 | 3 | 1.05 (0.22, 3.07) | 0.14 |
| Thailand | 2002 | 62761 | 17 | 0.27 (0.16, 0.43) | 1.81 |
| Thailand | 2015 | 8409 | 2 | 0.24 (0.03, 0.86) | 0.94 |
| Thailand | 2009 | 21299 | 3 | 0.14 (0.03, 0.41) | 1.64 |
| Vietnam | 2013 | 39529 | 5 | 0.13 (0.04, 0.30) | 1.85 |
| Thailand | 2014 | 41483 | 1 | 0.02 (0.00, 0.13) | 1.99 |
| Bangladesh | 2016 | 19230 | 5 | 0.26 (0.08, 0.61) | 1.40 |
| India | 2016 | 1416 | 2 | 1.41 (0.17, 5.09) | 0.05 |
| India | 2009 | 34362 | 0 | 0.00 (0.00, 0.11) | 2.01 |
| India | 2015 | 88636 | 8 | 0.09 (0.04, 0.18) | 1.99 |
| India | 2009 | 4689 | 0 | 0.00 (0.00, 0.79) | 0.99 |
| India | 2014 | 107692 | 73 | 0.68 (0.53, 0.85) | 1.74 |
| Bangladesh | 2009 | 10000 | 1 | 0.10 (0.00, 0.56) | 1.34 |
| Qatar | 2011 | 87260 | 45 | 0.52 (0.38, 0.69) | 1.75 |
| Saudi Arabia | 2017 | 30389 | 20 | 0.66 (0.40, 1.02) | 1.24 |
| Kuwait | 2009 | 44990 | 14 | 0.31 (0.17, 0.52) | 1.69 |
| United Arab Emirates | 2017 | 13584 | 8 | 0.59 (0.25, 1.16) | 0.85 |
| Kuwait | 2017 | 23501 | 33 | 1.40 (0.97, 1.97) | 0.75 |
| Kuwait | 2011 | 56134 | 27 | 0.48 (0.32, 0.70) | 1.64 |
| Subtotal (I-squared = 87.0%, p = 0.000) | | | | 0.31 (0.22, 0.41) | 35.83 |
| . | | | | | |
| Overall (I-squared = 95.3%, p = 0.000) | | | | 0.41 (0.36, 0.46) | 100.00 |

NOTE: Weights are from random effects analysis

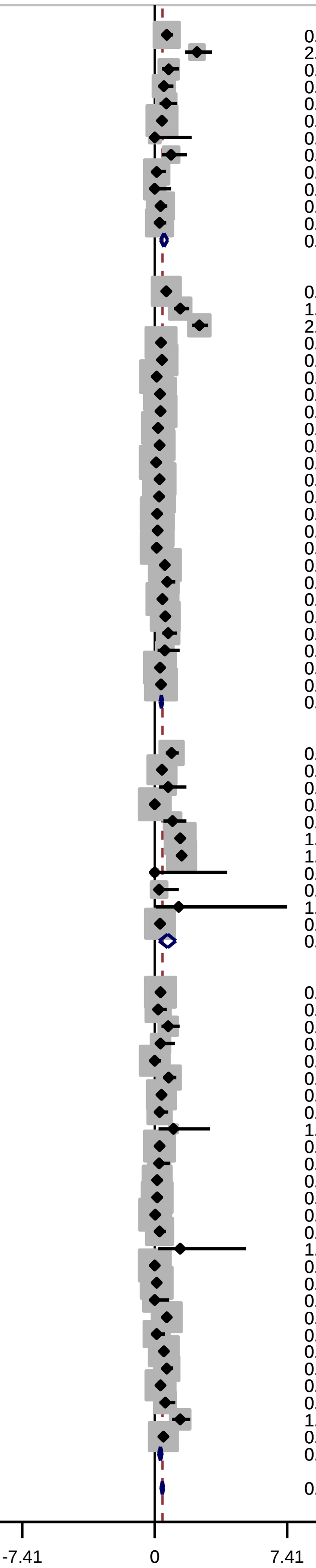

Supplementary Figure S6: Percentage of early-onset GBS disease cases in the first 24 hours after birth, of all early-onset (days 0-6) cases.

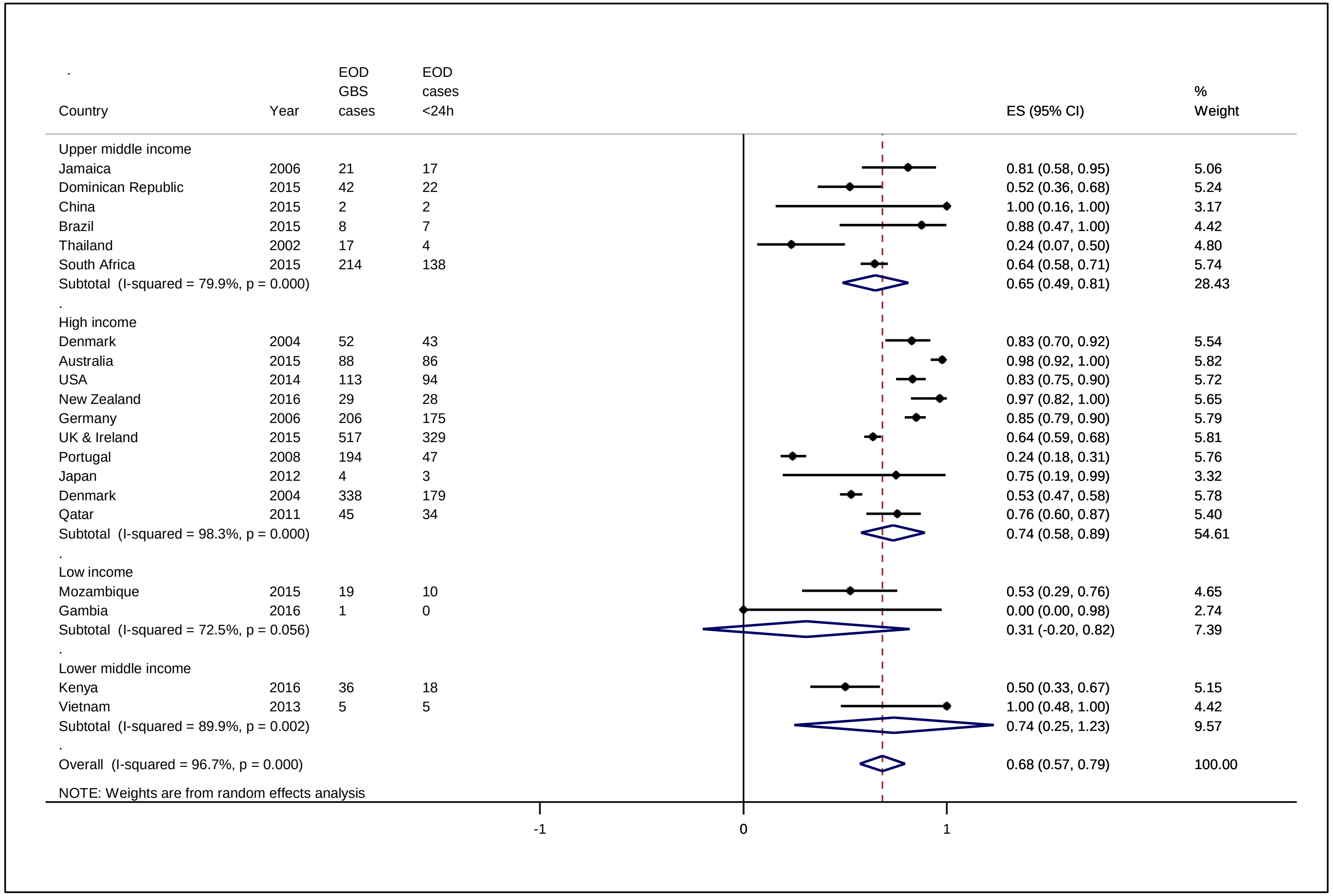

## Supplementary Figure S7: Incidence risk of late-onset GBS disease worldwide by region

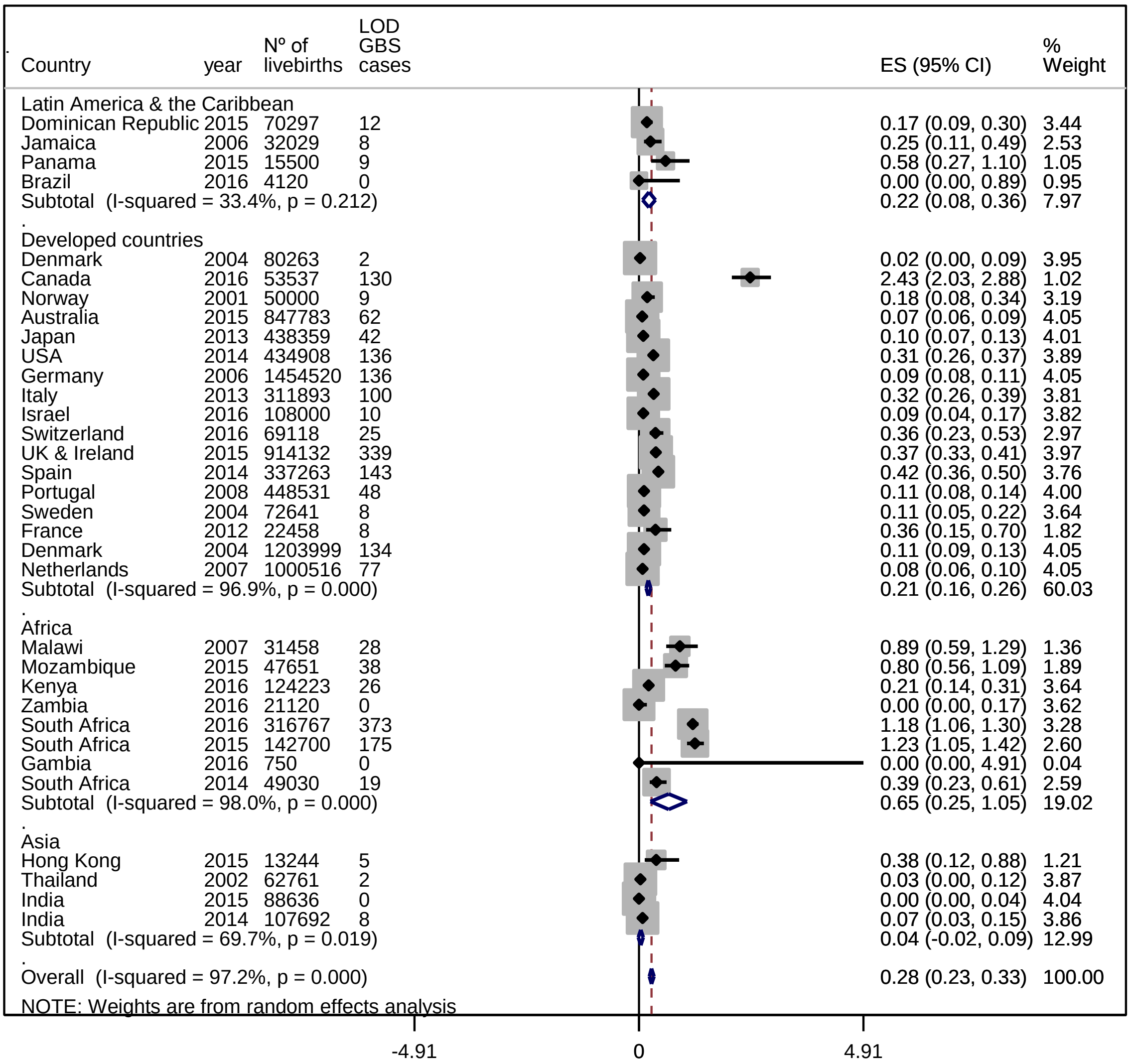

## Supplementary Figure S8: Case fatality risk of GBS disease in infants aged 0-89 days worldwide by region

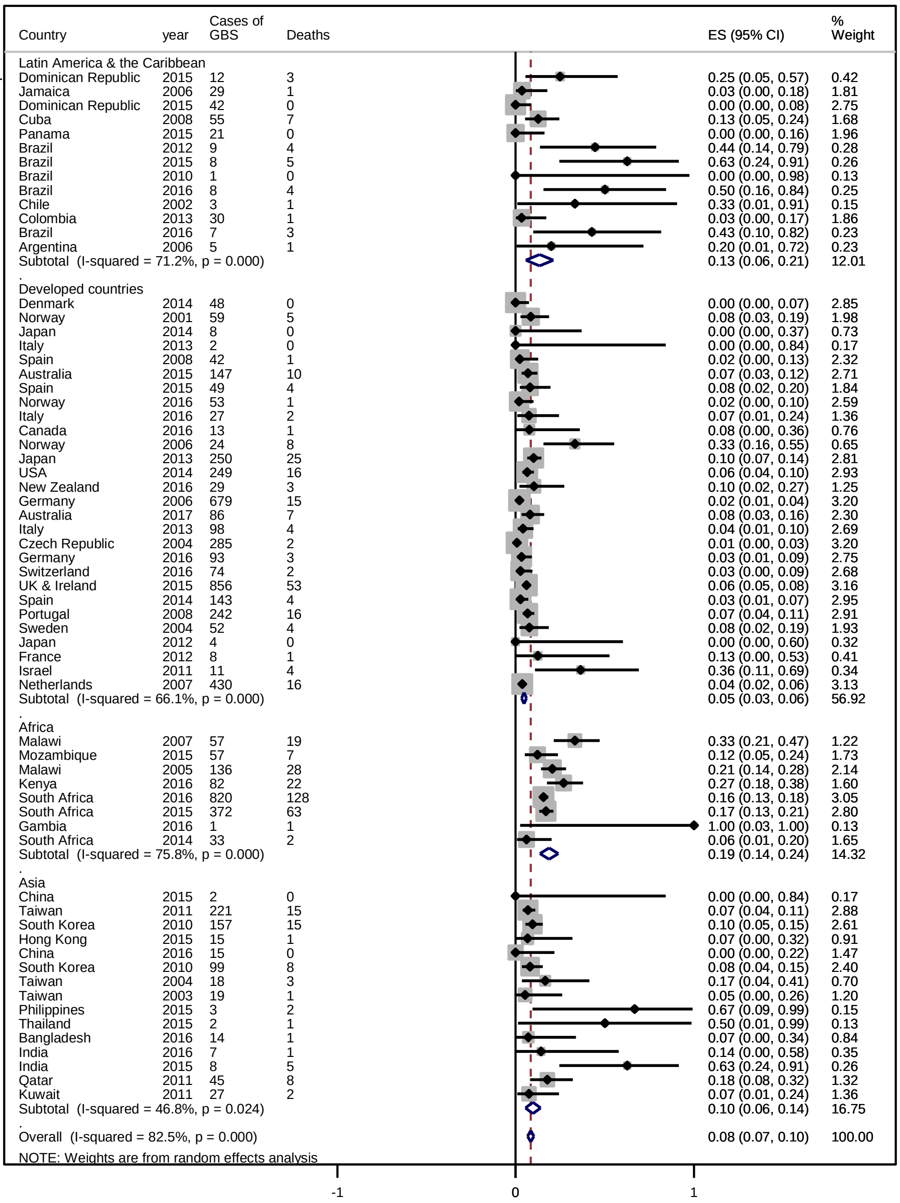

## Supplementary Figure S9: Case fatality risk of early-onset GBS disease by region.

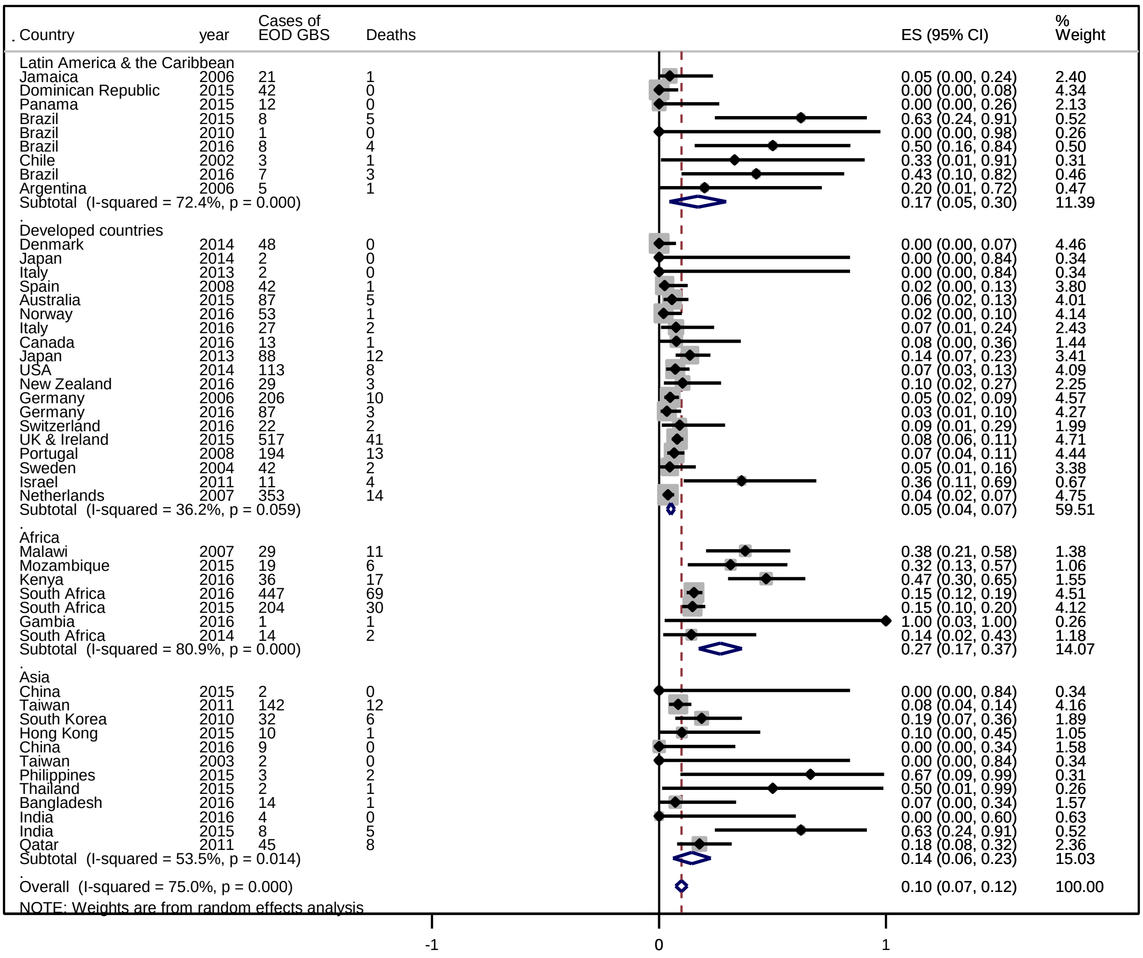

## Supplementary Figure S10: Case fatality risk of late-onset GBS disease by region.

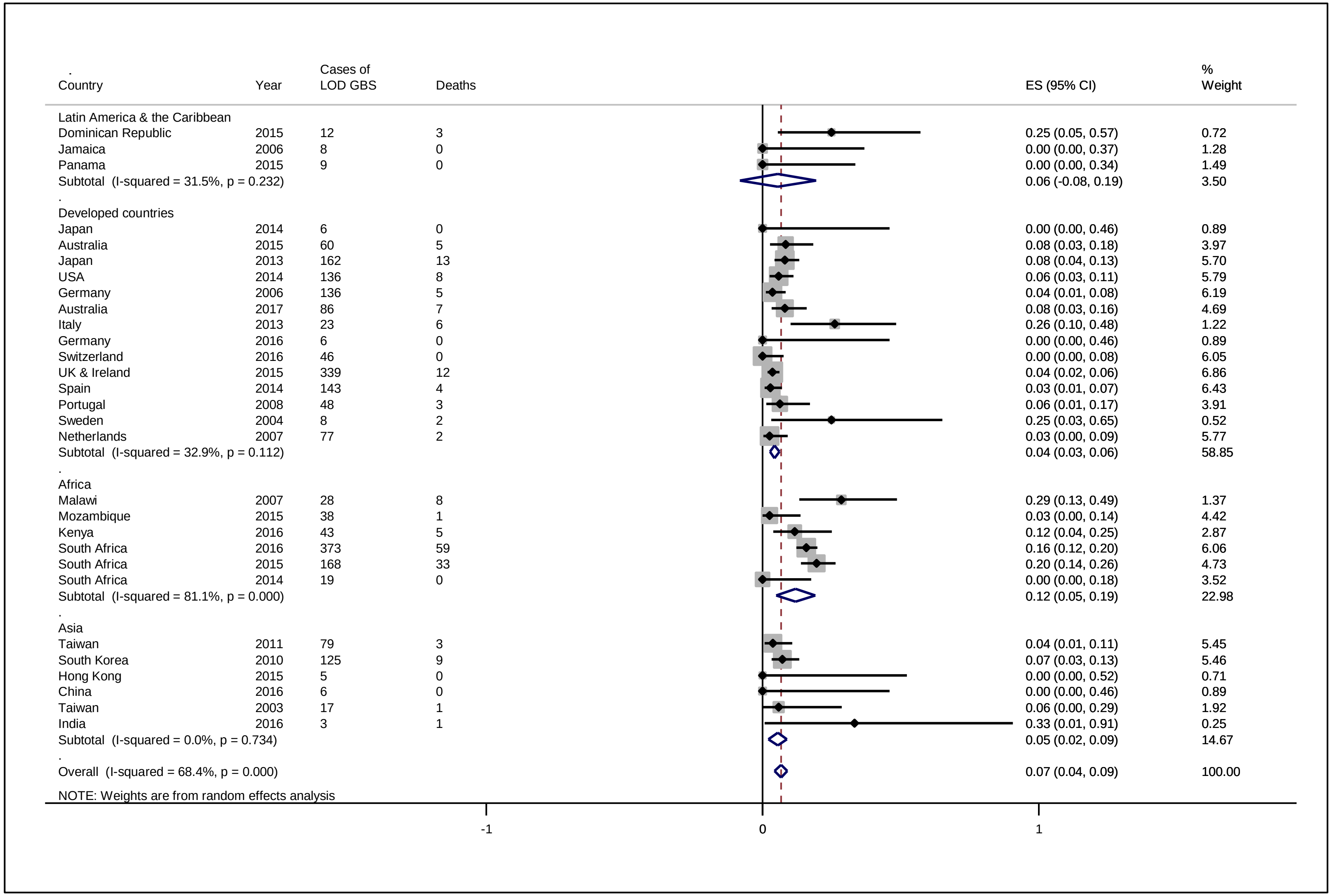

Supplementary Figure S11:  Map of included studies reporting serotype data for infant GBS disease

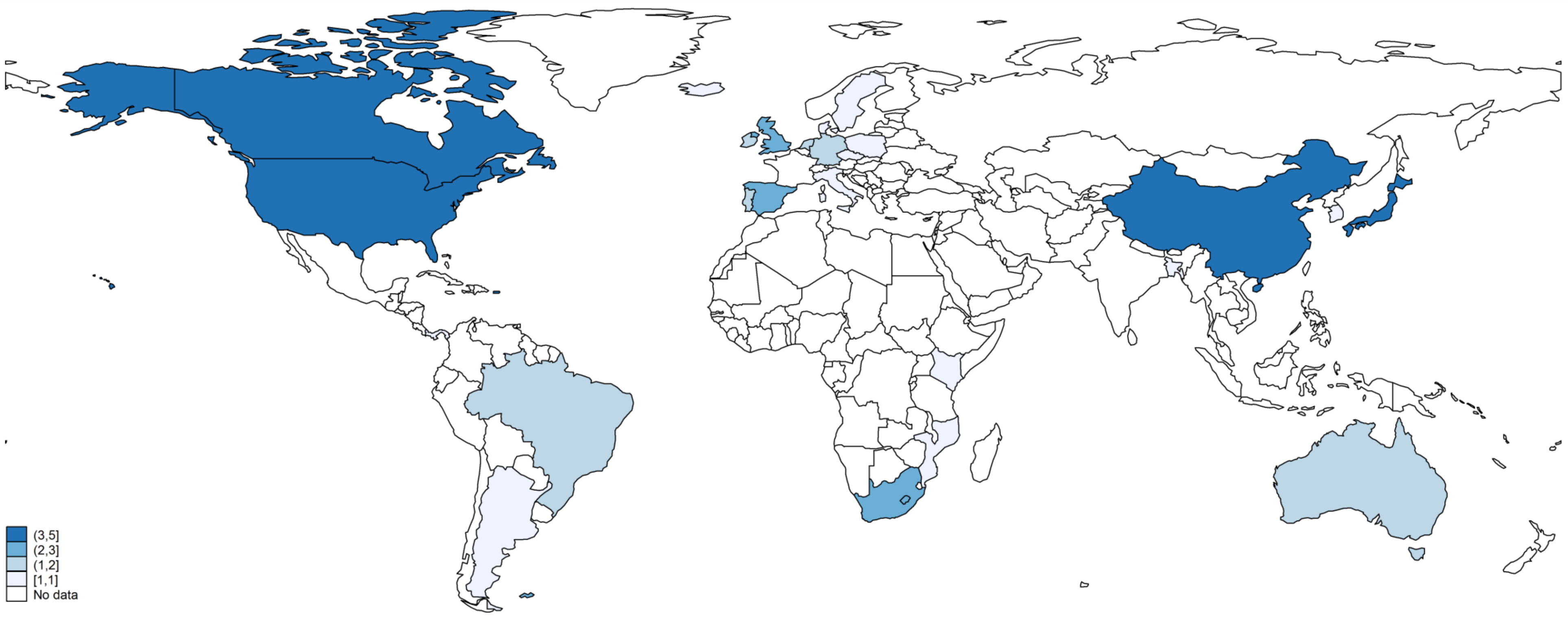

Supplementary Figure S12: Distribution of GBS serotypes for A) early onset GBS disease and B) late onset GBS disease

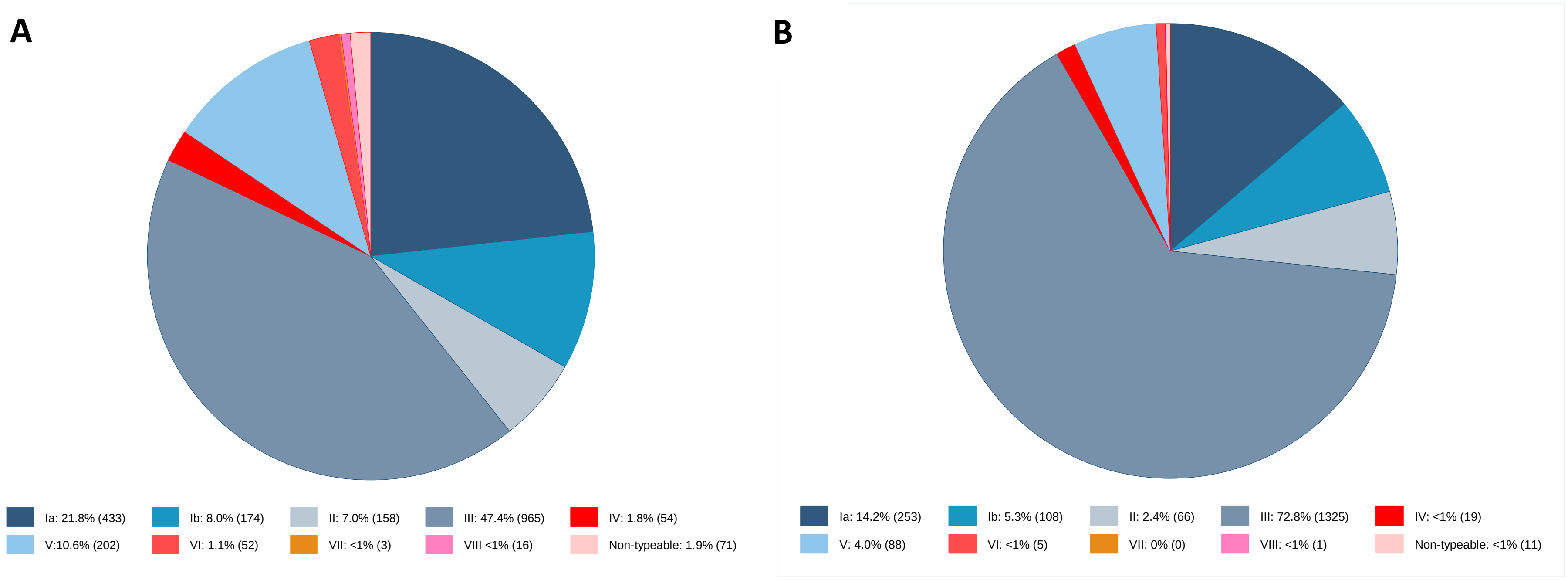


*Figures presenting prevalence of serotype in % (n).

## Supplementary Figure S13A: Ratio of early to late-onset GBS disease cases among infant GBS disease cases

| Country | Year | Nº of livebirths | GBS cases | RR (95% CI) | % Weight |
|---|---|---|---|---|---|
| Developed countries | | | | | |
| UK & Ireland | 2015 | 914132 | 856 | 1.53 (1.33, 1.75) | 5.44 |
| Australia | 2015 | 847783 | 150 | 1.42 (1.03, 1.96) | 5.05 |
| Canada | 2016 | 1015385 | 264 | 1.03 (0.81, 1.31) | 5.26 |
| Switzerland | 2016 | 69118 | 33 | 0.32 (0.14, 0.71) | 3.52 |
| Germany | 2006 | 1454520 | 312 | 1.51 (1.22, 1.88) | 5.31 |
| Denmark | 2004 | 1203999 | 472 | 2.52 (2.07, 3.08) | 5.34 |
| Denmark | 2004 | 80263 | 54 | 26.00 (6.33, 106.74) | 1.98 |
| Israel | 2016 | 108000 | 27 | 1.70 (0.78, 3.71) | 3.57 |
| Japan | 2013 | 438359 | 78 | 0.86 (0.55, 1.34) | 4.70 |
| Netherlands | 2007 | 1000516 | 430 | 4.58 (3.58, 5.87) | 5.25 |
| Portugal | 2008 | 448531 | 242 | 4.04 (2.95, 5.54) | 5.08 |
| Sweden | 2004 | 72641 | 50 | 5.25 (2.47, 11.18) | 3.65 |
| USA | 2014 | 434908 | 249 | 0.83 (0.65, 1.07) | 5.24 |
| Subtotal (I-squared = 94.3%, p = 0.000) | | | | 1.82 (1.29, 2.57) | 59.39 |
| . | | | | | |
| Latin America & the Caribbean | | | | | |
| Jamaica | 2006 | 32029 | 29 | 2.63 (1.16, 5.93) | 3.47 |
| Panama | 2015 | 15500 | 21 | 1.33 (0.56, 3.16) | 3.31 |
| Brazil | 2016 | 4120 | 0 | (Excluded) | 0.00 |
| Subtotal (I-squared = 20.2%, p = 0.263) | | | | 1.90 (0.98, 3.69) | 6.77 |
| . | | | | | |
| Asia | | | | | |
| Hong Kong | 2015 | 13244 | 15 | 2.00 (0.68, 5.85) | 2.72 |
| India | 2014 | 107692 | 81 | 9.13 (4.40, 18.93) | 3.74 |
| India | 2015 | 88636 | 8 | 17.00 (0.98, 294.53) | 0.66 |
| Thailand | 2002 | 62761 | 19 | 8.50 (1.96, 36.79) | 1.89 |
| Subtotal (I-squared = 51.5%, p = 0.103) | | | | 5.99 (2.40, 14.92) | 9.01 |
| . | | | | | |
| Africa | | | | | |
| Gambia | 2016 | 750 | 1 | 3.00 (0.12, 73.52) | 0.54 |
| Kenya | 2016 | 124223 | 42 | 0.84 (0.54, 1.30) | 4.70 |
| Mozambique | 2015 | 47651 | 57 | 0.50 (0.29, 0.87) | 4.35 |
| Malawi | 2007 | 31458 | 57 | 1.04 (0.62, 1.74) | 4.45 |
| South Africa | 2015 | 142700 | 389 | 1.22 (1.00, 1.49) | 5.34 |
| South Africa | 2016 | 316767 | 820 | 1.20 (1.04, 1.37) | 5.44 |
| Zambia | 2016 | 21120 | 0 | (Excluded) | 0.00 |
| Subtotal (I-squared = 58.1%, p = 0.036) | | | | 1.02 (0.82, 1.27) | 24.83 |
| . | | | | | |
| Overall (I-squared = 91.9%, p = 0.000) | | | | 1.72 (1.35, 2.21) | 100.00 |

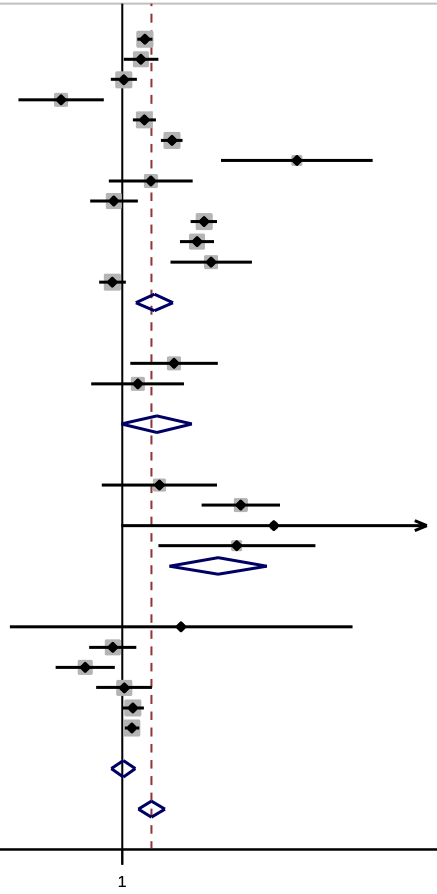


NOTE: Weights are from random effects analysis

Supplementary Figure S13B: Early to late-onset ratio for infant invasive GBS disease in high quality studies.

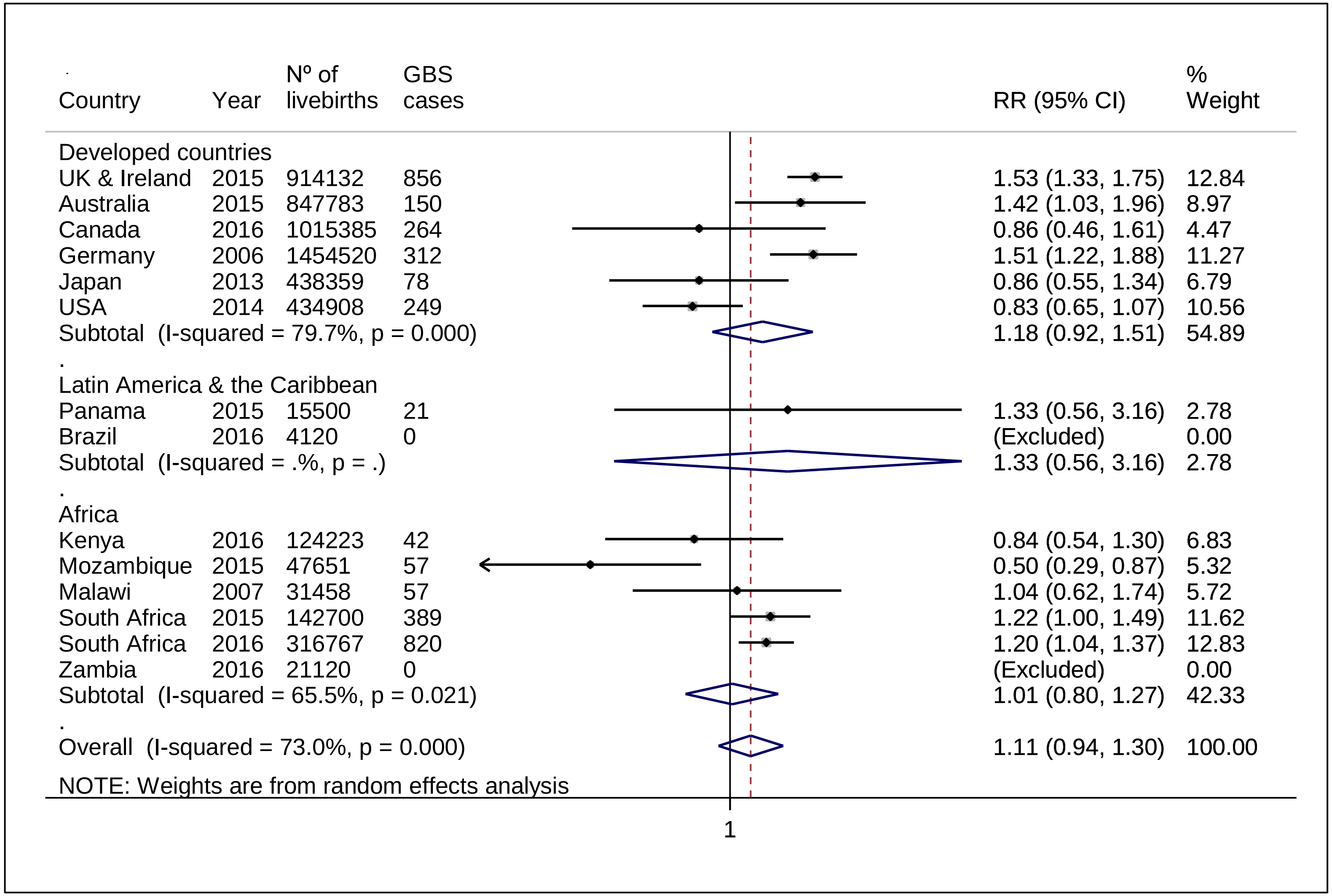


*Analysis was restricted to those studies reporting early to late-onset ratio between 0.5 and 1.5 based on high quality studies in high, lower-middle and upper-middle income countries[93-96].

Supplementary Figure S14: Meningitis cases among early-onset GBS cases.

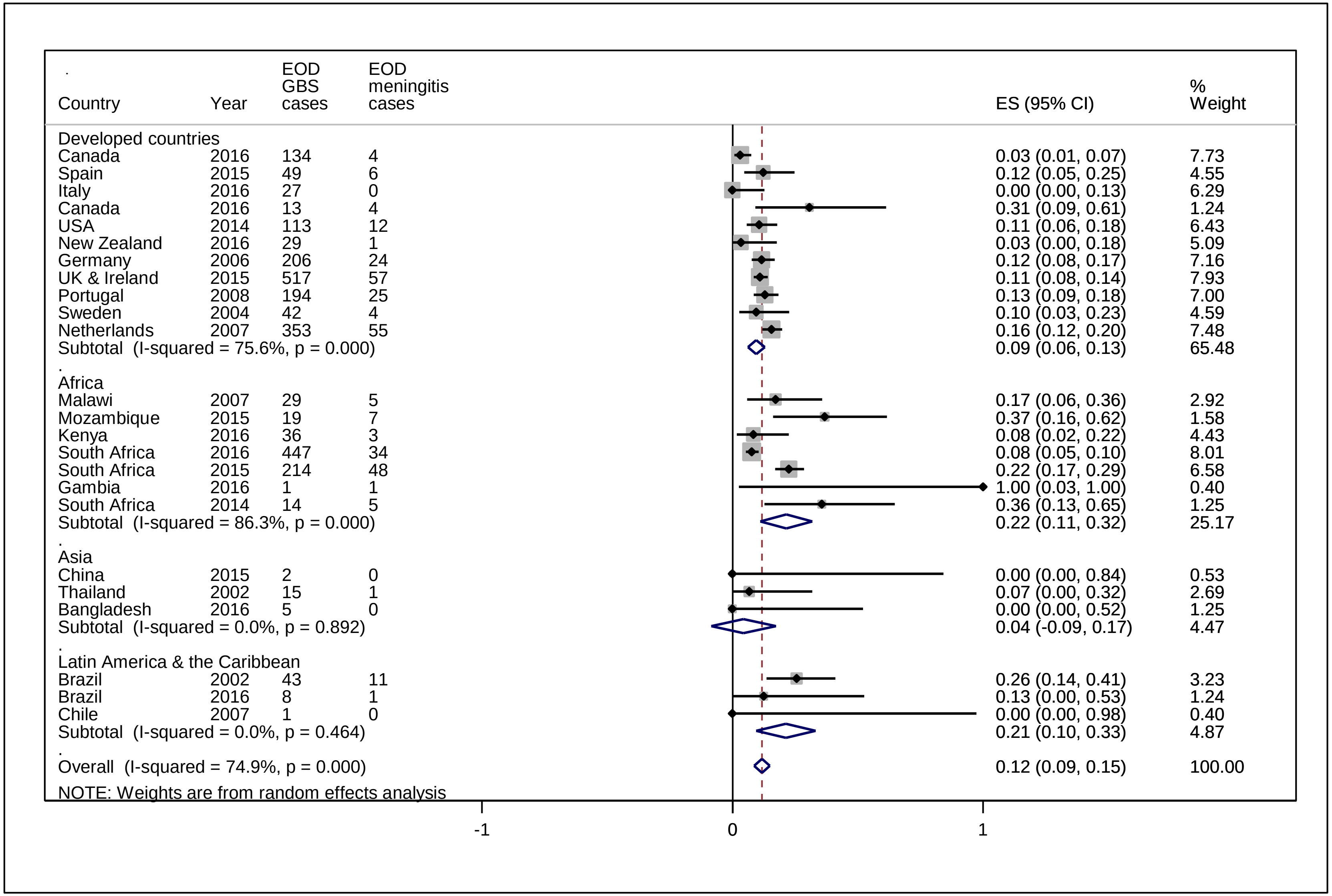

Supplementary Figure S15: Meningitis cases among late-onset GBS cases

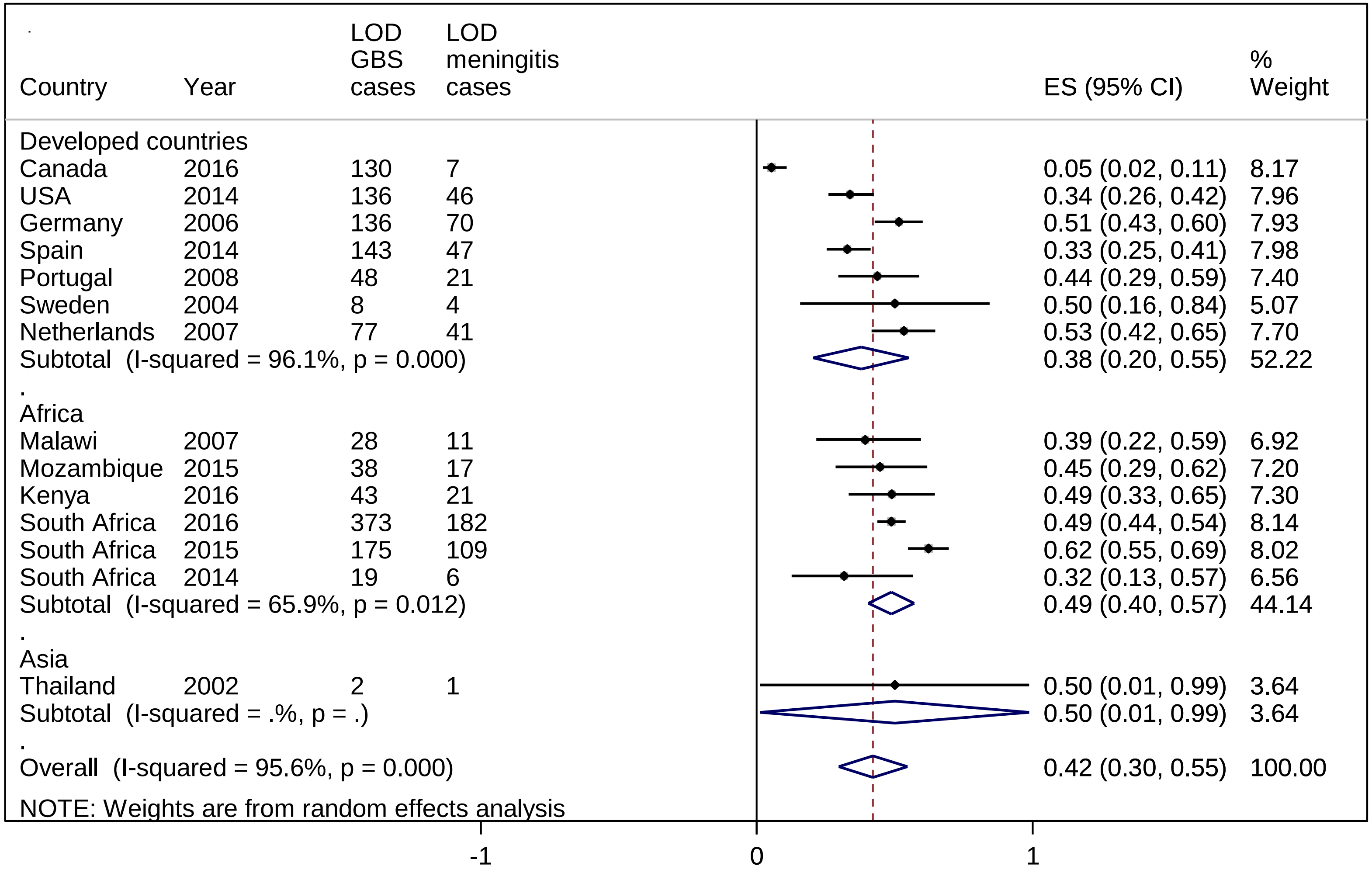

## Supplementary Figure S16: Incidence of GBS disease among infants aged 0-89 days in facility-based studies by region.

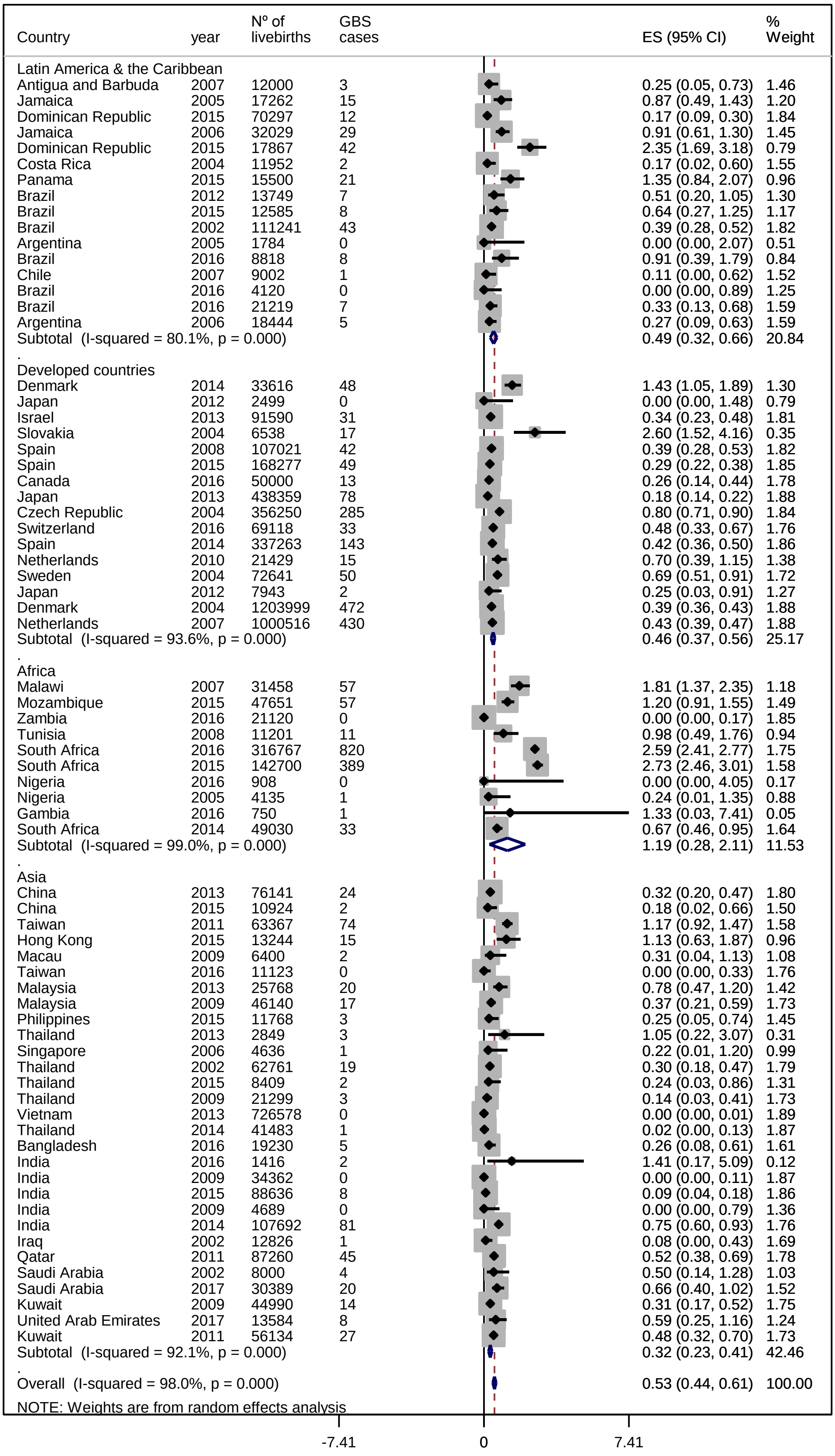

## Supplementary Figure S17: Incidence of early-onset GBS disease among infants in facility-based studies by region

| Country | year | Nº of livebirths | EOD GBS cases | ES (95% CI) | % Weight |
|---|---|---|---|---|---|
| **Latin America & the Caribbean** | | | | | |
| Jamaica | 2006 | 32029 | 21 | 0.66 (0.41, 1.00) | 1.73 |
| Dominican Republic | 2015 | 17867 | 42 | 2.35 (1.69, 3.18) | 0.69 |
| Panama | 2015 | 15500 | 12 | 0.77 (0.40, 1.35) | 1.19 |
| Brazil | 2012 | 13749 | 7 | 0.51 (0.20, 1.05) | 1.34 |
| Brazil | 2015 | 12585 | 8 | 0.64 (0.27, 1.25) | 1.16 |
| Brazil | 2002 | 111241 | 43 | 0.39 (0.28, 0.52) | 2.30 |
| Argentina | 2005 | 1784 | 0 | 0.00 (0.00, 2.07) | 0.41 |
| Brazil | 2016 | 8818 | 8 | 0.91 (0.39, 1.79) | 0.75 |
| Chile | 2007 | 9002 | 1 | 0.11 (0.00, 0.62) | 1.70 |
| Brazil | 2016 | 4120 | 0 | 0.00 (0.00, 0.89) | 1.26 |
| Brazil | 2016 | 21219 | 7 | 0.33 (0.13, 0.68) | 1.82 |
| Argentina | 2006 | 18444 | 5 | 0.27 (0.09, 0.63) | 1.82 |
| Subtotal (I-squared = 74.5%, p = 0.000) | | | | 0.51 (0.30, 0.71) | 16.15 |
| . | | | | | |
| **Developed countries** | | | | | |
| Denmark | 2014 | 33616 | 48 | 1.43 (1.05, 1.89) | 1.34 |
| Israel | 2013 | 91590 | 31 | 0.34 (0.23, 0.48) | 2.29 |
| Spain | 2008 | 107021 | 42 | 0.39 (0.28, 0.53) | 2.29 |
| Spain | 2015 | 168277 | 49 | 0.29 (0.22, 0.38) | 2.37 |
| Canada | 2016 | 50000 | 13 | 0.26 (0.14, 0.44) | 2.21 |
| Japan | 2013 | 438359 | 36 | 0.08 (0.06, 0.11) | 2.44 |
| Switzerland | 2016 | 69118 | 8 | 0.12 (0.05, 0.23) | 2.37 |
| Netherlands | 2010 | 21429 | 15 | 0.70 (0.39, 1.15) | 1.46 |
| Sweden | 2004 | 72641 | 42 | 0.58 (0.42, 0.78) | 2.12 |
| Japan | 2012 | 7332 | 4 | 0.55 (0.15, 1.40) | 0.87 |
| Denmark | 2004 | 1203999 | 338 | 0.28 (0.25, 0.31) | 2.44 |
| Netherlands | 2007 | 1000516 | 353 | 0.35 (0.32, 0.39) | 2.44 |
| Subtotal (I-squared = 95.2%, p = 0.000) | | | | 0.35 (0.26, 0.45) | 24.64 |
| . | | | | | |
| **Africa** | | | | | |
| Malawi | 2007 | 31458 | 29 | 0.92 (0.62, 1.32) | 1.55 |
| Mozambique | 2015 | 47651 | 19 | 0.40 (0.24, 0.62) | 2.09 |
| Zambia | 2016 | 21120 | 0 | 0.00 (0.00, 0.17) | 2.37 |
| Tunisia | 2008 | 11201 | 11 | 0.98 (0.49, 1.76) | 0.85 |
| South Africa | 2016 | 316767 | 447 | 1.41 (1.28, 1.55) | 2.27 |
| South Africa | 2015 | 142700 | 214 | 1.50 (1.31, 1.71) | 2.05 |
| Nigeria | 2016 | 908 | 0 | 0.00 (0.00, 4.05) | 0.12 |
| Nigeria | 2005 | 4135 | 1 | 0.24 (0.01, 1.35) | 0.79 |
| Gambia | 2016 | 750 | 1 | 1.33 (0.03, 7.41) | 0.04 |
| South Africa | 2014 | 49030 | 14 | 0.29 (0.16, 0.48) | 2.19 |
| Subtotal (I-squared = 97.8%, p = 0.000) | | | | 0.70 (0.21, 1.19) | 14.31 |
| . | | | | | |
| **Asia** | | | | | |
| China | 2013 | 76141 | 24 | 0.32 (0.20, 0.47) | 2.26 |
| China | 2015 | 10924 | 2 | 0.18 (0.02, 0.66) | 1.66 |
| Hong Kong | 2015 | 13244 | 10 | 0.76 (0.36, 1.39) | 1.10 |
| Macau | 2009 | 6400 | 2 | 0.31 (0.04, 1.13) | 1.02 |
| Taiwan | 2016 | 11123 | 0 | 0.00 (0.00, 0.33) | 2.17 |
| Malaysia | 2013 | 25768 | 20 | 0.78 (0.47, 1.20) | 1.52 |
| Malaysia | 2009 | 46140 | 17 | 0.37 (0.21, 0.59) | 2.11 |
| Philippines | 2015 | 11768 | 3 | 0.25 (0.05, 0.74) | 1.57 |
| Thailand | 2013 | 2849 | 3 | 1.05 (0.22, 3.07) | 0.23 |
| Thailand | 2002 | 62761 | 17 | 0.27 (0.16, 0.43) | 2.25 |
| Thailand | 2015 | 8409 | 2 | 0.24 (0.03, 0.86) | 1.36 |
| Thailand | 2009 | 21299 | 3 | 0.14 (0.03, 0.41) | 2.09 |
| Vietnam | 2013 | 39529 | 5 | 0.13 (0.04, 0.30) | 2.28 |
| Thailand | 2014 | 41483 | 1 | 0.02 (0.00, 0.13) | 2.40 |
| Bangladesh | 2016 | 19230 | 5 | 0.26 (0.08, 0.61) | 1.86 |
| India | 2016 | 1416 | 2 | 1.41 (0.17, 5.09) | 0.08 |
| India | 2009 | 34362 | 0 | 0.00 (0.00, 0.11) | 2.42 |
| India | 2015 | 88636 | 8 | 0.09 (0.04, 0.18) | 2.40 |
| India | 2009 | 4689 | 0 | 0.00 (0.00, 0.79) | 1.42 |
| India | 2014 | 107692 | 73 | 0.68 (0.53, 0.85) | 2.19 |
| Qatar | 2011 | 87260 | 45 | 0.52 (0.38, 0.69) | 2.20 |
| Saudi Arabia | 2017 | 30389 | 20 | 0.66 (0.40, 1.02) | 1.70 |
| Kuwait | 2009 | 44990 | 14 | 0.31 (0.17, 0.52) | 2.14 |
| United Arab Emirates | 2017 | 13584 | 8 | 0.59 (0.25, 1.16) | 1.25 |
| Kuwait | 2017 | 23501 | 33 | 1.40 (0.97, 1.97) | 1.12 |
| Kuwait | 2011 | 56134 | 27 | 0.48 (0.32, 0.70) | 2.09 |
| Subtotal (I-squared = 87.5%, p = 0.000) | | | | 0.32 (0.23, 0.42) | 44.90 |
| . | | | | | |
| Overall (I-squared = 94.3%, p = 0.000) | | | | 0.43 (0.35, 0.50) | 100.00 |

NOTE: Weights are from random effects analysis

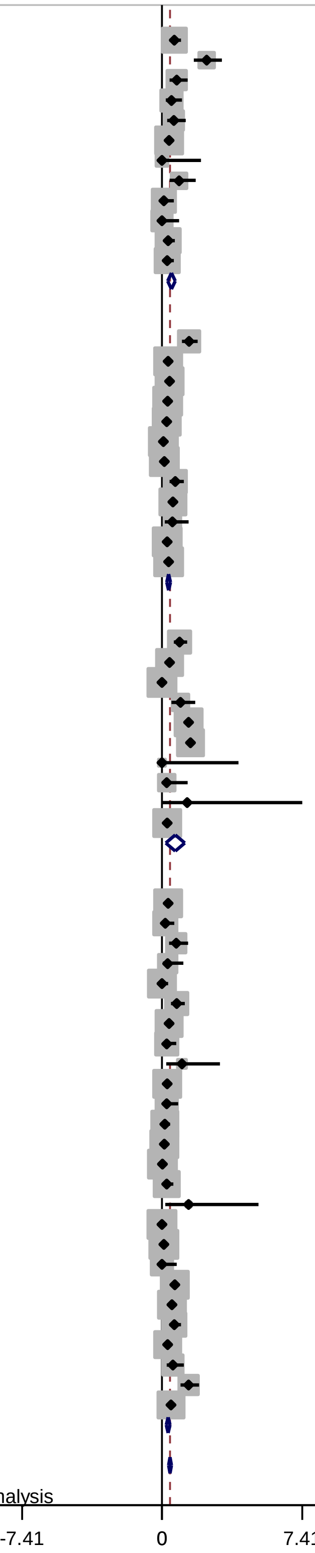

Supplementary Figure S18: Incidence of late-onset GBS disease among infants in facility-based studies by region

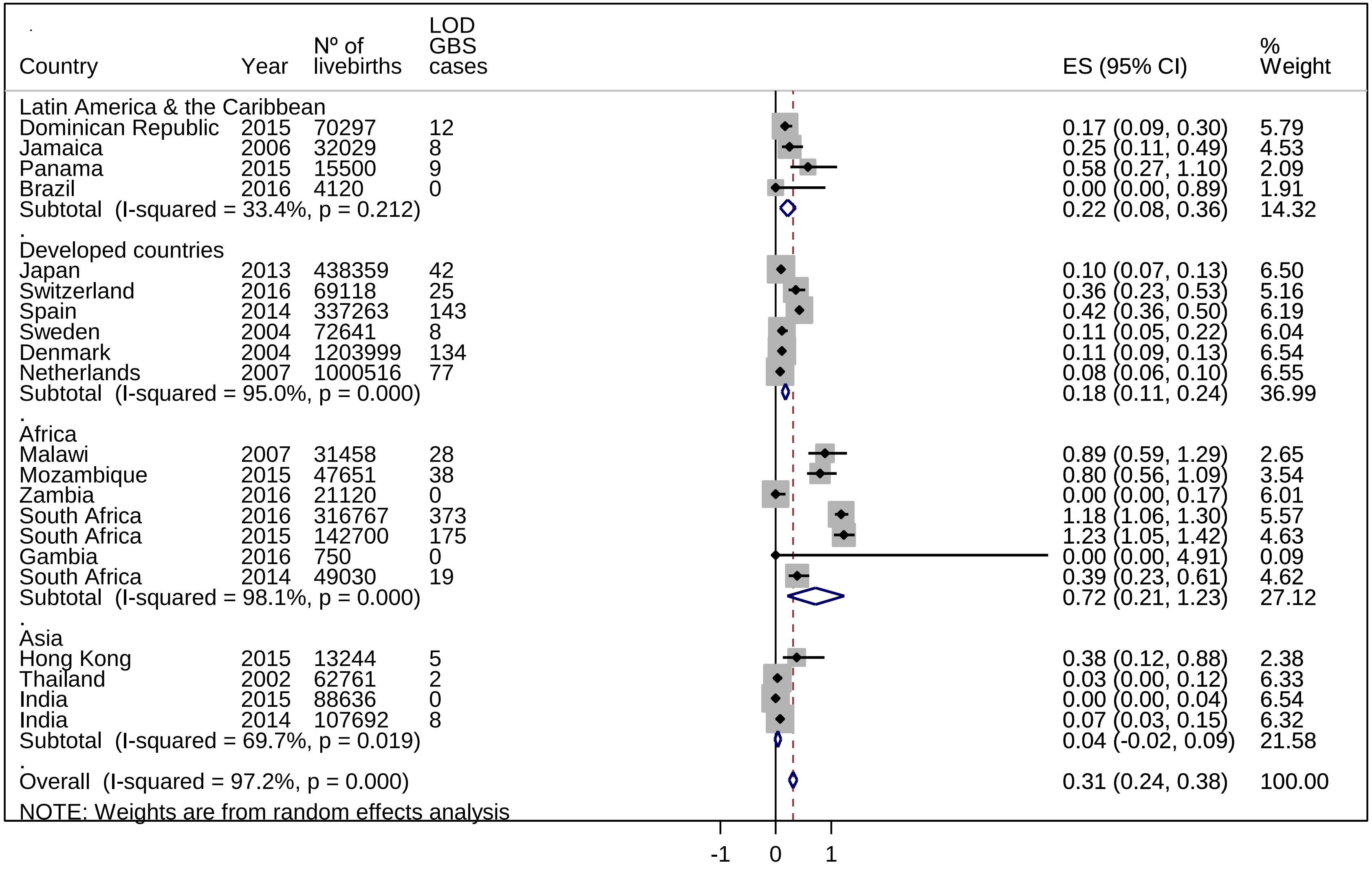

# Supplementary Figure S19: Incidence of early-onset of GBS disease among infants aged 0-6 days by region*

| Country | Year | Nº of livebirths | EOD GBS cases | ES (95% CI) | % Weight |
|---|---|---|---|---|---|
| **Latin America & the Caribbean** | | | | | |
| Jamaica | 2006 | 32029 | 21 | 0.66 (0.41, 1.00) | 2.19 |
| Dominican Republic | 2015 | 17867 | 42 | 2.35 (1.69, 3.18) | 0.72 |
| Panama | 2015 | 15500 | 12 | 0.77 (0.40, 1.35) | 1.36 |
| Argentina | 2005 | 1784 | 0 | 0.00 (0.00, 2.07) | 0.41 |
| Brazil | 2016 | 4120 | 0 | 0.00 (0.00, 0.89) | 1.47 |
| Argentina | 2006 | 18444 | 5 | 0.27 (0.09, 0.63) | 2.34 |
| Subtotal (I-squared = 85.6%, p = 0.000) | | | | 0.65 (0.17, 1.13) | 8.50 |
| **Developed countries** | | | | | |
| Denmark | 2004 | 80263 | 52 | 0.65 (0.48, 0.85) | 2.89 |
| Denmark | 2014 | 33616 | 48 | 1.43 (1.05, 1.89) | 1.58 |
| Spain | 2008 | 107021 | 42 | 0.39 (0.28, 0.53) | 3.23 |
| Australia | 2015 | 847783 | 88 | 0.10 (0.08, 0.13) | 3.58 |
| Norway | 2016 | 168877 | 53 | 0.31 (0.24, 0.41) | 3.40 |
| Japan | 2013 | 438359 | 36 | 0.08 (0.06, 0.11) | 3.57 |
| USA | 2014 | 434908 | 113 | 0.26 (0.21, 0.31) | 3.53 |
| New Zealand | 2016 | 127134 | 29 | 0.23 (0.15, 0.33) | 3.40 |
| Germany | 2006 | 1454520 | 206 | 0.14 (0.12, 0.16) | 3.58 |
| Israel | 2016 | 108000 | 17 | 0.16 (0.09, 0.25) | 3.43 |
| Switzerland | 2016 | 69118 | 8 | 0.12 (0.05, 0.23) | 3.40 |
| UK & Ireland | 2015 | 914132 | 517 | 0.57 (0.52, 0.62) | 3.53 |
| Portugal | 2008 | 448531 | 194 | 0.43 (0.37, 0.50) | 3.49 |
| Sweden | 2004 | 72641 | 42 | 0.58 (0.42, 0.78) | 2.90 |
| France | 2010 | 20131 | 15 | 0.75 (0.42, 1.23) | 1.64 |
| Denmark | 2004 | 1203999 | 338 | 0.28 (0.25, 0.31) | 3.57 |
| Netherlands | 2007 | 1000516 | 353 | 0.35 (0.32, 0.39) | 3.56 |
| Subtotal (I-squared = 97.5%, p = 0.000) | | | | 0.33 (0.26, 0.41) | 54.29 |
| **Africa** | | | | | |
| Malawi | 2007 | 31458 | 29 | 0.92 (0.62, 1.32) | 1.89 |
| Mozambique | 2015 | 47651 | 19 | 0.40 (0.24, 0.62) | 2.84 |
| Kenya | 2016 | 6598 | 5 | 0.76 (0.25, 1.77) | 0.69 |
| Tunisia | 2008 | 11201 | 11 | 0.98 (0.49, 1.76) | 0.92 |
| South Africa | 2016 | 316767 | 447 | 1.41 (1.28, 1.55) | 3.19 |
| South Africa | 2015 | 142700 | 214 | 1.50 (1.31, 1.71) | 2.76 |
| Nigeria | 2005 | 4135 | 1 | 0.24 (0.01, 1.35) | 0.85 |
| Gambia | 2016 | 750 | 1 | 1.33 (0.03, 7.41) | 0.04 |
| South Africa | 2014 | 49030 | 14 | 0.29 (0.16, 0.48) | 3.02 |
| Subtotal (I-squared = 95.4%, p = 0.000) | | | | 0.83 (0.40, 1.27) | 16.20 |
| **Asia** | | | | | |
| China | 2015 | 10924 | 2 | 0.18 (0.02, 0.66) | 2.07 |
| Hong Kong | 2015 | 13244 | 10 | 0.76 (0.36, 1.39) | 1.24 |
| Taiwan | 2016 | 11123 | 0 | 0.00 (0.00, 0.33) | 3.00 |
| Philippines | 2015 | 11768 | 3 | 0.25 (0.05, 0.74) | 1.93 |
| Thailand | 2002 | 62761 | 17 | 0.27 (0.16, 0.43) | 3.16 |
| Thailand | 2015 | 8409 | 2 | 0.24 (0.03, 0.86) | 1.60 |
| Vietnam | 2013 | 39529 | 5 | 0.13 (0.04, 0.30) | 3.22 |
| Bangladesh | 2016 | 19230 | 5 | 0.26 (0.08, 0.61) | 2.41 |
| India | 2016 | 1416 | 2 | 1.41 (0.17, 5.09) | 0.08 |
| Bangladesh | 2009 | 10000 | 1 | 0.10 (0.00, 0.56) | 2.31 |
| Subtotal (I-squared = 33.6%, p = 0.139) | | | | 0.19 (0.09, 0.28) | 21.01 |
| Overall (I-squared = 96.6%, p = 0.000) | | | | 0.42 (0.35, 0.49) | 100.00 |

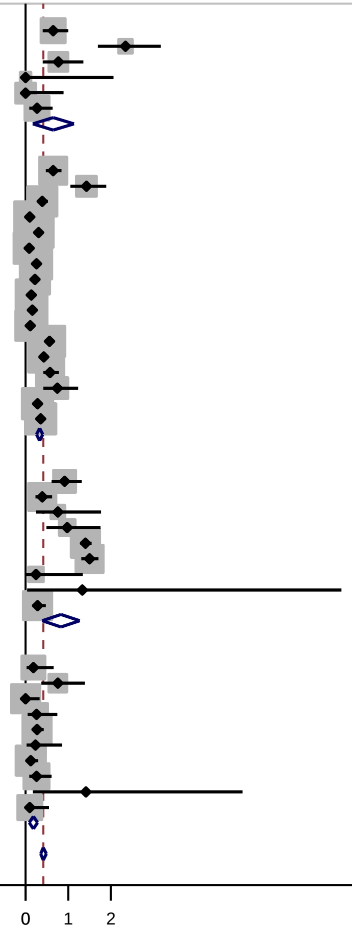


NOTE: Weights are from random effects analysis

*Analysis was restricted to those studies reporting GBS early-onset incidence for complete period (infants aged 0-6 days).

## Supplementary Figure S20: Incidence of late-onset of GBS disease among infants aged 7-89 days by regions*

| Country | Year | Nº of livebirths | LOD GBS cases | ES (95% CI) | % Weight |
|---|---|---|---|---|---|
| **Latin America & the Caribbean** | | | | | |
| Dominican Republic | 2015 | 70297 | 12 | 0.17 (0.09, 0.30) | 10.05 |
| Panama | 2015 | 15500 | 9 | 0.58 (0.27, 1.10) | 5.10 |
| Subtotal (I-squared = 71.2%, p = 0.062) | | | | 0.32 (-0.06, 0.71) | 15.15 |
| . | | | | | |
| **Developed countries** | | | | | |
| Australia | 2015 | 847783 | 62 | 0.07 (0.06, 0.09) | 10.73 |
| Japan | 2013 | 438359 | 42 | 0.10 (0.07, 0.13) | 10.69 |
| USA | 2014 | 434908 | 136 | 0.31 (0.26, 0.37) | 10.56 |
| Italy | 2013 | 311893 | 100 | 0.32 (0.26, 0.39) | 10.48 |
| UK & Ireland | 2015 | 914132 | 339 | 0.37 (0.33, 0.41) | 10.65 |
| Subtotal (I-squared = 98.4%, p = 0.000) | | | | 0.23 (0.11, 0.36) | 53.11 |
| . | | | | | |
| **Africa** | | | | | |
| Mozambique | 2015 | 47651 | 38 | 0.80 (0.56, 1.09) | 7.44 |
| South Africa | 2016 | 316767 | 373 | 1.18 (1.06, 1.30) | 9.84 |
| South Africa | 2014 | 49030 | 19 | 0.39 (0.23, 0.61) | 8.82 |
| Subtotal (I-squared = 96.0%, p = 0.000) | | | | 0.79 (0.26, 1.32) | 26.11 |
| . | | | | | |
| **Asia** | | | | | |
| Hong Kong | 2015 | 13244 | 5 | 0.38 (0.12, 0.88) | 5.63 |
| Subtotal (I-squared = .%, p = .) | | | | 0.38 (-0.00, 0.76) | 5.63 |
| . | | | | | |
| Overall (I-squared = 98.2%, p = 0.000) | | | | 0.40 (0.27, 0.53) | 100.00 |

NOTE: Weights are from random effects analysis

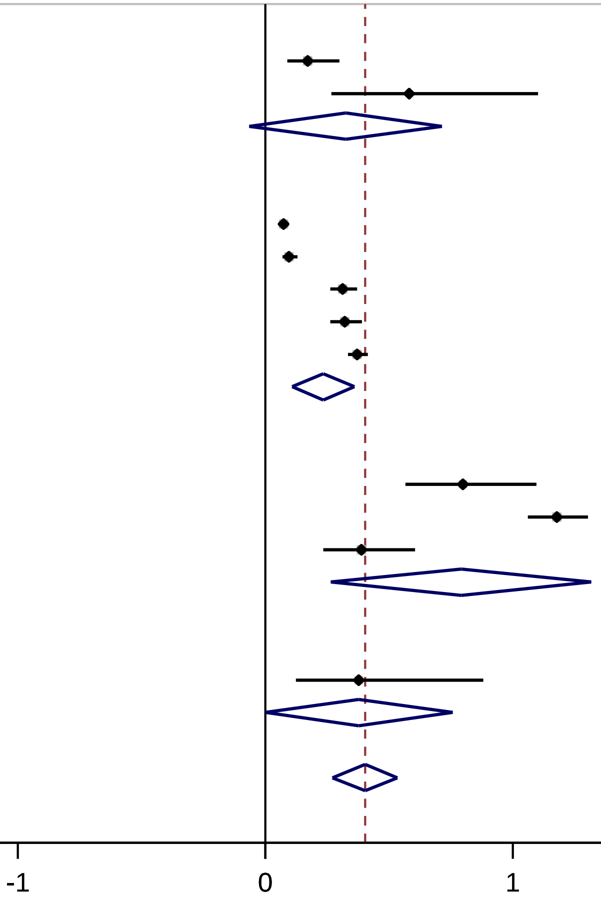


*Analysis was restricted to those studies reporting GBS late-onset incidence for complete period (infants aged 7-89 days).

Supplementary Figure S21: Incidence of late-onset of GBS disease among infants aged 7-27 days by country

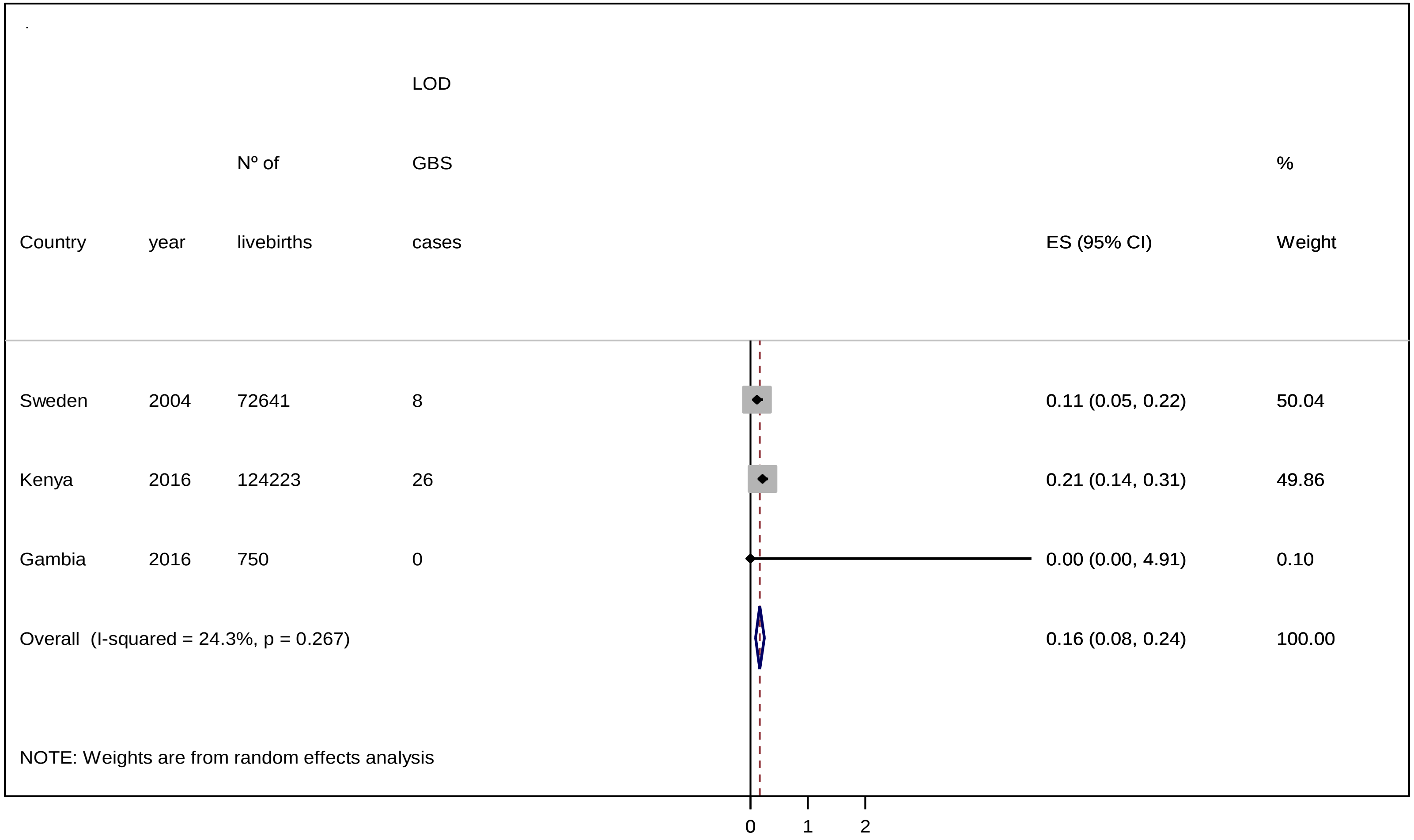


*Analysis was restricted to those studies reporting GBS late-onset incidence in infants aged 7-27 days

## REFERENCES


1. Edmond KM, Kortsalioudaki C, Scott S, et al. Group B streptococcal disease in infants aged younger than 3 months: systematic review and meta-analysis. Lancet **2012**; 379(9815): 547-56.
2. Andersen J, Christensen R, Hertel J. Clinical features and epidemiology of septicaemia and meningitis in neonates due to Streptococcus agalactiae in Copenhagen County, Denmark: a 10 year survey from 1992 to 2001. Acta Paediatr **2004**; 93(10): 1334-9.
3. Bell Y ea. Neonatal sepsis in Jamaican neonates. Ann Trop Paediatr **2005**; 25(4): 293-6.
4. Carbonell-Estrany X, Figueras-Aloy J, Salcedo-Abizanda S, de la Rosa-Fraile M. Probable early-onset group B streptococcal neonatal sepsis: a serious clinical condition related to intrauterine infection. Arch Dis Child Fetal Neonatal Ed **2008**; 93(2): F85-9.
5. Chang CJ, Chang WN, Huang LT, et al. Neonatal bacterial meningitis in southern Taiwan. Pediatr Neurol **2003**; 29(4): 288-94.
6. Davies HD, Raj S, Adair C, Robinson J, McGeer A. Population-based active surveillance for neonatal group B streptococcal infections in Alberta, Canada: implications for vaccine formulation. Pediatr Infect Dis J **2001**; 20(9): 879-84.
7. Ekelund K, Konradsen HB. Invasive group B streptococcal disease in infants: a 19-year nationwide study. Serotype distribution, incidence and recurrent infection. Epidemiol Infect **2004**; 132(6): 1083-90.
8. Fluegge K, Siedler A, Heinrich B, et al. Incidence and clinical presentation of invasive neonatal group B streptococcal infections in Germany. Pediatrics **2006**; 117(6): e1139-45.
9. Hasseltvedt V, et.al. Systemic streptococcal group B disease in Norway-an increasing health problem. Euro Surveill **2001**; 5(40): 2086.
10. Janek Lea. Screening of Hemolytical Streptococcus of Group B in Pregnancy and Prevention of Infection in Newborns. Ceska Gynekologie **2004**; 69(2): 91-4.
11. Jiang JH, Chiu NC, Huang FY, et al. Neonatal sepsis in the neonatal intensive care unit: characteristics of early versus late onset. J Microbiol Immunol Infect **2004**; 37(5): 301-6.
12. Kim JS, Jang YT, Kim JD, et al. Incidence of Haemophilus influenzae type b and other invasive diseases in South Korean children. Vaccine **2004**; 22(29-30): 3952-62.
13. Neto MT. Group B streptococcal disease in Portuguese infants younger than 90 days. Arch Dis Child Fetal Neonatal Ed **2008**; 93(2): F90-3.
14. Niduvaje K, Amutha C, Roy J. Early neonatal streptococcal infection. Indian J Pediatr **2006**; 73(7): 573-6.
15. Ben Hamida Nouaili E, Harouni M, Chaouachi S, Sfar R, Marrakchi Z. [Early-onset neonatal bacterial infections: a retrospective series of 144 cases]. Tunis Med **2008**; 86(2): 136-9.
16. Trijbels-Smeulders M, de Jonge GA, Pasker-de Jong PC, et al. Epidemiology of neonatal group B streptococcal disease in the Netherlands before and after introduction of guidelines for prevention. Arch Dis Child Fetal Neonatal Ed **2007**; 92(4): F271-6.
17. Trotman H, Bell Y. Neonatal group B streptococcal infection at the University Hospital of the West Indies, Jamaica: a 10-year experience. Ann Trop Paediatr **2006**; 26(1): 53-7.
18. Yossuck P, Preedisripipat K. Neonatal group B streptococcal infection: incidence and clinical manifestation in Siriraj Hospital. J Med Assoc Thai **2002**; 85 Suppl 2: S479-87.
19. Hoshina K ea. Trend of neonatal group B streptococcal infection during the last 15 years. Pediatr Int **2002**; 44(6): 641-6.
20. Gray KJ, Bennett SL, French N, Phiri AJ, Graham SM. Invasive group B streptococcal infection in infants, Malawi. Emerg Infect Dis **2007**; 13(2): 223-9.
21. Figueira-Coelho J, Ramirez M, Salgado MJ, Melo-Cristino J. Streptococcus agalactiae in a large Portuguese teaching hospital: antimicrobial susceptibility, serotype distribution, and clonal analysis of macrolide-resistant isolates. Microb Drug Resist **2004**; 10(1): 31-6.
22. Hajdu A, Blystad H, Hoiby EA, Klouman E, Schimmer B, Nygard K. Unexpected increase in case fatality of invasive group B streptococcal infections in infants in Norway, January-July 2006. Euro Surveill **2006**; 11(7): E060727 2.

23. Vaciloto E, et.al. A survey of the Incidence of Neonatal Sepsis by Group B Streptococcus During a Decade in a Brazilian Maternity Hospital. The Brazilian journal of infectious diseases : an official publication of the Brazilian Society of Infectious Diseases **2002**; 6(2): 55-62.
24. Strakova L, Motlova J. Active surveillance of early onset disease due to group to group B streptococci in newborns. Indian J Med Res **2004**; 119: 205-7.
25. Park KH, Kim KH, Kang JH, et al. Current status and clinical presentations of invasive neonatal Group B streptococcal infections in Korea. Pediatr Int **2010**; 53(2): 236-9.
26. Matsubara K, Yamamoto G. Invasive group B streptococcal infections in a tertiary care hospital between 1998 and 2007 in Japan. International journal of infectious diseases : IJID : official publication of the International Society for Infectious Diseases **2009**; 13(6): 679-84.
27. Darmstadt GL, Saha SK, Choi Y, et al. Population-based incidence and etiology of community-acquired neonatal bacteremia in Mirzapur, Bangladesh: an observational study. J Infect Dis **2009**; 200(6): 906-15.
28. Al-Zwaini EJ. Neonatal septicaemia in the neonatal care unit, Al-Anbar governorate, Iraq. East Mediterr Health J **2002**; 8(4-5): 509-14.
29. Sundaram V, Kumar P, Dutta S, et al. Blood culture confirmed bacterial sepsis in neonates in a North Indian tertiary care center: changes over the last decade. Jpn J Infect Dis **2009**; 62(1): 46-50.
30. Martin TC, Adamson J, Dickson T, DiGiantomasso E, Nesbitt C. Does group B streptococcal infection contribute significantly to neonatal sepsis in Antigua and Barbuda? West Indian Med J **2007**; 56(6): 498-501.
31. Cho HK, Lee H, Kang JH, et al. The causative organisms of bacterial meningitis in Korean children in 1996-2005. J Korean Med Sci **2010**; 25(6): 895-9.
32. van den Hoogen A, Gerards LJ, Verboon-Maciolek MA, Fleer A, Krediet TG. Long-term trends in the epidemiology of neonatal sepsis and antibiotic susceptibility of causative agents. Neonatology **2009**; 97(1): 22-8.
33. Kuhn P, Dheu C, Bolender C, et al. Incidence and distribution of pathogens in early-onset neonatal sepsis in the era of antenatal antibiotics. Paediatr Perinat Epidemiol **2010**; 24(5): 479-87.
34. Milledge J, et.al. Aetiology of neonatal sepsis in Blantyre, Malawi: 1996-2001. Ann Trop Paediatr **2005**; 25(2): 101-10.
35. Zhao Z, Kong F, Zeng X, Gidding HF, Morgan J, Gilbert GL. Distribution of genotypes and antibiotic resistance genes among invasive Streptococcus agalactiae (group B streptococcus) isolates from Australasian patients belonging to different age groups. Clinical microbiology and infection : the official publication of the European Society of Clinical Microbiology and Infectious Diseases **2008**; 14(3): 260-7.
36. Martins ER, Pessanha MA, Ramirez M, Melo-Cristino J. Analysis of group B streptococcal isolates from infants and pregnant women in Portugal revealing two lineages with enhanced invasiveness. J Clin Microbiol **2007**; 45(10): 3224-9.
37. Trijbels-Smeulders MA, Kimpen JL, Kollee LA, et al. Serotypes, genotypes, and antibiotic susceptibility profiles of group B streptococci causing neonatal sepsis and meningitis before and after introduction of antibiotic prophylaxis. Pediatr Infect Dis J **2006**; 25(10): 945-8.
38. Fluegge K, Supper S, Siedler A, Berner R. Serotype distribution of invasive group B streptococcal isolates in infants: results from a nationwide active laboratory surveillance study over 2 years in Germany. Clin Infect Dis **2005**; 40(5): 760-3.
39. Persson E, Berg S, Trollfors B, et al. Serotypes and clinical manifestations of invasive group B streptococcal infections in western Sweden 1998-2001. Clinical microbiology and infection : the official publication of the European Society of Clinical Microbiology and Infectious Diseases **2004**; 10(9): 791-6.
40. Davies HD, Jones N, Whittam TS, Elsayed S, Bisharat N, Baker CJ. Multilocus sequence typing of serotype III group B streptococcus and correlation with pathogenic potential. J Infect Dis **2004**; 189(6): 1097-102.
41. Bidet P, Brahimi N, Chalas C, Aujard Y, Bingen E. Molecular characterization of serotype III group B-streptococcus isolates causing neonatal meningitis. J Infect Dis **2003**; 188(8): 1132-7.
42. Lopardo HA, Vidal P, Jeric P, et al. Six-month multicenter study on invasive infections due to group B streptococci in Argentina. J Clin Microbiol **2003**; 41(10): 4688-94.
43. El-Said Mf, et.al. Epidemiology of neonatal meningitis in Qatar. Saudi Med J **2002**; 23(7): 789-92.

44. Ojukwu JU, et.al. Neonatal septicemia in high risk babies in South-Eastern Nigeria. JPerinatMed **2005**; 34: 166-72.
45. Tiskumara R, Fakharee SH, Liu CQ, et al. Neonatal infections in Asia. Arch Dis Child Fetal Neonatal Ed **2009**; 94(2): F144-8.
46. Abdelmaaboud M, Mohammed AF, Abdelmaaboud M, Mohammed AF. Universal screening vs. risk-based strategy for prevention of early-onset neonatal Group-B streptococcal disease. Journal of Tropical Pediatrics **2011**; 57(6): 444-50.
47. Al-Taiar A, Hammoud MS, Cuiqing L, et al. Neonatal infections in China, Malaysia, Hong Kong and Thailand. Archives of Disease in Childhood Fetal & Neonatal Edition **2013**; 98(3): F249-55.
48. Bekker V, Bijlsma MW, van de Beek D, et al. Incidence of invasive group B streptococcal disease and pathogen genotype distribution in newborn babies in the Netherlands over 25 years: a nationwide surveillance study. The Lancet Infectious Diseases **2014**; 14(11): 1083-9.
49. Berardi A, Rossi C, Lugli L, et al. Group B streptococcus late-onset disease: 2003-2010. Pediatrics **2013**; 131(2): e361-8.
50. Bromiker R, Ernest N, Meir MB, et al. Correlation of bacterial type and antibiotic sensitivity with maternal antibiotic exposure in early-onset neonatal sepsis.[Erratum appears in Neonatology. 2013;103(1):53]. Neonatology **2013**; 103(1): 48-53.
51. Chang B, Wada A, Hosoya M, et al. Characteristics of group B Streptococcus isolated from infants with invasive infections: a population-based study in Japan. Japanese Journal of Infectious Diseases **2014**; 67(5): 356-60.
52. Cutland CL, Schrag SJ, Thigpen MC, et al. Increased risk for group B Streptococcus sepsis in young infants exposed to HIV, Soweto, South Africa, 2004-2008(1). Emerging Infectious Diseases **2015**; 21(4): 638-45.
53. Cantoni L, Ronfani L, Da Riol R, et al. Physical examination instead of laboratory tests for most infants born to mothers colonized with group B Streptococcus: support for the Centers for Disease Control and Prevention's 2010 recommendations. Journal of Pediatrics **2013**; 163(2): 568-73.
54. Didier C, Streicher MP, Chognot D, et al. Late-onset neonatal infections: incidences and pathogens in the era of antenatal antibiotics. European Journal of Pediatrics **2012**; 171(4): 681-7.
55. Evangelista ML, Freitas FT, Evangelista MLB, Freitas FTdM. Group B streptococcus neonatal infection in an intensive care unit in Brazil: high fatality and missed opportunities for antibiotic prophylaxis. Brazilian Journal of Infectious Diseases **2015**; 19(1): 98-9.
56. Fiolo K, Zanardi CE, Salvadego M, et al. [Infection rate and Streptococcus agalactiae serotypes in samples of infected neonates in the city of Campinas (Sao Paulo), Brazil]. Revista Brasileira de Ginecologia e Obstetricia **2012**; 34(12): 544-9.
57. Giannoni E, Berger C, Stocker M, et al. Incidence and Outcome of Group B Streptococcal Sepsis in Infants in Switzerland. Pediatric Infectious Disease Journal **2016**; 35 (2): 222-4.
58. Gimenez M, Sanfeliu I, Sierra M, et al. Group B streptococcal early-onset neonatal sepsis in the area of Barcelona (2004-2010). Analysis of missed opportunities for prevention. [Spanish]. Enfermedades Infecciosas y Microbiologia Clinica Monografias **2015**; 33 (7): 446-50.
59. Hashavya S, Benenson S, Ergaz-Shaltiel Z, et al. The use of blood counts and blood cultures to screen neonates born to partially treated group B Streptococcus-carrier mothers for early-onset sepsis: is it justified? Pediatric Infectious Disease Journal **2011**; 30(10): 840-3.
60. Juncosa-Morros T, Guardia-Llobet C, Bosch-Mestres J, et al. Streptococcus agalactiae late-onset neonatal infections in Barcelona (1996-2010). [Spanish]. Enfermedades Infecciosas y Microbiologia Clinica Monografias **2014**; 32 (9): 574-8.
61. Kruse AY, Thieu Chuong do H, Phuong CN, et al. Neonatal bloodstream infections in a pediatric hospital in Vietnam: a cohort study. Journal of Tropical Pediatrics **2013**; 59(6): 483-8.
62. Liu H, Zeng H, Wang W, et al. Estimating the burden of invasive Group B Streptococcal disease in young infants in southern mainland China: An observational study. International Journal of Clinical and Experimental Medicine **2015**; 8 (8): 13699-707.
63. Matsubara K, Hoshina K, Suzuki Y, Matsubara K, Hoshina K, Suzuki Y. Early-onset and late-onset group B streptococcal disease in Japan: a nationwide surveillance study, 2004-2010. International Journal of Infectious Diseases **2013**; 17(6): e379-84.

64. Miyata A, Takahashi H, Kubo T, et al. Early-onset group B streptococcal disease following culture-based screening in Japan: A single center study. Journal of Obstetrics and Gynaecology Research **2012**; 38 (8): 1052-6.
65. Oladottir GL, Erlendsdottir H, Palsson G, et al. Increasing incidence of late-onset neonatal invasive group B streptococcal infections in Iceland. Pediatric Infectious Disease Journal **2011**; 30(8): 661-3.
66. Petersen KB, Johansen HK, Rosthoj S, Krebs L, Pinborg A, Hedegaard M. Increasing prevalence of group B streptococcal infection among pregnant women. Danish Medical Journal **2014**; 61(9): 2014.
67. Sakata H, Sakata H. Pediatric invasive streptococcal infection in northern and eastern regions of Hokkaido, Japan from 2010 to 2012. Pediatrics International **2014**; 56(3): 360-3.
68. Sridhar S, Grace R, Nithya PJ, et al. Group B streptococcal infection in a tertiary hospital in India--1998-2010. Pediatric Infectious Disease Journal **2014**; 33(10): 1091-2.
69. Yu HW, Lin HC, Yang PH, et al. Group B streptococcal infection in Taiwan: maternal colonization and neonatal infection. Pediatrics & Neonatology **2011**; 52(4): 190-5.
70. Thatrimontrichai A, Chanvitan P, Janjindamai W, Dissaneevate S, Jefferies A, Shah V. Trends in neonatal sepsis in a neonatal intensive care unit in Thailand before and after construction of a new facility. Asian Biomedicine **2014**; 8 (6): 771-8.
71. Ko DW, Zurynski Y, Gilbert GL. Group B streptococcal disease and genotypes in Australian infants. J Paediatr Child Health **2015**; 51(8): 808-14.
72. Morozumi M, Wajima T, Kuwata Y, et al. Associations between capsular serotype, multilocus sequence type, and macrolide resistance in Streptococcus agalactiae isolates from Japanese infants with invasive infections. Epidemiology & Infection **2014**; 142(4): 812-9.
73. Almeida A, Villain A, Joubrel C, et al. Whole-Genome Comparison Uncovers Genomic Mutations between Group B Streptococci Sampled from Infected Newborns and Their Mothers. Journal of Bacteriology **2015**; 197(20): 3354-66.
74. Brzychczy-Wloch M, Gosiewski T, Bulanda M, Brzychczy-Wloch M, Gosiewski T, Bulanda M. Multilocus sequence types of invasive and colonizing neonatal group B streptococci in Poland. Medical Principles & Practice **2014**; 23(4): 323-30.
75. Fluegge K, Wons J, Spellerberg B, et al. Genetic differences between invasive and noninvasive neonatal group B streptococcal isolates. Pediatric Infectious Disease Journal **2011**; 30(12): 1027-31.
76. Imperi M, Gherardi G, Berardi A, et al. Invasive neonatal GBS infections from an area-based surveillance study in Italy. Clinical Microbiology & Infection **2011**; 17(12): 1834-9.
77. Joubrel C, Tazi A, Six A, et al. Group B streptococcus neonatal invasive infections, France 2007-2012. Clinical Microbiology and Infection **2015**; 21 (10): 910-6.
78. Six A, Firon A, Plainvert C, et al. Molecular characterization of nonhemolytic and nonpigmented group b streptococci responsible for human invasive infections. Journal of Clinical Microbiology **2016**; 54 (1): 75-82.
79. Souza VC, Kegele FC, Souza SR, et al. Antimicrobial susceptibility and genetic diversity of Streptococcus agalactiae recovered from newborns and pregnant women in Brazil. Scandinavian Journal of Infectious Diseases **2013**; 45(10): 780-5.
80. Teatero S, McGeer A, Low DE, et al. Characterization of invasive group B streptococcus strains from the greater Toronto area, Canada. Journal of Clinical Microbiology **2014**; 52(5): 1441-7.
81. Yoon IA, Jo DS, Cho EY, Choi EH, Lee HJ, Lee H. Clinical significance of serotype V among infants with invasive group B streptococcal infections in South Korea. International Journal of Infectious Diseases **2015**; 38: 136-40.
82. Wang P, Ma Z, Tong J, et al. Serotype distribution, antimicrobial resistance, and molecular characterization of invasive group B Streptococcus isolates recovered from Chinese neonates. International Journal of Infectious Diseases **2015**; 37: e115-e8.
83. Sakata H, Sakata H. Evaluation of intrapartum antibiotic prophylaxis for the prevention of early-onset group B streptococcal infection. Journal of Infection & Chemotherapy **2012**; 18(6): 853-7.
84. Rivera L, Saez-Llorens X, Feris-Iglesias J, et al. Incidence and serotype distribution of invasive group B streptococcal disease in young infants: a multi-country observational study. BMC Pediatr **2015**; 15: 143.

85. Villanueva-Uy ME, Wongsiridej P, Sangtawesin V, et al. THE BURDEN OF INVASIVE NEONATAL GROUP B STREPTOCOCCAL (GBS) DISEASE IN THAILAND AND THE PHILIPPINES. Southeast Asian J Trop Med Public Health **2015**; 46(4): 728-37.
86. Larcher JS, Capellino F, De Giusto R, et al. [Group B streptococcus colonization during pregnancy and prevention of early onset of disease]. Medicina (B Aires) **2005**; 65(3): 201-6.
87. Sigaúque B BQ, Vubil D, Mandomando I, Menendez C, Alonso PL. Invasive bacterial infection among admitted neonates, in Manhiça District Hospital, south of Mozambique. Manhiça, Maputo, Mozambique: Centro de Investigaçao em Saúde de Manhiça., **2011**.
88. Paul VK Agarwal R. Characterisation and antimicrobial resistance of sepsis pathogens in neonates born in tertiary care centres in Delhi, India: a cohort study. Lancet Glob Health **2016**; 4(10): e752-60.
89. Le Doare K, Jarju S, Darboe S, et al. Risk factors for Group B Streptococcus colonisation and disease in Gambian women and their infants. J Infect **2016**; 72(3): 283-94.
90. Saha SK et al. Unpublished. Tangail, Bangladesh, **2016**.
91. Araujo da Silva AR et al. Unpublished. Rio de Janeiro, Brazil, **2016**.
92. Dhaded SM et al. Unpublished. Karnataka South India: J N Medical College and KLE Dr P K Hospital Belagavi, **2016**.
93. Dangor Z, Cutland CL, Izu A, et al. Temporal Changes in Invasive Group B Streptococcus Serotypes: Implications for Vaccine Development. PLoS One **2016**; 11(12): e0169101.
94. Centers for Disease Control and Prevention (CDC). Active Bacterial Core Surveillance (ABCs) Report. . In: Streptococcus. EIPNGB, Vol. 2016, **2014**.
95. O'Sullivan CP LT, Patel D, Efstratiou A , Cunney R , Ladhani S , Reynolds A , Campbell R , Doherty L , Davies E , Jones C , Heath P.T. . Group B Streptococcal (GBS) disease in UK and Irish infants younger than 90 days, 2014-2015. **2017**.
96. Seale AC, Koech AC, Sheppard AE, et al. Maternal colonization with Streptococcus agalactiae and associated stillbirth and neonatal disease in coastal Kenya. Nature microbiology **2016**; 1(7): 16067.
97. Alhhazmi A, Hurteau D, Tyrrell GJ. Epidemiology of Invasive Group B Streptococcal Disease in Alberta, Canada, from 2003 to 2013. J Clin Microbiol **2016**; 54(7): 1774-81.
98. Barbosa NG, Dos Reis H, Mantese OC, Mussi-Pinhata MM, Abdallah VO, Gontijo Filho PP. Early-onset neonatal sepsis by Group B Streptococcus in a Brazilian public hospital. The Brazilian journal of infectious diseases : an official publication of the Brazilian Society of Infectious Diseases **2016**; 20(6): 647-8.
99. Bartlett AW, Smith B, George CR, et al. Epidemiology of Late and Very Late Onset Group B Streptococcal Disease: Fifteen-Year Experience From Two Australian Tertiary Pediatric Facilities. Pediatr Infect Dis J **2017**; 36(1): 20-4.
100. Berardi A, Baroni L, Bacchi Reggiani ML, et al. The burden of early-onset sepsis in Emilia-Romagna (Italy): a 4-year, population-based study. The journal of maternal-fetal & neonatal medicine : the official journal of the European Association of Perinatal Medicine, the Federation of Asia and Oceania Perinatal Societies, the International Society of Perinatal Obstet **2016**; 29(19): 3126-31.
101. Bulkowstein S, Ben-Shimol S, Givon-Lavi N, Melamed R, Shany E, Greenberg D. Comparison of early onset sepsis and community-acquired late onset sepsis in infants less than 3 months of age. BMC Pediatr **2016**; 16: 82.
102. Campisi E, Rosini R, Ji W, et al. Genomic Analysis Reveals Multi-Drug Resistance Clusters in Group B Streptococcus CC17 Hypervirulent Isolates Causing Neonatal Invasive Disease in Southern Mainland China. Frontiers in microbiology **2016**; 7: 1265.
103. Darlow BA, Voss L, Lennon DR, Grimwood K. Early-onset neonatal group B streptococcus sepsis following national risk-based prevention guidelines. The Australian & New Zealand journal of obstetrics & gynaecology **2016**; 56(1): 69-74.
104. Fjalstad JW, Stensvold HJ, Bergseng H, et al. Early-onset Sepsis and Antibiotic Exposure in Term Infants: A Nationwide Population-based Study in Norway. Pediatr Infect Dis J **2016**; 35(1): 1-6.
105. Freitas FT, Romero GA. Early-onset neonatal sepsis and the implementation of group B streptococcus prophylaxis in a Brazilian maternity hospital: a descriptive study. The Brazilian journal of infectious diseases : an official publication of the Brazilian Society of Infectious Diseases **2017**; 21(1): 92-7.

106. Ip M, Ang I, Fung K, Liyanapathirana V, Luo MJ, Lai R. Hypervirulent Clone of Group B Streptococcus Serotype III Sequence Type 283, Hong Kong, 1993-2012. Emerg Infect Dis **2016**; 22(10): 1800-3.
107. Hammoud MS, Al-Taiar A, Al-Abdi SY, et al. Culture-proven early-onset neonatal sepsis in Arab states in the Gulf region: two-year prospective study. International journal of infectious diseases : IJID : official publication of the International Society for Infectious Diseases **2017**; 55: 11-5.
108. Kang HM, Lee HJ, Lee H, et al. Genotype Characterization of Group B Streptococcus Isolated from Infants with Invasive Diseases in South Korea. Pediatr Infect Dis J **2017**.
109. Li YP, Kuok CM, Lin SY, Hsieh WS, Shyu MK. Group B streptococcus antimicrobial resistance in neonates born to group B streptococcus-colonized mothers: Single-center survey. The journal of obstetrics and gynaecology research **2016**; 42(11): 1471-5.
110. Lomuto CQ, Marta; Nigri, Carolina; Fatur, Daniel; Messina, Analía; Santos, Norberto. [Approach to the epidemiologic situation in Argentina of the perinatal infection by beta hemolytic streptococcus of the B group (EGB) ]. Rev Hosp Matern Infant Ramon Sarda **2006**; 25(1): 13-9.
111. Mendoza OM, Arias M, Mendoza L,. [Sepsis neonatal Group B Streptococcus and gram negative bacteria: prevalence and risk of meningitis]. Rev chil pediatr **2013**; 84(5).
112. Martinez MA, Ovalle A, Duran C, et al. [Serotypes and antimicrobial susceptibility of Streptococcus agalactiae]. Revista medica de Chile **2004**; 132(5): 549-55.
113. Poliquin V, Cohen E, Poliquin PG, Schneider C, Menticoglou S. Ongoing Cases of Early-Onset Group B Streptococcal Disease in the Era of Screening and Prophylaxis. Journal of obstetrics and gynaecology Canada : JOGC = Journal d'obstetrique et gynecologie du Canada : JOGC **2016**; 38(10): 926-9.
114. Reinheimer C, Kempf VA, Wittekindt BE, et al. Group B streptococcus infections in neonates admitted to a German NICU: Emphasis on screening and adherence to pre-analytical recommendations. Early Hum Dev **2016**; 103: 37-41.
115. Zeng SJ, Tang XS, Zhao WL, Qiu HX, Wang H, Feng ZC. Clinical analysis of cases of neonatal Streptococcus agalactiae sepsis. Genetics and molecular research : GMR **2016**; 15(2).
116. Tapia IJ, Reichhard TC, Saldias RM, et al. [Neonatal sepsis in the era of antenatal antibiotic prophylaxis]. Revista chilena de infectologia : organo oficial de la Sociedad Chilena de Infectologia **2007**; 24(2): 111-6.
117. Kabwe M, Tembo J, Chilukutu L, et al. Etiology, Antibiotic Resistance and Risk Factors for Neonatal Sepsis in a Large Referral Center in Zambia. Pediatr Infect Dis J **2016**; 35(7): e191-8.
118. Akindolire AE, Tongo O, Dada-Adegbola H, Akinyinka O. Etiology of early onset septicemia among neonates at the University College Hospital, Ibadan, Nigeria. J Infect Dev Ctries **2016**; 10(12): 1338-44.
119. Ovalle A, Levancini M. Urinary tract infections in pregnancy. Curr Opin Urol **2001**; 11(1): 55-9.
120. Delgado Picado E; Saenz-Sanchez C; Calderon Zumiga A. [Rate of Colonization of Streptococcus Agalactiae in Pregnant Women and Neonates, Women's Hospital Dr Adolfo Carit Eva]. Rev costarric cienc méd **2004**; 25(1): 25-32.
121. Díaz-Álvarez M CD, Pérez-Amarillo J. [Infections due to Streptococcus agalactiae at an open neonatology service ]. Rev Cubana Pediatr **2008**; 80(4).
122. Costa N; Carvalho M; Moura S; Júnior SC. [Beta-hemolytic streptococcus in pregnant women and their newborn infants: a critical analysis of the protocol used at Fernandes Figueira Institute, Oswaldo Cruz Foundation, in Brazil]. Rev paul pediatr **2010**; 28(2).
123. Frigati L, van der Merwe JL, Harvey J, Rabie H, Theron G, Cotton MF. A retrospective review of group B streptococcal infection in the Metro East area of the Western Cape province: 2010 to 2011. Southern African Journal of Infectious Diseases **2014**; 29(1): 33-6.
124. Angstetra D, Ferguson J, Giles WB. Institution of universal screening for Group B streptococcus (GBS) from a risk management protocol results in reduction of early-onset GBS disease in a tertiary obstetric unit. The Australian & New Zealand journal of obstetrics & gynaecology **2007**; 47(5): 378-82.
125. Ireland S, Larkins S, Kandasamy Y, Ireland S, Larkins S, Kandasamy Y. Group B streptococcal infection in the first 90 days of life in North Queensland. Australian & New Zealand Journal of Obstetrics & Gynaecology **2014**; 54(2): 146-51.
126. Daley AJ, Isaacs D. Ten-year study on the effect of intrapartum antibiotic prophylaxis on early onset group B streptococcal and Escherichia coli neonatal sepsis in Australasia. Pediatr Infect Dis J **2004**; 23(7): 630-4.

127. May M, Daley AJ, Donath S, Isaacs D. Early onset neonatal meningitis in Australia and New Zealand, 1992-2002. Arch Dis Child Fetal Neonatal Ed **2005**; 90(4): F324-7.
128. Meehan M, Cunney R, Cafferkey M, Meehan M, Cunney R, Cafferkey M. Molecular epidemiology of group B streptococci in Ireland reveals a diverse population with evidence of capsular switching. European Journal of Clinical Microbiology & Infectious Diseases **2014**; 33(7): 1155-62.
129. Berardi A, Lugli L, Baronciani D, et al. Group B streptococcal infections in a northern region of Italy. Pediatrics **2007**; 120(3): e487-93.
130. Berardi A, Lugli L, Rossi C, et al. Intrapartum antibiotic prophylaxis failure and group-B streptococcus early-onset disease. Journal of Maternal-Fetal & Neonatal Medicine **2011**; 24(10): 1221-4.
131. Berardi A, Lugli L, Rossi C, et al. Impact of perinatal practices for early-onset group B Streptococcal disease prevention. Pediatr Infect Dis J **2013**; 32(7): e265-71.
132. Berkley JA, Lowe BS, Mwangi I, et al. Bacteremia among children admitted to a rural hospital in Kenya. N Engl J Med **2005**; 352(1): 39-47.
133. Sigauque B, Roca A, Mandomando I, et al. Community-acquired bacteremia among children admitted to a rural hospital in Mozambique. Pediatr Infect Dis J **2009**; 28(2): 108-13.
134. Eastwood KA, Craig S, Sidhu H, et al. Prevention of early-onset Group B Streptococcal disease - the Northern Ireland experience. BJOG: An International Journal of Obstetrics & Gynaecology **2015**; 122(3): 361-7.
135. Madhi SA, Radebe K, Crewe-Brown H, et al. High burden of invasive Streptococcus agalactiae disease in South African infants. Ann Trop Paediatr **2003**; 23(1): 15-23.
136. Cutland CL, Madhi SA, Zell ER, et al. Chlorhexidine maternal-vaginal and neonate body wipes in sepsis and vertical transmission of pathogenic bacteria in South Africa: a randomised, controlled trial. Lancet **2009**; 374(9705): 1909-16.
137. Cutland CL, Schrag SJ, Zell ER, et al. Maternal HIV infection and vertical transmission of pathogenic bacteria. Pediatrics **2012**; 130(3): e581-90.
138. Dangor Z, Lala SG, Cutland CL, et al. Burden of invasive group B Streptococcus disease and early neurological sequelae in South African infants. PLoS ONE **2015**; 10 (4) (no pagination)(e0123014).
139. Schrag SJ, Cutland CL, Zell ER, et al. Risk factors for neonatal sepsis and perinatal death among infants enrolled in the prevention of perinatal sepsis trial, Soweto, South Africa. Pediatric Infectious Disease Journal **2012**; 31 (8): 821-6.
140. Andreu A, Sanfeliu I, Vinas L, et al. [Decreasing incidence of perinatal group B streptococcal disease (Barcelona 1994-2002). Relation with hospital prevention policies]. Enferm Infecc Microbiol Clin **2003**; 21(4): 174-9.
141. Lopez Sastre JB, Fernandez Colomer B, Coto Cotallo GD, Ramos Aparicio A. Trends in the epidemiology of neonatal sepsis of vertical transmission in the era of group B streptococcal prevention. Acta Paediatr **2005**; 94(4): 451-7.
142. Martins ER, Andreu A, Correia P, et al. Group B streptococci causing neonatal infections in barcelona are a stable clonal population: 18-year surveillance. Journal of Clinical Microbiology **2011**; 49(8): 2911-8.
143. Vergnano S, Menson E, Kennea N, et al. Neonatal infections in England: the NeonIN surveillance network. Arch Dis Child Fetal Neonatal Ed **2010**; 96(1): F9-F14.
144. Lamagni TL, Keshishian C, Efstratiou A, et al. Emerging trends in the epidemiology of invasive group B streptococcal disease in England and Wales, 1991-2010. Clinical Infectious Diseases **2013**; 57(5): 682-8.
145. Weisner AM, Johnson AP, Lamagni TL, et al. Characterization of group B streptococci recovered from infants with invasive disease in England and Wales. Clin Infect Dis **2004**; 38(9): 1203-8.
146. Meehan M, Cafferkey M, Corcoran S, et al. Real-time polymerase chain reaction and culture in the diagnosis of invasive group B streptococcal disease in infants: a retrospective study. European Journal of Clinical Microbiology and Infectious Diseases **2015**; 34 (12): 2413-20.
147. Oddie S, Embleton ND. Risk factors for early onset neonatal group B streptococcal sepsis: case-control study. BMJ (Clinical research ed) **2002**; 325(7359): 308.
148. Okike IO, Johnson AP, Henderson KL, et al. Incidence, etiology, and outcome of bacterial meningitis in infants aged <90 days in the United kingdom and Republic of Ireland: prospective, enhanced, national population-based surveillance. Clinical Infectious Diseases **2014**; 59(10): e150-7.

149. Heath PT, Balfour G, Weisner AM, et al. Group B streptococcal disease in UK and Irish infants younger than 90 days. Lancet **2004**; 363(9405): 292-4.
150. Early-onset and late-onset neonatal group B streptococcal disease--United States, 1996-2004. MMWR Morb Mortal Wkly Rep **2005**; 54(47): 1205-8.
151. Castrodale L, Gessner B, Hammitt L, Chimonas MA, Hennessy T. Invasive early-onset neonatal group B streptococcal cases--Alaska, 2000-2004. Matern Child Health J **2007**; 11(1): 91-5.
152. Chen KT, Puopolo KM, Eichenwald EC, Onderdonk AB, Lieberman E. No increase in rates of early-onset neonatal sepsis by antibiotic-resistant group B Streptococcus in the era of intrapartum antibiotic prophylaxis. Am J Obstet Gynecol **2005**; 192(4): 1167-71.
153. Cordero L, Rau R, Taylor D, Ayers LW. Enteric gram-negative bacilli bloodstream infections: 17 years' experience in a neonatal intensive care unit. Am J Infect Control **2004**; 32(4): 189-95.
154. Hyde TB, Hilger TM, Reingold A, Farley MM, O'Brien KL, Schuchat A. Trends in incidence and antimicrobial resistance of early-onset sepsis: population-based surveillance in San Francisco and Atlanta. Pediatrics **2002**; 110(4): 690-5.
155. Mayor-Lynn K, Gonzalez-Quintero VH, O'Sullivan MJ, Hartstein AI, Roger S, Tamayo M. Comparison of early-onset neonatal sepsis caused by Escherichia coli and group B Streptococcus. Am J Obstet Gynecol **2005**; 192(5): 1437-9.
156. Phares CR, Lynfield R, Farley MM, et al. Epidemiology of invasive group B streptococcal disease in the United States, 1999-2005. JAMA **2008**; 299(17): 2056-65.
157. Puopolo KM, Madoff LC, Eichenwald EC. Early-onset group B streptococcal disease in the era of maternal screening. Pediatrics **2005**; 115(5): 1240-6.
158. Perinatal group B streptococcal disease after universal screening recommendations--United States, 2003-2005. MMWR Morb Mortal Wkly Rep **2007**; 56(28): 701-5.
159. Stoll BJ, Hansen N, Fanaroff AA, et al. Changes in pathogens causing early-onset sepsis in very-low-birth-weight infants. N Engl J Med **2002**; 347(4): 240-7.
160. Jordan HT, Farley MM, Craig A, et al. Revisiting the need for vaccine prevention of late-onset neonatal group B streptococcal disease: a multistate, population-based analysis. Pediatr Infect Dis J **2008**; 27(12): 1057-64.
161. Trends in perinatal group B streptococcal disease - United States, 2000-2006. MMWR Morb Mortal Wkly Rep **2009**; 58(5): 109-12.
162. Ecker KL, Donohue PK, Kim KS, Shepard JA, Aucott SW. The impact of group B Streptococcus prophylaxis on early onset neonatal infections. Journal of Neonatal-Perinatal Medicine **2013**; 6(1): 37-44.
163. Greenhow TL, Hung YY, Herz AM, Greenhow TL, Hung Y-Y, Herz AM. Changing epidemiology of bacteremia in infants aged 1 week to 3 months. Pediatrics **2012**; 129(3): e590-6.
164. Greenhow TL, Hung YY, Herz AM, et al. The changing epidemiology of serious bacterial infections in young infants. Pediatric Infectious Disease Journal **2014**; 33(6): 595-9.
165. Mukhopadhyay S, Eichenwald EC, Puopolo KM. Neonatal early-onset sepsis evaluations among well-appearing infants: projected impact of changes in CDC GBS guidelines. Journal of Perinatology **2013**; 33(3): 198-205.
166. Mukhopadhyay S, Dukhovny D, Mao W, et al. 2010 perinatal GBS prevention guideline and resource utilization. Pediatrics **2014**; 133(2): 196-203.
167. Tudela CM, Stewart RD, Roberts SW, et al. Intrapartum evidence of early-onset group B streptococcus. Obstetrics & Gynecology **2012**; 119(3): 626-9.
168. Weston EJ, Pondo T, Lewis MM, et al. The burden of invasive early-onset neonatal sepsis in the United States, 2005-2008. Pediatric Infectious Disease Journal **2011**; 30(11): 937-41.
169. Stafford IA, Stewart RD, Sheffield JS, et al. Efficacy of maternal and neonatal chemoprophylaxis for early-onset group B streptococcal disease. Obstetrics and gynecology **2012**; 120 (1): 123-9.
170. Stoll BJ, Hansen NI, Sanchez PJ, et al. Early Onset Neonatal Sepsis: The Burden of Group B Streptococcal and E. coli Disease Continues. Pediatrics **2011**; 127(5): 817-26.
171. Parente V, Clark RH, Ku L, et al. Risk factors for group B streptococcal disease in neonates of mothers with negative antenatal testing. Journal of perinatology : official journal of the California Perinatal Association **2017**; 37(2): 157-61.

172. Wortham JM, Hansen NI, Schrag SJ, et al. Chorioamnionitis and culture-confirmed, early-onset neonatal infections. Pediatrics **2016**; 137 (1) (no pagination)(e20152323).
